# Designing novel bisquinoline antimalarials from historical 4-aminoquinolines to combat drug-resistant malaria

**DOI:** 10.1128/aac.01300-25

**Published:** 2026-03-02

**Authors:** Mason J. Handford, Yuexin Li, Terry Riscoe, Xiaowei Zhang, Jane X. Kelly, Michael K. Riscoe

**Affiliations:** 1 Chemical Physiology and Biochemistry Department, Oregon Health & Science University 6684, Portland, Oregon, USA; 2 Experimental Chemotherapy Lab, Portland VA Research Foundation 467019, Portland, Oregon, USA; 3 Department of Chemistry, Portland State University 574478, Portland, Oregon, USA; 4 Molecular Microbiology and Immunology Department, Oregon Health & Science University 6684, Portland, Oregon, USA; The Children's Hospital of Philadelphia, Philadelphia, Pennsylvania, USA

**Keywords:** *Plasmodium falciparum*, malaria, multidrug-resistant malaria, 4-aminoquinoline, antimalarial agents

## Abstract

*Plasmodium falciparum,* the deadliest causative agent of malaria, continues to evade eradication efforts through widespread drug resistance. The recent development of **ADC-028**, a 4-aminoquinoline antimalarial with excellent activity and pharmacokinetic properties, prompted the investigation of bisquinoline analogs featuring similar structural motifs. Here, we describe a structure-activity relationship study that guided the optimization of compounds with key features, including the 4-anilinoquinoline core and diverse bridging linkers. Several analogs exhibited potent *in vitro* activity (IC_50_ < 20 nM) against both drug-sensitive and multidrug-resistant *P. falciparum* strains, while maintaining favorable cytotoxicity profiles. Among them, **25** demonstrated improved intrinsic metabolic stability (t_1/2_ = 121 min) and potent *in vivo* efficacy (ED_50_ = 0.32 mg/kg/day), achieving complete curative protection at a reduced dose compared to ADC-028. While **25** showed moderately reduced oral bioavailability (F = 43%) and a shorter half-life (T_1/2_ = 27.2 h) relative to ADC-028, its enhanced *in vivo* efficacy underscores its therapeutic potential. This work highlights a promising path forward in developing antimalarials that retain the efficacy of legacy compounds while overcoming modern resistance mechanisms.

## INTRODUCTION

**Fig 1:**
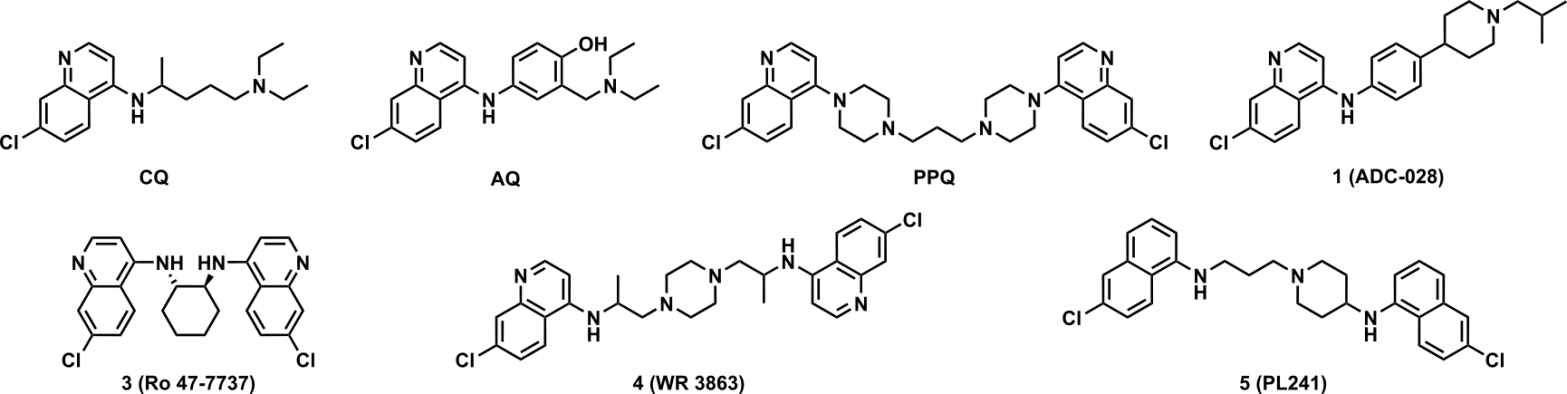
Top: structure representations of 4-aminoquinoline antimalarials (from left to right): CQ, AQ, PPQ, and amodiachin-028 (ADC-028, 1). Bottom: survey of symmetrical and unsymmetrical 4-aminoquinoline compounds from other antimalarial drug discovery programs, including Ro 47-7737 (10, 11) (3), WR 3863 (11) (4), and PL241 (12) (5).

*Plasmodium falciparum* (*Pf*) causes the most severe form of malaria, causing approximately 597,000 deaths in 2023, mostly among children ages 5 and under (1). The parasite’s complex life cycle aids in its evasion of global eradication initiatives and, consequently, has produced strains of parasites that carry resistance to multiple antimalarials. During the blood stage of infection, *Plasmodium* spp. lack the machinery to synthesize essential amino acids and thus acquire them by protease-mediated digestion of host hemoglobin in acidified digestive vacuoles, lysosome-like organelles (2). Cytotoxic heme is released during this process but is detoxified via aggregation into an inert biomolecule, hemozoin (3, 4). Inhibition of this protective pathway is part of the mechanism of action of 4-aminoquinoline antimalarials (Fig. 1) such as chloroquine (**CQ**), amodiaquine (**AQ**), and piperaquine (**PPQ**) (5, 6). Resistance to 4-aminoquinolines results primarily from the accumulation of mutations in the *Pf* CQ resistance transporter (*pfcrt*), which promote 4-aminoquinoline efflux from the digestive vacuole, vastly reducing their antiplasmodial activity (7). CQ-resistant *Pf* strains were first identified in 1957 in Cambodia and quickly spread throughout Asia and into Africa by the 1970s (8). CQ was integral to early eradication and treatment efforts, and as CQ-resistant strains emerged, successful 4-aminoquinoline CQ analogs like AQ and PPQ were developed to be effective against those strains (9). Sadly, resistance to those antimalarials and toxicity issues have put their continued effectiveness at risk. Development of 4-aminoquinoline analogs continues today, aiming to discover antimalarials active against not only CQ-resistant parasites but also multidrug-resistant *Pf*.

Our recent work on 4-aminoquinoline-based analogs led to the development of ADC-028 (**1**). This compound was designed to overcome the toxicity of AQ and the pharmacokinetic (PK) shortcomings of isoquine (9, 13). Compound **1** exhibited nanomolar activity against multidrug-resistant strains of *Pf* (IC_50_ vs *Pf*Dd2 = 17 nM), was non-cytotoxic (CC_50_ vs HepG2 > 20 µM), and was moderately stable *in vitro* (intrinsic metabolic stability, t_1/2_ = 48.2 min). The compound was effective in a mouse malaria model (ED_90_ = 2.5 mg/kg/day) and provided complete cures of infected animals at 16 mg/kg/day in the standard modified Peters 4-Day test and a single-dose cure at 50 mg/kg (14). Its PK profile exhibited an extended bloodstream half-life (T_1/2_ = 84 h) and excellent oral bioavailability (%F = 74). Taking these characteristics together, **1** was identified as the frontrunner of that set of compounds. In addition, the structure-activity relationship (SAR) study from that series highlighted several key structural moieties that could expand SAR profiling into unexplored chemical space. Motivated by both the high therapeutic potential of **1** and the existing precedent for both symmetrical and unsymmetrical bis-4-aminoquinolines (**3–5**) antimalarials (10–12), we decided to investigate a bis-quinoline pharmacophore incorporating components of the aminophenyl piperidine core found in **1**. The results of this investigation are presented below.

## RESULTS AND DISCUSSION

### Rationale and chemistry

**Fig 2:**
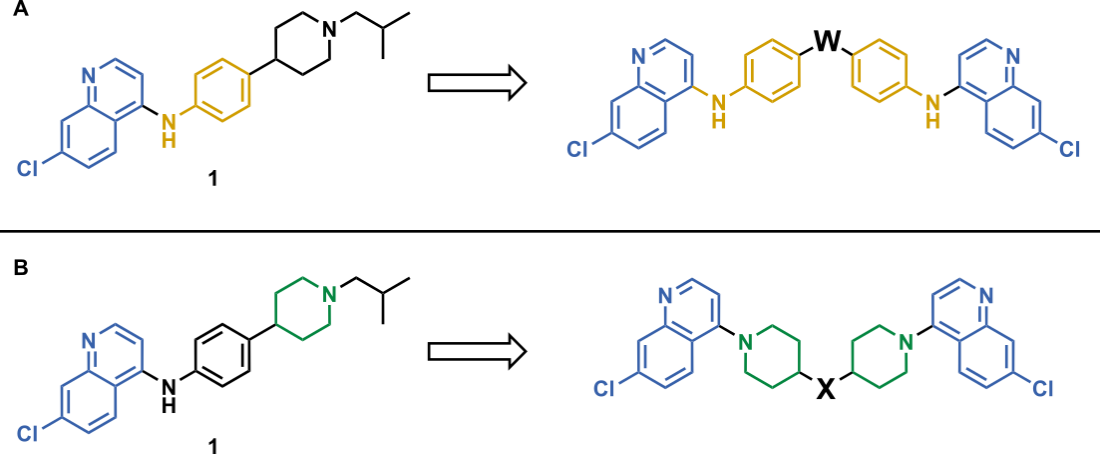
Structure of 1 (ADC-028) with structural variations of interest and Markush structure of bisquinoline designs. (**A**) Compound 1 with the 7-chloro-N-phenylquinolin-4-amine core (highlighted in blue), the aniline moiety (highlighted in yellow) that is directly connected to the quinoline core, and the Markush structure of desired symmetrical bisquinolines that utilize these structural components. (**B**) Compound 1 with the 7-chloro-N-phenylquinolin-4-amine core (blue), in addition to the piperidine moiety (green), and the Markush structure of desired symmetrical bisquinolines that utilize these structural components.

**Fig 3:**
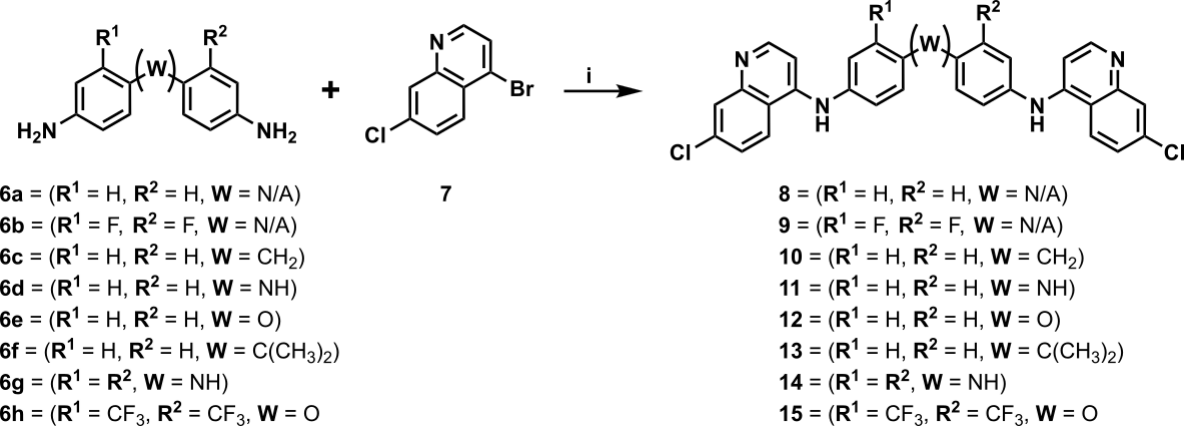
Synthetic scheme used to generate compounds 8–15. Reagents and conditions: (i) linker, 4-bromo-7-chloroquinoline, phenol, DMF, 2 h, 150°C.

**Fig 4:**
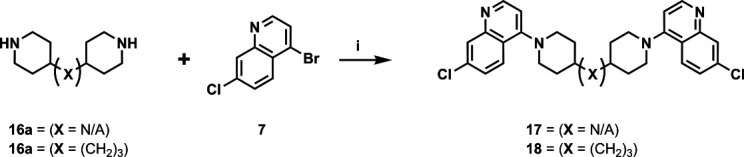
Synthetic scheme used to generate compounds 17 and 18. Reagents and conditions: (i) linker, 4-bromo-7-chloroquinoline, phenol, DMF, 2 h, 150°C.

Our initial design was based on the intrinsic antiplasmodial activity of the 7-chloro-N-phenylquinolin-4-amine core, highlighted in blue (7-chloroquinoline) and gold (aniline) in Fig. 2A, which is shared between **1** and other antiplasmodial compounds (15). We sought to vary the atom **W** between two symmetrical cores (Fig. 2A) utilizing a variety of commercially available bis-anilines. The conditions used in the synthesis of compounds **8–15** are summarized in Fig. 3. Starting material **7** was prepared from commercially available 4-hydro-7-chloroquine using previously published methods (16). Compounds **8–15** were afforded from **7** when reacted with the appropriate bis-aniline and phenol in DMF at 150°C for 2 h in a microwave reactor. Again, we sought to diversify the bisquinoline structure by varying the connecting atom **X** between the two symmetrical bisquinoline cores by utilizing a bis-piperidine moiety in lieu of a bis-aniline, seen in Fig. 2B. Figure 4 illustrates the synthesis of compounds **17** and **18**, utilizing identical reaction conditions as described in Fig. 3.

**Fig 5:**
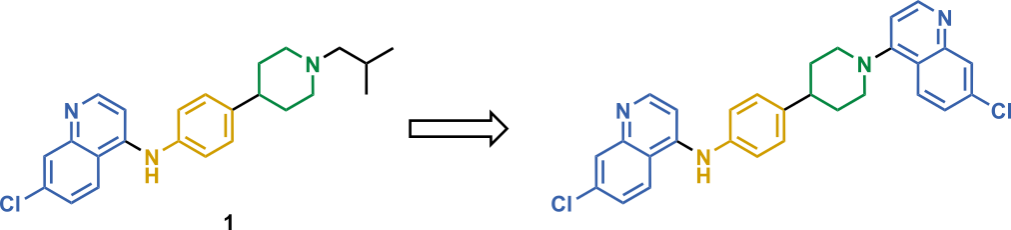
Structure of 1 (left) with moieties of interest and Markush structure of bisquinoline design (right). Compound 1 with the 7-chloro-N-phenylquinolin-4-amine core highlighted in blue, in addition to the aniline moiety in yellow and the piperidine in blue, followed by the Markush structure of possible unsymmetrical bisquinolines that utilize those structural constituents.

**Fig 6:**
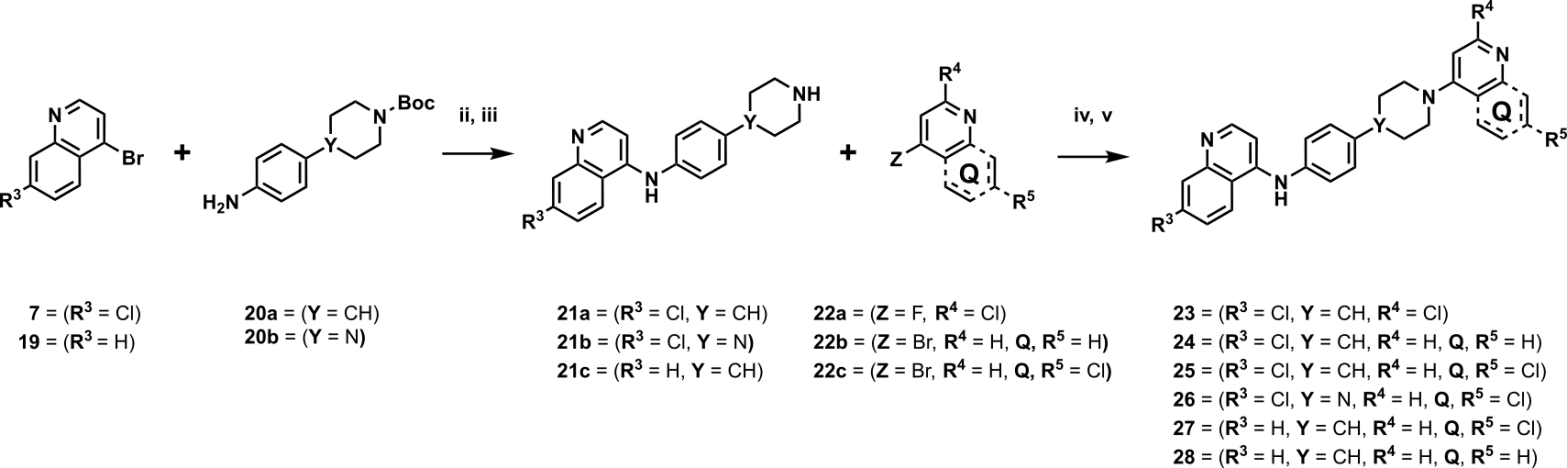
Synthetic scheme used to generate compounds 23–28. Reagents and conditions: (ii) aniline, 4-bromo-7-chloroquinoline, THF, 20 min, 120°C. (iii) TFA, DCM, rt, 2 h. (iv) 4-Chloro-2-fluoropyridine, DIPEA, NMP, 2 h, 150°C. (v) 4-Bromo-7-chloroquinoline, phenol, DMF, 2 h, 150°C.

We next returned to the original inspiration for this compound series and sought to utilize the original structure of **1** but retain both the aniline and piperidine elements and include a second quinoline onto the terminal nitrogen of the piperidine ring, as illustrated in Fig. 5. Compounds **23–28** were afforded using the reaction conditions outlined in Fig. 6. In short, the initial quinoline, with or without the 7-position chlorine atom, was reacted together with a Boc-protected 4-(4-aminophenyl)piperidine or 4-(4-aminophenyl)piperazine in THF at 120°C for 20 min. The solid products were filtered and washed with excess THF and subjected to a short aqueous workup before deprotection in TFA in DCM. The resulting intermediates (**21a–c**) were used in reactions with either 2-chloro-4-fluoropyridine (**22a**) and diisopropylethylamine in N-methyl-2-pyrrolidone at 150°C for 2 h to produce 23, or 4-bromoquinoline with or without a 7-position chlorine atom (**22b** and **22c**, respectively) using similar conditions as previously described to produce compounds **24–28**.

### *In vitro* activity of bisquinolines vs *Pf* and their relative cytotoxicity

Tables 1 and 2 contain compound cytotoxicity (CC_50_) against HepG2 mammalian cells and mean and standard deviations of antiplasmodial activity (IC_50_) determined from a fluorescence-based antiplasmodial activity assay (17). Proven efficacious antimalarials **CQ** and **PPQ** were used as controls to illustrate benchmark cytotoxicity (18, 19) and activity of other 4-aminoquinolines against the drug-sensitive *Pf* D6 (*Pf*D6) strain, as well as to highlight the resistance profile against the multidrug-resistant *Pf* Dd2 (*Pf*Dd2) strain. In Table 1, all compounds used in this assay, except for **9** and **18**, exhibit IC_50_ values lower than 50 nM against both *Pf*D6 and *Pf*Dd2, indicating an elevated level of intrinsic antiplasmodial potency, while simultaneously highlighting the capacity to overcome the resistance mechanisms of the *Pf*Dd2 strain. Compounds **24–28** in Table 2 exhibit similar antiplasmodial activity against both strains of *Pf* with a range of IC_50_ values from 14 nM to 5 nM against *Pf*D6 and 20 nM to 7.4 nM against *Pf*Dd2, regardless of the piperazine- vs piperidine-containing 4-aminoquinolines. The majority of compounds described in Tables 1 and 2 demonstrate promising cytotoxicity profiles, with CC_50_ values exceeding 50 µM. Therefore, to further evaluate this series of compounds for future development, we implemented the selectivity index on a log scale (pSI) to quantify the margin between a compound’s potency and cytotoxicity. Indeed, most of the compounds in Tables 1 and 2 are either highly selective (pSI = 3–4) or very highly selective (pSI > 4), indicating a low probability of cytotoxicity at a therapeutic dose. However, compound **9** has the lowest pSI (< 2.9), and due to activity against the PfD6 strain (IC_50_ ≥ 250 nM) lying outside of our testing range, it is difficult to say how cytotoxic the compound might be. When comparing the CC_50_ values of compounds found in Table 2 that differ only at the 7-position of the 4-aminophenyl quinoline—such as compounds **24** (CC_50_ > 200 µM) and **28** (CC_50_ = 12.8 µM) or compounds **25** (CC_50_ > 200 µM) and **27** (CC_50_ = 11.8 µM)—it is apparent that the 7-position chlorine atom of the 4-aminophenyl quinoline is key to low cytotoxicity. In our pipeline, a CC_50_ ≤ 20 µM serves as a heuristic threshold for identifying toxic analogs in early-stage discovery. Although the cytotoxicity of **14** is near that threshold and **28** and **27** are below it, they are still within an order of magnitude of that of CQ, an FDA-approved antimalarial, in the same assay (CC_50_ = 38 µM). Additionally, each compound’s selectivity index indicates an effective concentration *in vitro* against *Pf*D6 that is more than three orders of magnitude smaller than their potentially cytotoxic dose *in vitro* (CC_50_). Based on this context, we chose to continue evaluating the therapeutic potential of **14**, **27**, and **28**. We interpret these results as support for our initial hypothesis of highly active and non-cytotoxic antimalarials within this chemical space, which in turn stimulated us to further evaluate these compounds for metabolic stability and *in vivo* efficacy.

**TABLE 1:** SAR profiling of bisquinolines vs drug-sensitive (D6) and multidrug-resistant (Dd2) strains of *Pf*: impact of bisquinoline linker on antiplasmodial activity and cytotoxicity^*a*^^,^^*b*^

		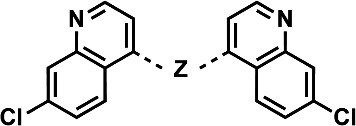			
Compound	Z	IC_50_ vs *Pf*D6 ± SD (nM)	IC_50_ vs *Pf*Dd2 ± SD (nM)	CC_50_ vs HepG2 (µM)	Log selectivity index (pSI)
**CQ**		10 ± 2	106 ± 10	37.8^*c*^	3.6
**PPQ**	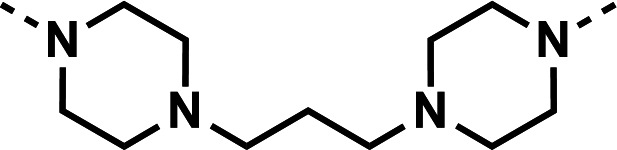	3.8 ± 1.6	5.8 ± 1.8	≥120^*c*^	≥4.5
**8**	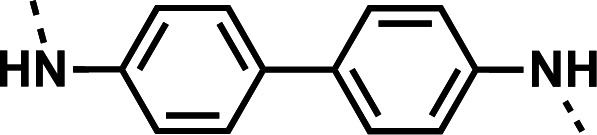	14.6 ± 1.3	10 ± 2	≥200	≥4.1
**9**	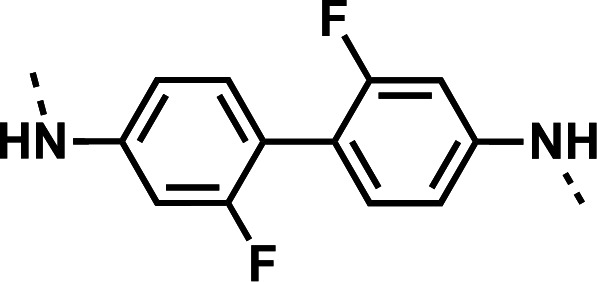	≥250	111 ± 13	≥200	<2.9
**10**	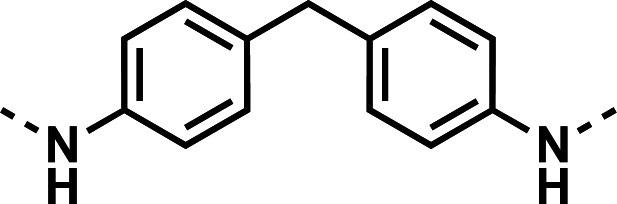	11 ± 4	7 ± 5	≥200	≥4.3
**11**	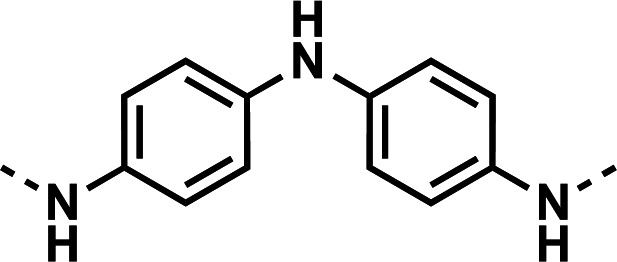	9 ± 6	6.8 ± 1.8	100	4.0
**12**	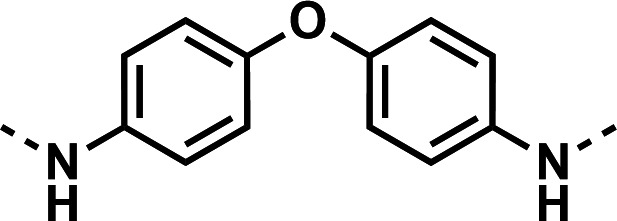	10.4 ± 1.9	7 ± 7	≥200	≥4.3
**13**	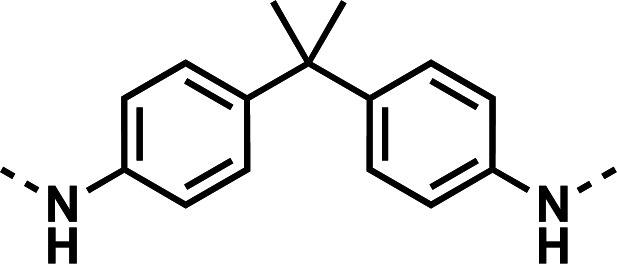	19 ± 10	15 ± 9	96.4	3.7
**14**	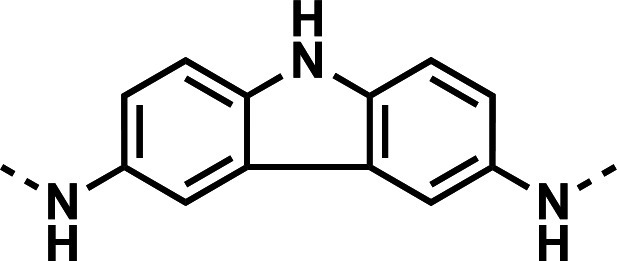	6 ± 2	6 ± 6	24.5	3.6
**15**	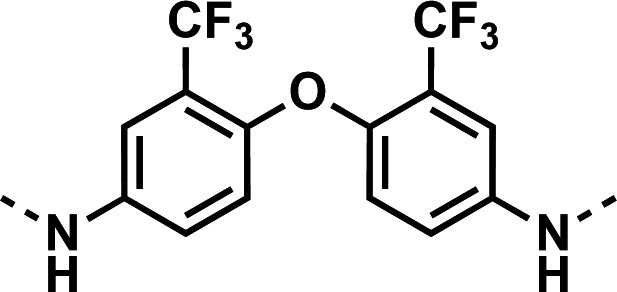	45 ± 16	38 ± 8	≥200	≥3.6
**17**	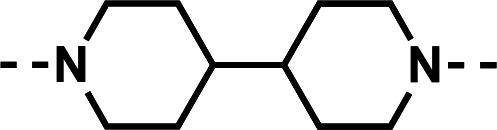	16 ± 4	28 ± 4	58.0	>3.5
**18**	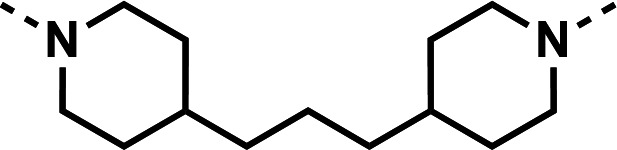	170 ± 60	140 ± 20	≥200	>3.1

^*a*^ Antiplasmodial activity (IC_50_) values and standard deviations (SD) were derived from the average of at least three independent experiments, each performed in quadruplicate. Cytotoxicity (CC_50_) assay was performed using human hepatoma-derived HepG2 cells across an initial concentration of 200 µM to 10 nM in triplicate. Log of selectivity index (pSI) calculated using equation 1; pSI classification: (<1) = potentially cytotoxic/nonselective, (1–2) = low selectivity, (2–3) = moderate selectivity, (3–4) = high selectivity, >4 = very high selectivity. Details of assays performed are found in the Materials and Methods section.

^*b*^ Markush structure of compounds in the table (top), followed by the activity of each compound against a drug-sensitive strain of *Pf* (*Pf*D6), a multidrug-resistant strain of *Pf* (*Pf*Dd2), and cytotoxicity against HepG2 mammalian cells.

^*c*^ Cytotoxicity of **CQ** and **PPQ** reported from published sources (20, 21).

**TABLE 2:** Structure-activity relationship profiling of bisquinolines vs drug-sensitive (D6) and multidrug-resistant (Dd2) strains of *Pf*: impact of quinoline substitution pattern on antiplasmodial activity and cytotoxicity^*a*^^,^^*b*^

				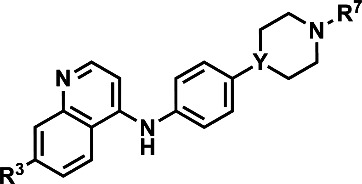			
Compound	R^3^	Y	R^7^	IC_50_ vs *Pf*D6 ± SD (nM)	IC_50_ vs *Pf*Dd2 ± SD (nM)	CC_50_ vs HepG2 (µM)	Log selectivity index (pSI)
**23**	Cl	C	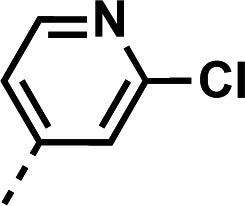	86 ± 23	82 ± 15	≥200	≥3.4
**24**	Cl	C	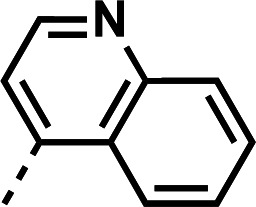	5 ± 2	10.1 ± 0.5	≥200	≥4.6
**25**	Cl	C	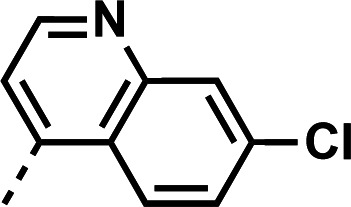	14 ± 3	7.9 ± 1.0	≥200	≥4.2
**26**	Cl	N	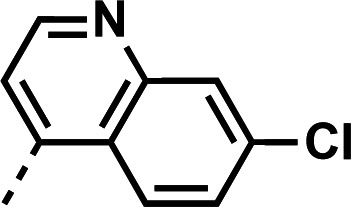	8.1 ± 1.9	7.4 ± 1.4	≥200	≥4.4
**27**	H	C	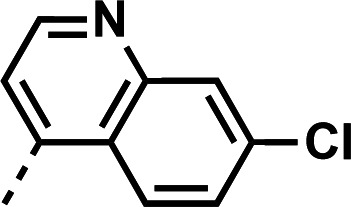	8.5 ± 0.2	10 ± 2	12.2	3.2
**28**	H	C	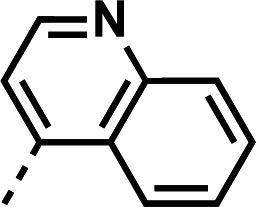	5 ± 3	20 ± 23	11.8	3.4

^*a*^ Antiplasmodial activity (IC_50_) values and standard deviations (SD) were derived from the average of at least three independent experiments, each performed in quadruplicate. Cytotoxicity (CC_50_) assay was performed using human hepatoma-derived HepG2 cells across an initial concentration of 200 µM to 10 nM in triplicate. Log of selectivity index (pSI) calculated using equation 1; pSI classification: (<1) = potentially cytotoxic/nonselective, (1–2) = low selectivity, (2–3) = moderate selectivity, (3–4) = high selectivity, >4 = very high selectivity. Details of assays performed are found in the Materials and Methods section.

^*b*^ Markush structure of compounds in the table (top), and the activity of each compound against a drug-sensitive strain of *Pf* (*Pf*D6), a multidrug-resistant strain of *Pf* (*Pf*Dd2), and cytotoxicity against HepG2 mammalian cells.

### Metabolic stability and *in vivo* efficacy of selected bisquinolines

Compounds from Tables 1 and 2 that exhibited both excellent therapeutic potential (*Pf*D6 and *Pf*Dd2 IC_50_ < 20 nM) and demonstrated a high selectivity index value (pSI > 3) were chosen for evaluation for *in vivo* efficacy experiments. Prior to *in vivo* experiments, the intrinsic metabolic stability (t_1/2_) of each selected compound was measured in pooled murine liver microsomes, with values shown in Tables 3 and 4. A predicted ratio of compound that would remain in circulation after first-pass metabolism, predicted hepatic extraction ratio (E_H_), was calculated from each t_1/2_ (22). Both t_1/2_ and E_H_ were used to aid in the down-selection and interpretation of *in vivo* experiments moving forward. The results of these experiments on compounds presented in Table 1 are reported in Table 3, and the results of compounds from Table 2 are found within Table 4.

**TABLE 3:** Intrinsic metabolic stability and *in vivo* efficacy against *P. yoelii* murine malaria model of bisquinolines from Table 1^*a*^^,^^*b*^

Compound	IC_50_ vs *Pf*D6 ± SD (nM)	*Py* suppression at 5 mg/kg/day (%)	t_1/2_ (min)	E_H_
**8**	14.6 ± 1.3	0	398	0.13
**10**	11 ± 4	100	58.5	0.51
**11**	91 ± 6	48	247	0.20
**12**	10.4 ±1.9	63	55.8	0.52
**13**	19 ± 10	–	16.4	0.79
**14**	6 ± 2	78	3.06	0.95
**17**	16 ± 4	0	23.4	0.72

^*a*^ *In vitro* half-life (t_1/2_) determined from HPLC UV traces of drug after incubation with pooled murine liver microsomes. *In vivo* efficacy (% *Py* suppression) was the percent suppression of parasitemia of a treated group compared to the control group. Predicted hepatic extraction ratio (E*_H_*) calculated from t_1/2_ using equation 2, E*_H_* classification range: (<0.3) = low, (0.3–0.7) = intermediate, (0.7–0.95) = high, (>0.95) = very high. Full procedure for microsomal stability and *in vivo* assays can be found in the Materials and Methods section. (–) = not performed. All animal studies used group sizes of *n* = 4 per dose regimen.

^*b*^ Activity of selected compounds from Table 1 against drug-sensitive *Pf* (IC_50_ vs *Pf*D6) and their efficacy in a murine malaria infection challenge at 5 mg/kg/day (*py* suppression). Compounds were evaluated for intrinsic metabolic stability (t_1/2_) in pooled liver murine microsomes and their predicted *in vivo* hepatic extraction ratio calculated (E_H_).

**TABLE 4:** Intrinsic metabolic stability and *in vivo* efficacy against *P. yoelii* murine malaria model of bisquinolines from Table 2^*a*^^,^^*b*^

Compound	IC_50_ vs *Pf* D6 ± SD (nM)	*Py* suppression at 5 mg/kg/day (%)	ED_50_ vs P*y* (mg/kg/day)	ED_90_ vs P*y* (mg/kg/day)	NRD (mg/kg/day)	t_1/2_ (min)	E*_H_*
**24**	5 ± 2	100	–	–	–	42.3	0.59
**25**	14 ± 3	100	0.32	0.51	10	121	0.33
**26**	8.1 ± 1.9	100	0.28	0.98	ND (10)	34.4	0.64
**27**	8.5 ± 0.2	–	–	–	–	38.6	0.61
**28**	5 ± 3	85	–	–	–	14.3	0.81

^*a*^ *In vitro* half-life (t_1/2_) determined from HPLC UV traces of drug after incubation with pooled murine liver microsomes. *In vivo* efficacy (% *Py* suppression) was the percent suppression of parasitemia of a treated group compared to the control group. Predicted hepatic extraction ratio (E*_H_*) calculated from t_1/2_ using equation 2, E*_H_* classification range: (<0.3) = low, (0.3–0.7) = intermediate, (0.7–0.95) = high, (>0.95) = very high. Full procedure for microsomal stability and *in vivo* assays can be found in the Materials and Methods section. (–) = not performed. ND() = not detected at corresponding dosage. All animal studies used group sizes of *n* = 4 per dose regimen.

^*b*^ Activity of selected compounds from Table 2 against drug-sensitive *Pf* (IC_50_ vs *Pf*D6) and their efficacy in a murine malaria infection challenge at 5 mg/kg/day (*py* suppression). Compounds were evaluated for intrinsic metabolic stability in pooled liver murine microsomes (t_1/2_) and their predicted *in vivo* hepatic extraction ratio calculated (E_H_). Downselected compounds were reexamined for degree of *in vivo* efficacy (ED_50_ and ED_90_) and protection from recrudescence (NRD).

Notice that **8** and **11** possess excellent intrinsic metabolic stability in murine microsomes (t_1/2_ = 399 min and 247 min, respectively). We had hypothesized that reducing rotational freedom of the diphenylamine linker may enhance metabolic stability, but compared to **11**, the carbazole linker in **14** introduced a metabolic liability, substantially reducing *in vitro* half-life to 3.06 min. From the series presented in Table 2, compound **25** possesses enhanced intrinsic metabolic stability (t_1/2_ = 121 min) compared to the corresponding piperazine analog **26** (t_1/2_ = 34.38 min). A clear trend of metabolic stability emerged among the piperidine-containing compounds **24**, **25**, **27**, and **28**. If the presence of two 7-position chlorine atoms (**25**) is reduced to include only one chlorine atom on either quinoline (**24** and **27**), the *in vitro* metabolic stability is substantially reduced, t_1/2_ = 42.3 min and 38.6 min, respectively. This is further illustrated by the complete absence of 7-position chlorine atoms in **28** and its *in vitro* half-life of 14.33 min, near an order of magnitude shorter than **25**.

Select compounds were then evaluated for *in vivo* efficacy of parasite clearance in a murine malaria challenge model at 5 mg/kg/day in PEG-400 using a modified Peter’s 4-Day test (14). Regardless of the similar *in vitro* activity across compounds in Table 3, only **10** was able to completely clear the infection by Day 5 of the challenge at this dose. This was an unexpected result considering that **8** and **11** exhibit the two longest *in vitro* half-lives reported in Tables 3 and Table 4. Despite the increased metabolic liability of **14** compared to its bioisostere **11**, it was more efficacious *in vivo* (*Py* suppression of: **14** = 78%, **11** = 48%). Of the compounds represented in Table 4, **24–26** exhibit excellent *in vivo* efficacy and completely cleared *P. yoelii* infection by Day 5 of the study, despite their relative differences in *in vitro* metabolic stability. Compound **28** exhibited respectable efficacy *in vivo* (*Py* suppression = 85%), but not to the same degree as **24–26**. Therefore, **25** was selected for further *in vivo* studies to more fully assess its efficacy in this malaria infection model.

Excitingly, **25** demonstrated a high level of *in vivo* efficacy, with a 90% reduction in parasitemia compared to the control (PEG-400 only), at 0.51 mg/kg/day. Perhaps an even more exciting result was that **25** completely cleared the infection and protected the animals against parasite recrudescence (NRD) at 10 mg/kg/day. Given the superior *in vivo* performance of **25**, we decided to further assess the *in vivo* efficacy of its bioisostere **26**. The compound exhibited similar efficacy (ED_50_ = 0.32 mg/kg/day, ED_90_ = 0.51 mg/kg/day) to **25**; however, a non-recrudescence dose (NRD) was not achieved across the entire tested dose range. With these results in hand, we moved to further evaluate the clinical potential of **25** as the frontrunner compound.

### Single-dose PKs of compound 25

**Fig 7:**
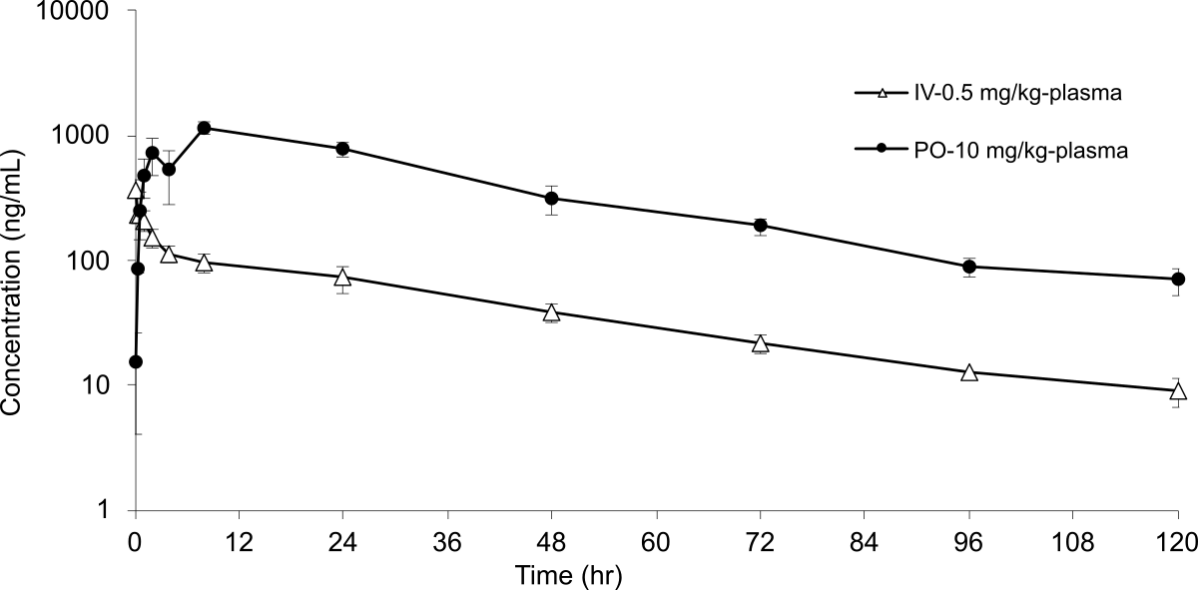
Mean plasma concentration-time profiles of 25 after IV dose at 0.5 mg/kg and PO dose at 10 mg/kg in male CD1 mice (*n* = 3). Compound 25 was prepared in PEG-400 to yield clear solutions at 0.1 mg/mL for IV via tail vein and 1.0 mg/mL for PO dosing via oral gavage. No abnormal clinical symptoms were observed during the entire in-life study.

To evaluate the *in vivo* performance of **25**, a dual-arm (oral and IV) PK experiment was performed. Plasma concentration versus time of **25** following single-dose oral gavage (PO) of 10 mg/kg and intravenous injection (IV) of 0.5 mg/kg administration of **25** in mice is shown in Fig. 7, and the resulting PK parameters are summarized below in Table 5. It is noteworthy that **25** appears to be moderately well absorbed, with an absolute oral bioavailability of 43% (F) and a maximum blood-stream concentration reached in 8 h (*T*_max_), achieving a maximum concentration of 1,162 ng/mL (*C*_max_). The bloodstream half-life of 25 given through IV administration compared to PO administration agreed well across the two arms of the experiment, T_1/2_ = 30.9 h and 27.2 h, respectively.

**TABLE 5:** PK properties of 25 in male CD1 mice (*n* = 3) after PO dose of 10 mg/kg and IV dose of 0.5 mg/kg^*a*^

Administration	Parameter	Calculated value	Administration	Parameter	Calculated value
0.5 mg/kg - IV	CL	1.49 mL/min/kg	10.0 mg/kg - PO	*T*_max_	8.0 h
	V_ss_	3.76 L/kg		*C*_max_	1,162 ng/mL
	T_1/2_	30.9 h		T_1/2_	27.2 h
	AUC_INF_	5,580 h × ng/mL		AUC_INF_	48,126 h × ng/mL
	MRT_INF_	41.9 h		MRT_INF_	39.5 h
				F	43%

^*a*^ PK parameters were estimated by non-compartmental model using WinNonlin version 8.2.

### Structure-activity profiling of bisquinolines

Initial designs of bisquinolines focused on combinations of key structural features, namely the quinoline core, aniline, and piperazine moiety. First, we sought to use the simplest bis-aniline linker—benzidine, passing over *p*-phenylenediamine as a starting point due to the work described by Ismail et al. (23), to create compound **8** (23). We next sought to disrupt coplanarity in the aromatic rings found in the linker of **8**, by adding opposing fluorine atoms, seen in **9**. This change, however, had a great cost to activity against both strains of *Pf* (*Pf*D6 IC_50_ ≥ 250 nM, *Pf*Dd2 IC_50_ = 111 nM) compared to the parent compound **8**, so this design strategy was abandoned. We next introduced complexity and flexibility by introducing a carbon, nitrogen, or oxygen atom between the two aromatic rings resulting in methylene (**10**), amine (**11**), and ether (**12**) bi-aryls. Notice that all three compounds exhibit similar antiplasmodial activity, and the only difference of note is that **11** exhibited cytotoxicity against HepG2 cells (CC_50_ = 100 µM). To further increase the chemical diversity of each of these three compounds from one another, we introduced a dimethyl to the methylene linker (**13**), changed the diphenylamine linker to a carbazole (**14**), and introduced trifluoromethyl groups to the biaryl ether (**15**). These changes to the chemical structure showed a slight reduction in the antiplasmodial activity across both strains of **13** (*Pf*D6 IC_50_ = 19 nM, *Pf*Dd2 IC_50_ = 15 nM) and in **15** (*Pf*D6 IC_50_ = 45 nM, *Pf*Dd2 IC_50_ = 38 nM) when compared to the parent compounds. And both **13** and **14** saw an increase in cytotoxicity, CC_50_ = 96 µM and 25 µM, respectively, compared to the corresponding parent compound. We incorporated the piperidine moiety, as outlined in Fig. 2B, in **17** as a bridged bi-piperidine, and in **18** as a piperidine-based analog of PPQ by including a *n*-propyl alkane between the two rings. Compound **17** showed excellent antiplasmodial activity, *Pf*D6 IC_50_ = 17 nM, *Pf*Dd2 IC_50_ = 28 nM; however, **18**, which we expected to be similarly active to PPQ, exhibited over an order of magnitude reduction in potency against drug-sensitive (*Pf*D6 IC_50_ = 170 nM) and a substantial reduction in potency against multidrug-resistant *Pf* (*Pf*Dd2 IC_50_ = 140 nM), when compared to **17**.

The next series of compounds contained all three structural components—the quinoline cores, the aniline, and the piperidine. Compound **23** was prepared to probe whether the piperidinyl quinoline ring feature is required for antiplasmodial activity or whether an alternative nitrogen-containing aromatic constituent could maintain potency. Although **23** retains activity (*Pf*D6 IC_50_ = 86 ± 23 nM, *Pf*Dd2 IC_50_ = 82 ± 15 nM), it was notably less potent than most analogs in Table 1. Compounds **24–28** were made to evaluate the role of the 7-position chlorine found in the quinoline rings, but we found that the chlorine can be removed from either quinoline ring or both without a noticeable change in potency (IC_50_s vs *Pf*D6 = 5–14 nM; *Pf*Dd2 = 7–20 nM). However, the 4-anilinoquinoline chlorine is clearly important for maintaining low cytotoxicity, as bioisosteres **27** and **28** (CC_50_ ≈ 12 µM) were markedly more cytotoxic than **24** (CC_50_ > 200 µM), despite the latter being a positional isomer of **27**.

Despite some compounds exhibiting measurable cytotoxicity, all but compound **9** demonstrate strong selectivity for *Pf* over the reference mammalian cell line, with selectivity indices (pSI) ≥ 3, many demonstrating pSI ≥ 4—highlighting their potential as promising antimalarial candidates. And while antiplasmodial potency was broadly conserved across the entire series, as 11 of 16 compounds exhibit *Pf*D6 and *Pf*Dd2 IC_50_s ≤ 20 nM, differences in metabolic stability and parasite suppression revealed some key structure-property relationships.

### DMPK-efficacy profiling of bisquinolines

Table 3 indicates no clear relationships between *in vitro* potency and *in vivo* efficacy of the compounds from Table 1. Despite their high potency (*Pf*D6 and *Pf*Dd2 IC_50_s ≤ 20 nM) and low predicted hepatic extraction ratio (E_H_ < 0.3), compounds **8** and **11** were less effective at suppressing parasitemia in the murine malaria model than other compounds with similar potency but higher predicted hepatic extraction. For example, compound **10** completely suppressed infection notwithstanding a predicted intermediate level of hepatic extraction during first-pass metabolism (E_H_ = 0.51). Similarly, compound **14** suppressed parasite burden by 78%, despite very high predicted hepatic extraction (E_H_ = 0.95). These results suggest the formation of active metabolites of **10** and **14** with enhanced *in vivo* efficacy, or insufficient absorption and distribution of compounds **8** and **11**, which reduces their ability to suppress parasitemia burden.

A similar observation can be made for **12**, in that the degree of *in vitro* potency and predicted hepatic extraction ratio (E_H_ = 0.52) should lead to robust *in vivo* efficacy; however, parasitemia was suppressed by only 63% on day 5. Again, this could be attributed to partial or insufficient absorption and distribution, which in turn reduces efficacy.

Compounds reported in Table 4 do not suffer from the same disparity between potency, metabolic stability, and *in vivo* efficacy as seen in the compounds listed in Table 3. Clearly, **24**, **25**, **26**, and **28** demonstrate impressive *in vivo* efficacy, which matches in kind with their reduced metabolic stability. In essence, **28** is as potent *in vitro* as **24**, but the decreased metabolic stability of **28** predicts a high level of hepatic extraction (E_H_ = 0.81), which is reflected by a diminished *in vivo* efficacy compared to **24**, *Py* suppression = 85% and 100%, respectively. Furthermore, we can observe this agreement when **25** and **26** are compared. Compound **26** is marginally more potent than **25** *in vitro,* which likely contributes to a lower ED_50_ than **25**. However, one possible explanation for why the ED_90_ of **25** is lower than the ED_90_ of **26** is that a higher fraction of **25** is predicted to remain in circulation after first-pass metabolism (E_H_ = 0.33 and 0.64, respectively), allowing an effective therapeutic concentration to be reached and maintained for longer duration. This reasoning could ultimately explain the recrudescence of *P. yoelii* observed when mice were treated with **26** at a similar dose to **25**, whereas **25** cured the infection and provided full protection against parasite recrudescence (NRD = 10 mg/kg/day).

### Concluding remarks and future directions

We have previously used historical antiparasitic drugs as potential starting points for new chemical leads for the development of novel antimalarials, e.g. using endochin to develop endochin-like quinolones (24–27). Our first foray into 4-aminoquinolines led to the development of the pharmachins, a series of sontochin-based analogs that ultimately led to highly active and efficacious antimalarials (28). Our second campaign into this chemical space originated from a desire to improve the metabolic instability and cytotoxicity observed in the use of AQ. This work resulted in the amodiachin series, which produced a metabolically stable (T_1/2_ = 84 h) and efficacious antimalarial, ADC-028 (1). Our initial rationale for the work described here set out to expand the SAR around that scaffold by incorporating key structural elements of 1 into a bisquinoline arrangement similar to PPQ.

We hoped to find compounds with enhanced antiplasmodial activity, safety, metabolic stability, and *in vivo* efficacy in that chemical space. To this end, compound **25** exhibits enhanced antiplasmodial activity against both drug-sensitive (D6) and multidrug-resistant (Dd2) strains of *Pf* compared to compound **1**. The two compounds have similar safety profiles against HepG2, but **25** exhibits an increased intrinsic metabolic stability, t_1/2_ = 121 min. The efficacy of **25** *in vivo* is far superior to its parent compound, with a sevenfold increase in potency, ED_90_ = 0.51 mg/kg/day and 2.5 mg/kg/day, respectively, in addition to protecting animals from recrudescence at two-thirds of the dose, NRD of **1** = 16 mg/kg/day and a NRD of **25** = 10 mg/kg/day. PK analysis of **25** found that the half-life of an oral dose was 27.2 h in the mouse model, effectively a third of the half-life of **1** using the same route of administration, but still incredibly stable and long-lasting in this model. Unfortunately, the oral bioavailability (F = 43%) of **25** is nearly half of compound **1** (F = 76%). Future work in this chemical space will focus on improving oral bioavailability and *in vivo* metabolic stability of this series in tandem with profiling these and newer analogs against genetically modified and clinical isolates of *Pf* parasites that harbor resistance to additional 4-aminoquinoline antimalarials. In addition, we will perform *ex vivo* β-hematin and *in vitro* hemozoin formation inhibition studies to determine if these compounds share a similar mechanism of action to other 4-aminoquinoline antimalarials. Lastly, we acknowledge the potential of aryl amines to generate reactive metabolites when oxidized, and although our *in vivo* experiments did not indicate phenotypic toxicity markers, we plan on performing metabolite identification studies alongside glutathione-trapping and covalent-binding assays to determine if the aryl amine in **25** undergoes bioactivation to form toxic metabolites. Though **25** does not outperform **1** at every benchmark, it still succeeds at providing an excellent starting point for continued exploration and development within this chemical space.

## MATERIALS AND METHODS

### Chemistry

#### Materials and instruments

All solvents, starting materials, and reagents were acquired from commercial sources, including (but not limited to): Fisher Scientific, TCI Chemicals, Combi Blocks, Enamine, and Sigma-Aldrich. Reaction progress was monitored by TLC, GCMS, or HPLC when permitted. Both reverse-phase and normal-phase flash chromatography were performed using a Biotage Isolera with the following column types: Sfär Silica HC and Sfär C18-Duo. ^1^H NMR spectra were acquired on a Bruker 400 MHz instrument, and chemical shifts are reported relative to TMS (0.0 ppm) or NMR Solvent (CDCl_3_, DMSO-*d*_6_, or CD_3_OD). Final compounds are reported as >95% pure, as determined via NMR, since many compounds produced did not exhibit necessary solubility in HPLC mobile-phase solvents. High-resolution accurate mass mass spectrometry (HRAM/MS) using electrospray ionization was performed by the Oregon Health & Science University’s Bioanalytical Shared Resource/Pharmacokinetics Core for additional structure verification.

#### General procedure (i)

A microwave reactor flask equipped with a magnetic stir bar was charged with bis-aniline (1 equiv.), 4-bromo-7-chloroquinoline (3 equiv.), phenol (3.0 equiv.), and DMF (1.0 M). The flask was sealed and heated at 150°C for 2 h under high absorption. Upon completion, the reaction mixture was transferred to an appropriately sized round-bottom flask and concentrated *in vacuo*. The resulting solids were resuspended in dichloromethane. The organic solution was subjected to an aqueous workup in a separatory funnel. The solution was sequentially treated with 2 M sodium hydroxide (to pH > 10), then washed with water, and finally with saturated sodium chloride. The organic layer was separated, dried over magnesium sulfate, and gravity filtered. The filtrate was concentrated *in vacuo* and purified by automated flash chromatography. Fractions containing the desired bis-quinoline were combined and concentrated *in vacuo*.

#### General procedure (ii)

A microwave reactor flask equipped with a magnetic stir bar was charged with aniline (1 equiv.), 4-chloroquinoline (1.2 equiv.), and THF (1.0 M). The flask was sealed and heated at 120°C for 20 min under high absorption. Upon completion, the reaction mixture was filtered and washed with excess THF. The resulting solids were resuspended and triturated with methanol. While suspended in methanol, the purified solids were treated with 2 M sodium hydroxide (to pH > 10) and stirred for 1.5 h. The solution was then concentrated *in vacuo* until only water remained. The resulting suspension was separated by vacuum filtration, and the residue was washed with water. The remaining solids were dried for 24 h to afford the desired product and were used without further purification.

#### General procedure (v)

A microwave reactor flask equipped with a magnetic stir bar was charged with the appropriate free-amine heterocycle-linked anilinoquinoline (1 equiv.), 4-bromo-7-chloroquinoline (2 equiv.), phenol (2.0 equiv.), and DMF (1.0 M). The flask was sealed and heated at 150°C for 2 h under high absorption. Upon completion, the reaction mixture was transferred to an appropriately sized round-bottom flask and concentrated *in vacuo*. The resulting solids were resuspended in dichloromethane. The organic solution was subjected to an aqueous workup in a separatory funnel. The solution was sequentially treated with 2 M sodium hydroxide (to pH > 10), then washed with water, and finally with saturated sodium chloride. The organic layer was separated, dried over magnesium sulfate, and gravity filtered. The filtrate was concentrated *in vacuo* and purified by automated flash chromatography. Fractions containing the desired bis-quinoline were combined and concentrated *in vacuo*.

### Synthetic descriptions of presented compounds

Details of the synthesis of **7**, **21a–c intermediates**, **21a–c**, and **22a–c** are described in the supplementary information.

#### *N*^4^,*N*^4^'-bis(7-chloroquinolin-4-yl)-[1,1'-biphenyl]-4,4'-diamine (8)

The title compound was prepared using General procedure (i) in the following proportions: 0.185 g (1.0 mmol) benzidine, 0.728 g (3.0 mmol, 3.0 equiv.) **7**, 0.189 g (1.95 mmol, 1.9 equiv.) phenol, and 10 mL dimethylformamide (0.1 M). Final product isolated as golden powder. Yield = 91%, 0.464 g (0.91 mmol). ^1^H NMR (DMSO-*d*_6_, 400 MHz) ppm: δ 9.26 (s, 1H), 8.57 (d, 1H, *J =* 5.4 Hz), 8.52 (d, 1H, *J =* 9.1 Hz), 7.98 (d, 1H, *J =* 2.3 Hz), 7.83 (d, 3H, *J =* 8.5 Hz), 7.66 (dd, 2H, *J =* 2.3, 9.0 Hz), 7.53 (d, 3H, *J =* 8.6 Hz), 7.11 (d, 1H, *J =* 5.3 Hz). HRAM/MS (ESI positive): [M+H]^+^ of [C_30_H_20_Cl_2_N_4_] = 507.11377; observed = 507.11375.

#### *N*^4^,*N*^4^'-bis(7-chloroquinolin-4-yl)-2,2'-difluoro-[1,1'-biphenyl]-4,4'-diamine (9)

The title compound was prepared using General procedure (i) in the following proportions: 0.225 g (1.0 mmol) 2,2'-difluoro-[1,1'-biphenyl]-4,4'-diamine, 0.487 g (2.0 mmol, 2.0 equiv.) **7**, 0.177 g (1.88 mmol, 1.8 equiv.) phenol, and 5 mL dimethylformamide (0.2 M). Final product isolated as a yellow solid. Yield = 90%, 0.488 g (0.90 mmol). ^1^H NMR (DMSO-*d*_6_, 400 MHz) δ ppm: 10.96 (br s, 2H), 8.74 (d, 2H, *J =* 9.1 Hz), 8.67 (d, 2H, *J =* 6.9 Hz), 8.09 (d, 2H, *J =* 2.0 Hz), 7.97 (dd, 2H, *J =* 2.0, 9.1 Hz), 7.74 (td, 2H, *J =* 4.4, 8.4 Hz), 7.6–7.6 (m, 2H), 7.52 (dd, 2H, *J =* 2.0, 8.3 Hz), 7.15 (d, 2H, *J =* 6.9 Hz). HRAM/MS (ESI positive): [M+H]^+^ of [C_30_H_18_Cl_2_F_2_N_4_] = 543.09493; observed = 543.09516.

#### *N*,*N*'-(methylenebis(4,1-phenylene))bis(7-chloroquinolin-4-amine) (10)

The title compound was prepared using General procedure (i) in the following proportions: 0.199 g (1.0 mmol) 4,4'-methylenedianiline, 0.537 g (2.2 mmol, 2.2 equiv.) **7**, 0.281 g (3.00 mmol, 3 equiv.) phenol, and 3 mL dimethylformamide (0.33 M). Final product isolated as yellow-tan powder. Yield = 91%, 0.475 g (0.91 mmol). ^1^H NMR (DMSO-*d*_6_, 400 MHz) δ ppm: 9.95 (br s, 2H), 8.56 (d, 2H, *J =* 9.1 Hz), 8.48 (d, 2H, *J =* 6.1 Hz), 7.95 (d, 2H, *J =* 2.1 Hz), 7.74 (dd, 2H, *J =* 9.0, 1.9 Hz), 7.40 (q, 8H, *J* = 8.9 Hz), 6.85 (d, 2H, *J =* 6.3 Hz), 4.06 (s, 2H). HRAM/MS (ESI positive): [M+H]^+^ of [C_31_H_22_Cl_2_N_4_] = 521.12942; observed = 521.12947.

#### *N*^1^-(7-chloroquinolin-4-yl)-*N*^4^-(4-((7-chloroquinolin-4-yl)amino)phenyl)benzene-1,4-diamine (11)

The title compound was prepared using General procedure (i) in the following proportions: 0.399 g (2.0 mmol) *N*1-(4-aminophenyl)benzene-1,4-diamine, 0.638 g (6.8 mmol, 3.4 equiv.) **7**, 1.170 g (4.8 mmol, 2.4 equiv.) phenol, and 10 mL dimethylformamide (0.2 M). Final product isolated as a tan-brown solid. Yield = 46%, 0.486 g (0.93 mmol). ^1^H NMR (DMSO-*d*_6_, 400 MHz) δ ppm: 8.98 (s, 2H), 8.4–8.5 (m, 4H), 8.30 (s, 1H), 7.87 (d, 2H, *J =* 2.1 Hz), 7.55 (dd, 2H, *J =* 2.3, 9.0 Hz), 7.2–7.3 (m, 4H), 7.2–7.2 (m, 4H), 6.70 (d, 2H, *J =* 5.4 Hz). HRAM/MS (ESI positive): [M+H]^+^ of [C_30_H_21_Cl_2_N_5_] = 522.12467; observed = 522.12476.

#### *N*,*N*'-(oxybis(4,1-phenylene))bis(7-chloroquinolin-4-amine) (12)

The title compound was prepared using General procedure (i) in the following proportions: 0.200 g (1.0 mmol) 4,4'-oxydianiline, 0.5148 g (2.1 mmol, 2.1 equiv.) **7**, 0.308 g (3.3 mmol, 3.3 equiv.) phenol, and 3 mL dimethylformamide (0.33 M). Final product isolated as white powder. Yield = 91%, 0.477 g (0.91 mmol). ^1^H NMR (DMSO-*d*_6_, 400 MHz) δ ppm: 9.09 (s, 1H), 8.4–8.5 (m, 2H), 7.89 (d, 1H, *J =* 2.3 Hz), 7.58 (dd, 1H, *J =* 2.2, 9.1 Hz), 7.4–7.4 (m, 2H), 7.1–7.2 (m, 2H), 6.84 (d, 1H, *J =* 5.4 Hz). HRAM/MS (ESI negative): [M-H]^-^ of [C_30_H_20_Cl_2_N_4_O] = 521.09304; observed = 521.09434.

#### *N*,*N*'-(propane-2,2-diylbis(4,1-phenylene))bis(7-chloroquinolin-4-amine) (13)

The title compound was prepared using General procedure (i) in the following proportions: 0.229 g (1.0 mmol) 4,4'-(propane-2,2-diyl)dianiline, 0.539 g (2.2 mmol, 2.2 equiv.) **7**, 0.291 g (3.1 mmol, 3 equiv.) phenol, and 4 mL dimethylformamide (0.25 M). Final product isolated as a gold-hued solid. Yield = 41%, 0.225 g (0.41 mmol). ^1^H NMR (DMSO-*d*_6_, 400 MHz) δ ppm: 9.05 (s, 2H), 8.4-8.5 (m, 4H), 7.89 (d, 2H, *J =* 2.1 Hz), 7.57 (dd, 2H, *J =* 2.3, 9.0 Hz), 7.3–7.4 (m, 8H), 6.91 (d, 2H, *J =* 5.4 Hz), 1.71 (s, 6H). HRAM/MS (ESI negative): [M-H]^-^ of [C_33_H_26_Cl_2_N_4_] = 547.14507; observed = 547.14650.

#### *N*^3^,*N*^6^-bis(7-chloroquinolin-4-yl)−9H-carbazole-3,6-diamine (14)

The title compound was prepared using General procedure (i) in the following proportions: 0.205 g (1.0 mmol) 9*H*-carbazole-3,6-diamine, 0.538 g (2.2 mmol, 2.2 equiv.) **7**, 0.288 g (3.0 mmol, 3 equiv.) phenol, and 3 mL dimethylformamide (0.33 M). Final product isolated as a brown solid. Yield = 37%, 0.201 g (0.39 mmol). ^1^H-NMR (DMSO-*d*_6_, 400 MHz) δ ppm: 11.44 (s, 1H), 9.17 (s, 2H), 8.50 (d, 2H, *J =* 9.1 Hz), 8.36 (d, 2H, *J =* 5.4 Hz), 8.13 (d, 2H, *J =* 1.9 Hz), 7.86 (d, 2H, *J =* 2.3 Hz), 7.60 (d, 2H, *J =* 8.5 Hz), 7.55–7.58 (m, 2H), 7.36–7.44 (m, 2H), 6.64 (d, 2H, *J =* 5.4 Hz). HRAM/MS (ESI positive): [M+H]^+^ of [C_30_H_19_Cl_2_N_5_] = 520.10907; observed = 520.10907.

#### *N*,*N*'-(oxybis(3-(trifluoromethyl)−4,1-phenylene))bis(7-chloroquinolin-4-amine) (15)

The title compound was prepared using General procedure (i) in the following proportions: 0.338 g (1.0 mmol) 4,4'-oxybis(3-(trifluoromethyl)aniline), 0.536 g (2.2 mmol, 2.2 equiv.) **7**, 0.291 g (3.00 mmol, 3 equiv.) phenol, and 3 mL dimethylformamide (0.33 M). Final product isolated as a white solid. Yield = 48%, 0.319 g (0.48 mmol). ^1^H-NMR (DMSO-*d*_6_, 400 MHz) δ ppm: 9.30 (s, 2H), 8.53 (d, 2H, *J =* 5.3 Hz), 8.40 (d, 2H, *J =* 9.1 Hz), 7.93 (d, 2H, *J* = 2.0 Hz), 7.76 (d, 2H, *J =* 2.3 Hz), 7.7–7.7 (m, 2H), 7.63 (dd, 2H, *J =* 9.0, 2.1 Hz), 7.23 (d, 2H, *J =* 8.8 Hz), 7.01 (b d, 2H *J =* 5.0 Hz). HRAM/MS (ESI positive): [M+H]^+^ of [C_32_H_18_Cl_2_F_6_N_4_O] = 659.08375; observed = 659.08375.

#### 1,1'-bis(7-chloroquinolin-4-yl)−4,4'-bipiperidine (17)

The title compound was prepared using General procedure (i) in the following proportions: 0.563 g (2.1 mmol) tert-butyl [4,4'-bipiperidine]−1-carboxylate, 1.663 g (6.7 mmol, 3.2 equiv.) **7**, 0.371 g (3.9 mmol, 3 equiv.) phenol, and 3 mL dimethylformamide (0.33 M). Final product isolated as tan powder. Yield = 42%, 0.432 g (0.88 mmol). ^1^H NMR (DMSO-*d*_6_, 400 MHz) δ ppm: 8.69 (d, 2H, *J =* 5.0 Hz), 8.02 (d, 2H, *J =* 9.0 Hz), 7.97 (d, 2H, *J =* 2.0 Hz), 7.57 (dd, 2H, *J =* 1.9, 8.9 Hz), 7.02 (d, 2H, *J =* 5.1 Hz), 3.63 (br d, 5H, *J =* 12.1 Hz), 2.85 (br t, 5H, *J =* 11.6 Hz), 1.94 (br d, 4H, *J =* 12.1 Hz), 1.5–1.7 (m, 4H), 1.47 (br d, 2H, *J =* 8.6 Hz). HRAM/MS (ESI): [M+H]^+^ of [C_28_H_28_Cl_2_N_4_] = 491.17637; observed = 491.17673.

#### 1,3-bis(1-(7-chloroquinolin-4-yl)piperidin-4-yl)propane (18)

The title compound was prepared using General procedure (i) in the following proportions: 0.176 g (0.83 mmol) of 1,3-di(piperidin-4-yl)propane, 0.628 g (2.6 mmol, 3 equiv.) of **7**, 0.191 g (2.0 mmol, 2.4 equiv.) of phenol, and 3 mL dimethylformamide (0.28 M). Final product isolated as a clear solid. Yield = 26%, 0.115 g (0.21 mmol). ^1^H NMR (DMSO-*d*_6_, 400 MHz) δ ppm: 8.68 (d, 2H, *J =* 5.0 Hz), 7.9–8.0 (m, 4H), 7.55 (dd, 2H, *J =* 2.3, 9.0 Hz), 6.98 (d, 2H, *J =* 5.0 Hz), 3.53 (br d, 4H, *J =* 12.1 Hz), 2.81 (br t, 4H, *J =* 11.3 Hz), 1.85 (br d, 4H, *J =* 9.5 Hz), 1.3–1.6 (m, 12H)**.** HRAM/MS (ESI positive): [M+H]^+^ of [C_31_H_34_Cl_2_N_4_] = 533.22332; observed = 533.22325.

#### 7-chloro-*N*-(4-(1-(2-chloropyridin-4-yl)piperidin-4-yl)phenyl)quinolin-4-amine (23)

The title compound was prepared using General procedure (iv) in the following proportions: 0.340 g (1.0 mmol) of **21a**, 0.218 g (2.2 mmol, 2.2 equiv.) 2-chloro-4-fluoropyridine, and 0.25 mL (1.4 mmol, 1.4 equiv.) *N*,*N*-diisopropylethylamine, and 2 mL (0.5 M) N-methyl-2-pyrillidone. Final product isolated as white powder. Yield = 30%, 0.130 g (0.30 mmol). ^1^H NMR (DMSO-*d*_6_, 400 MHz) δ ppm: 9.04 (s, 1H), 8.4–8.5 (m, 2H), 7.94 (d, 1H, *J =* 5.7 Hz), 7.88 (d, 1H, *J =* 2.3 Hz), 7.56 (dd, 1H, *J =* 2.3, 9.0 Hz), 7.3–7.3 (m, 4H), 6.9–6.9 (m, 3H), 4.11 (br d, 2H, *J =* 13.4 Hz), 2.9–3.0 (m, 2H), 2.85 (tt, 1H, *J =* 3.4, 12.0 Hz), 1.88 (br d, 2H, *J =* 11.9 Hz), 1.64 (dq, 2H, *J =* 3.6, 12.5 Hz). HRAM/MS (ESI positive): [M+H]^+^ of [C_25_H_22_Cl_2_N_4_] = 449.12942; observed = 449.12952.

#### 7-chloro-*N*-(4-(1-(quinolin-4-yl)piperidin-4-yl)phenyl)quinolin-4-amine (24)

The title compound was prepared using General procedure (v) in the following proportions: 0.334 g (0.99 mmol) of **21a**, 0.417 g (2.00 mmol, 2.0 equiv.) of **19**, 0.201 g (2.1 mmol, 2.1 equiv.) phenol, and 3 mL dimethylformamide (0.33 M). Final product isolated as white powder. Yield = 53%, 0.246 g (0.53 mmol). ^1^H NMR (DMSO-*d*_6_, 400 MHz) δ ppm: 9.07 (s, 1H), 8.70 (d, 1H, *J =* 5.0 Hz), 8.4–8.5 (m, 2H), 8.09 (d, 1H, *J =* 8.5 Hz), 7.96 (dd, 1H, *J =* 0.8, 8.4 Hz), 7.89 (d, 1H, *J =* 2.3 Hz), 7.70 (ddd, 1H, *J =* 1.3, 6.9, 8.3 Hz), 7.5–7.6 (m, 2H), 7.4–7.5 (m, 2H), 7.3–7.4 (m, 2H), 7.03 (d, 1H, *J =* 5.0 Hz), 6.90 (d, 1H, *J =* 5.4 Hz), 3.69 (br d, 2H, *J =* 12.1 Hz), 2.9–3.0 (m, 2H), 2.8–2.9 (m, 1H), 2.0–2.1 (m, 4H). HRAM/MS (ESI positive): [M+H]^+^ of [C_29_H_25_ClN_4_] = 465.18405; observed = 465.18392.

#### 7-chloro-*N*-(4-(1-(7-chloroquinolin-4-yl)piperidin-4-yl)phenyl)quinolin-4-amine (25)

The title compound was prepared using General procedure (v) in the following proportions: 0.335 g (1.0 mmol) of **21a**, 0.484 g (2.0 mmol, 2 equiv.) of **7**, 0.207 g (2.2 mmol, 2.2 equiv.) phenol, and 5 mL dimethylformamide (0.33 M). Final product isolated as a tan powder. Yield = 86%, 0.432 g (0.86 mmol). ^1^H NMR (CHLOROFORM-*d*, 400 MHz) δ ppm: 8.74 (d, 1H, *J =* 5.0 Hz), 8.57 (d, 1H, *J =* 5.3 Hz), 8.05 (t, 2H, *J =* 2.3 Hz), 8.00 (d, 1H, *J =* 9.0 Hz), 7.87 (d, 1H, *J =* 9.0 Hz), 7.46 (t, 2H, *J =* 9.2 Hz), 7.4–7.4 (m, 2H), 7.3–7.3 (m, 2H), 6.96 (d, 1H, *J =* 5.3 Hz), 6.89 (d, 1H, *J =* 5.0 Hz), 6.61 (br s, 1H), 3.74 (br d, 2H, *J =* 12.1 Hz), 2.9–3.1 (m, 2H), 2.7–2.9 (m, 1H), 2.0–2.2 (m, 4H). HRAM/MS (ESI negative): [M-H]^-^ of [C_29_H_24_Cl_2_N_4_] = 497.12942; observed = 497.13077.

#### 7-chloro-*N*-(4-(4-(7-chloroquinolin-4-yl)piperazin-1-yl)phenyl)quinolin-4-amine (26)

The title compound was prepared using General procedure (v) in the following proportions: 0.058 g (0.17 mmol) of **21b**, 0.083 g (0.34 mmol, 2 equiv.) **7**, 0.032 g (0.34 mmol, 2 equiv.) phenol, and 2 mL dimethylformamide (0.1 M). Final product isolated as a yellow solid. Yield = 31%, 0.026 g (0.052 mmol). ^1^H NMR (CHLOROFORM-*d*, 400 MHz) δ ppm: 8.77 (d, 1H, *J =* 4.9 Hz), 8.52 (d, 1H, *J =* 5.3 Hz), 8.08 (d, 1H, *J =* 2.1 Hz), 8.02 (dd, 2H, *J =* 3.4, 5.6 Hz), 7.85 (d, 1H, *J =* 9.0 Hz), 7.46 (ddd, 2H, *J =* 2.3, 7.0, 9.0 Hz), 7.3–7.3 (m, 1H), 7.2–7.3 (m, 1H), 7.1–7.1 (m, 2H), 6.92 (d, 1H, *J =* 5.1 Hz), 6.75 (d, 1H, *J =* 5.4 Hz), 6.54 (s, 1H), 3.5–3.5 (m, 4H), 3.4–3.4 (m, 4H). HRAM/MS (ESI positive): [M+H]^+^ of [C_28_H_23_Cl_2_N_5_] = 500.14032; observed = 500.14042.

#### *N*-(4-(1-(7-chloroquinolin-4-yl)piperidin-4-yl)phenyl)quinolin-4-amine (27)

The title compound was prepared using General procedure (v) in the following proportions: 0.164 g (0.54 mmol) of **21c**,1 0.207 g (0.85 mmol, 1.6 equiv.) of **7**, 0.102 g (1.1 mmol, 2.0 equiv.) phenol, and 3 mL dimethylformamide (0.2 M). Final product isolated as tan powder. Yield = 22%, 0.055 g (0.12 mmol). ^1^H NMR (DMSO-*d*_6_, 400 MHz) δ ppm: 8.92 (s, 1H), 8.72 (d, 1H, *J =* 5.0 Hz), 8.45 (d, 1H, *J =* 5.4 Hz), 8.39 (d, 1H, *J =* 8.0 Hz), 8.10 (d, 1H, *J =* 9.0 Hz), 7.99 (d, 1H, *J =* 2.1 Hz), 7.87 (d, 1H, *J =* 7.6 Hz), 7.70 (t, 1H, *J =* 7.1 Hz), 7.58 (dd, 1H, *J =* 2.1, 9.0 Hz), 7.53 (t, 1H, *J =* 7.2 Hz), 7.4–7.4 (m, 2H), 7.3–7.4 (m, 2H), 7.06 (d, 1H, *J =* 5.0 Hz), 6.91 (d, 1H, *J =* 5.3 Hz), 3.68 (br d, 2H, *J =* 12.0 Hz), 2.9–3.1 (m, 2H), 2.7–2.9 (m, 1H), 2.0–2.1 (m, 4H). HRAM/MS (ESI positive): [M+H]^+^ of [C_29_H_25_ClN_4_] = 465.18405; observed = 465.18410.

#### *N*-(4-(1-(quinolin-4-yl)piperidin-4-yl)phenyl)quinolin-4-amine (28)

The title compound was prepared using General procedure (v) in the following proportions: 0.203 g (0.67 mmol) of **21c**, 0.196 g (0.94 mmol, 1.4 equiv.) of **19**, 0.119 g (1.3 mmol, 1.9 equiv.) phenol, and 3 mL dimethylformamide (0.22 M). Final product isolated as straw-colored powder. Yield = 61%, 0.177 g (0.41 mmol). ^1^H NMR (DMSO-*d*_6_, 400 MHz) δ ppm: 8.93 (s, 1H), 8.70 (d, 1H, *J =* 4.9 Hz), 8.45 (d, 1H, *J =* 5.3 Hz), 8.40 (d, 1H, *J =* 8.4 Hz), 8.09 (d, 1H, *J =* 8.4 Hz), 7.96 (d, 1H, *J =* 8.4 Hz), 7.88 (d, 1H, *J =* 8.3 Hz), 7.70 (br t, 2H, *J =* 6.3 Hz), 7.55 (tt, 2H, *J =* 7.0, 7.9 Hz), 7.42 (d, 2H, *J =* 8.3 Hz), 7.35 (d, 2H, *J =* 8.3 Hz), 7.04 (d, 1H, *J =* 5.0 Hz), 6.9–7.0 (m, 1H), 3.69 (br d, 2H, *J =* 11.9 Hz), 2.97 (br t, 2H, *J =* 10.4 Hz), 2.7–2.9 (m, 1H), 2.0–2.1 (m, 4H). HRAM/MS (ESI positive): [M+H]^+^ of [C_29_H_26_N_4_] = 431.22302; observed = 431.22282.

### Biology

#### *Pf* strains

The following strains of *Pf* were used in this study and were obtained through BEI Resources, NIAID, NIH: *Pf*, Strain D6 (MRA-285, originally from Sierra Leone, has modest resistance to mefloquine), (46) Strain Dd2 (MRA-150, originated from Indochina; derived from W2-mef and is resistant to CQ, pyrimethamine, and mefloquine).

#### Parasite culture

Frozen stocks of *Pf* parasites were thawed and suspended with human erythrocytes (Lampire Biological Laboratories, Pipersville, PA; <28 days old) at 2% hematocrit. The culture medium used was RPMI-1640 with 25 mM HEPES buffer, 25 mg/L gentamicin sulfate, 45 mg/L hypoxanthine, 10 mM glucose, 2 mM glutamine, and 0.5% Albumax II (complete medium) (20). Cultures were incubated in a standard low-oxygen atmosphere (5% O_2_, 5% CO_2_, 90% N_2_) in a Forma Series II 3110 environmental chamber at 37°C. Parasites were passaged every 3–4 days into a fresh culture flask containing complete medium and erythrocytes.

#### Compound *in vitro* antiplasmodial activity (IC_50_) against *Pf*

Compounds were challenged against D6 and Dd2 strains of *Pf* using the previously described fluorescence-based SYBR Green assay to determine intrinsic antiplasmodial activity (17). Ten millimolar stocks of compounds in DMSO were prepared beforehand and kept in a sealed vial at room temperature in the dark. Fifty microliter aliquots of each compound were dispensed in quadruplicate in a flat-bottomed clear 96-well plate using twofold serial dilutions with the final column left untreated to span a range of 0.25 nM–250 nM. Cultured asynchronous *Pf*-infected erythrocytes in growth medium were added to each well (50 μL; total well volume = 100 µL), so that the final hematocrit = 2% and initial parasitemia = 0.2%. Stocks of 10 mM CQ and 10 mM AQ and uninfected and untreated erythrocytes were used as controls. Prepared plates were incubated in a controlled low-oxygen atmosphere (5% O_2_, 5% CO_2_, 90% N_2_) at 37°C for 72 h. Plates were simultaneously lysed and stained using 100 μL of a SYBR Green I dye-lysis detergent solution. The added solution was incubated in plates at room atmosphere and ambient temperature in the dark for at least 1 h. Plate fluorescence was read with a Spectramax iD3 plate reader at 497 nm excitation and 520 nm emission. Fluorescence reads were normalized with respect to the untreated control wells representing normal parasite growth and plotted against the logarithm of drug concentration. A 50% growth inhibitory curve (IC_50_) was determined for each compound by fitting this data to a variable slope nonlinear regression curve using GraphPad Prism software (v.9).

#### Compound cytotoxicity against HepG2 cells (CC_50_)

All compounds were tested for cytotoxicity against immortalized human liver carcinoma cell line (HepG2) using previously described methods (21). Compounds were prepared as 10 mM stock solutions in DMSO, including a 10 mM DMSO stock of mefloquine to be used as a pos. Cultured human hepatocarcinoma (HepG2) cells in RPMI-1640 growth medium containing 10% fetal bovine serum at 37°C in a humidified 5% CO_2_ atmosphere were added at 2 × 10^4^ density to 96-well flat-bottomed tissue culture plates with an additional 160 µL of complete culture medium per well and incubated overnight at 37°C to allow for adherence. Forty microliter aliquots of compound from stock solutions diluted in complete medium were added to each well in duplicate as a serial dilution to capture a range of 200 μL through 0.20 μL. Drug-treated plates were incubated in 5% CO_2_ atmosphere at 37°C for 24 h, then aspirated and 200 μL of complete medium was added to each well. After an additional 24 h incubation, a 20 μL aliquot of PBS buffer with resazurin (Alamar Blue) was added to each well for a final concentration of 10 μM. After a 3 h incubation with resazurin, fluorescence was measured at 560 nm excitation and 590 nm emission bands using a SpectraMax iD3 plate reader. Fluorescence reads were normalized to untreated control wells and plotted against the logarithm of drug concentration. These data were fit to a variable slope nonlinear regression curve using GraphPad Prism software (v.9) to determine 50% cell cytotoxicity (CC_50_).

#### *Plasmodium yoelii* infection suppression

Select compounds were evaluated for *in vivo* efficacy in a murine malaria model at a fixed dose of 2.5 mg/kg/day. Stock solutions were made for dosing by dissolving compound in polyethylene glycol 400 (PEG-400) one day prior to experiment. Using a modified 4-day Peters test, female CF1 mice (*n* = 4) from Charles River Laboratories were inoculated intravenously with approximately 2.5–5.0 × 10^4^ infected erythrocytes (*Plasmodium yoelii*, lethal Kenya strain MR4 MRA-428) from an infected donor mouse on day 0. Over the next = 96 h (days 1–4), dosing solutions (PEG-400 only for control mice) were administered by oral gavage once daily. On day 5, parasite concentration in each mouse was determined by microscopic examination of Giemsa-stained blood smears gathered from the tail vein. Parasite suppression was calculated by comparing parasitemia of treated mice relative to untreated controls using GraphPad Prism (v.9) and reported as a percentage.

#### *Plasmodium yoelii* infection efficacy and clearance (ED_50_, ED_90_, and NRD)

Select compounds were evaluated for *in vivo* efficacy using a modified 4-day Peters test. Stock solutions were made for dosing by dissolving compound in polyethylene glycol 400 (PEG-400) on day 0. On day 0, female CF1 (*n* = 4/dosing value) mice from Charles River Laboratories were inoculated intravenously with approximately 2.5–5.0 × 10^4^ parasitized erythrocytes (*Plasmodium yoelii*, lethal Kenya strain MR4 MRA-428) from an infected donor mouse. Over the following 4 days (day 1–4), dosing solutions were administered by oral gavage once per day. Initially, compounds were assessed at 1.0, 2.5, 5.0, and 10 mg/kg/day, but additional dosage values were added if necessary to obtain an interpolated ED_50_ and ED_90_ value. On day 5, mouse erythrocyte parasite infection was determined by microscopic examination of Giemsa-stained blood smears collected from the tail vein. Animals with no observable parasitemia by microscopic analysis on day 5 were monitored, and parasitemia was determined twice weekly until parasites were observed or up to day 30. Mice were sacrificed when parasites were observed by microscopic analysis on or after day 5. *In vivo* efficacy against infection (ED_50_ and ED_90_) was determined by generating dose–response curves of the parasite concentration of treated mice relative to untreated controls using GraphPad Prism (v.9). NRD was determined as the minimum dose required to maintain 0% parasitemia by microscopic analysis until day 30.

#### Stability in pooled murine liver microsomes (t_1/2_ and CL_int_)

Select compounds were assessed for murine microsomal stability in pooled liver microsomes performed by ChemPartner in Shanghai, China. In short, compounds were incubated at 37°C and 1 μM concentration in murine liver microsomes (Corning) for 1 h at a protein concentration of 0.5 mg/mL in potassium phosphate buffer at pH 7.4 containing 1.0 mM EDTA. The metabolic reaction was initiated by addition of NADPH and quenched with ice-cold acetonitrile at 15 min increments up to 1 h. The progress of compound metabolism was followed by LC-MS/MS (ESI positive ion, LC-MS/MS-034(API-6500+) using a C18 stationary phase (ACQUITY UPLC BEH C18 [2.1 × 50 mm, 1.7 μm]) and a MeOH/water mobile phase containing 0.25% formic acid and 1 mM NH_4_OAc. Imipramine or Osalmid was used as internal standards, ketanserin was used as a metabolically unstable control compound. Concentration-versus-time data for each compound were fitted to an exponential decay function to determine the first-order rate constant for compound depletion, which was then used to calculate the degradation half-life (t_1/2_) and predicted intrinsic clearance value (Cl_int_) from an assumed murine hepatic blood flow of 90 mL/min/kg.

#### PK study of 25 in CF1 mice

The title compound was selected for a PK analysis in mice at an oral dose of 10 mg/kg and an intravenous dose of 0.5 mg/kg, and was performed by a Contract Research Organization (CRO). Three groups of three male CF1 mice (JH Laboratory Animal) were used in each arm of the study and were administered the drug in PEG-400 at 1 mg/mL orally by oral gavage and in PEG-400 at 0.1 mg/mL intravenously via tail vein. Single groups of mice were manually restrained and approximately 93 µL of blood was taken from the animals via facial vein for semi-serial bleeding into K_2_EDTA tubes at the following time points: 0.083 h, 0.25 h, 0.5 h, 1 h, 2 h, 4 h, 8 h, 24 h, 48 h, 72 h, 96 h, and 120 h post-dose administration. Samples were put on ice and centrifuged (2,000 × *g*, 5 min at under 4°C) within 15 min of collection. An aliquot of 10 µL sample was added to 20 µL 10mM NH_4_OAc and 3 µL internal standard (Diclofenac, 200 ng/mL) in ACN. The mixture was vortexed for 1 min, then added to 800 µL MTBE and continued to vortex for an additional 10 min followed by centrifugation at 14,000 rpm for 5 min. A 700 µL aliquot of the supernatant was condensed under nitrogen steam. Samples were reconstituted with 200 µL of 1:1 methanol/water and vortexed for 2 min. Finally, 1 µL of the prepared sample was injected into a LC-MS/MS-21 (Triple Quad 6500) and separated on a Waters ACQUITY UPLC HSS T3 (2.1 x 50 mm, 1.8 µm) column. PK analysis as a best-fit curve was prepared from drug concentration in plasma as a function of time using non-compartmental analysis as implemented in WinNonlin software (Pharsight, Mountain View, CA). The exposure (AUC_last_), half-life (T_1/2_), maximum concentration (*C*_max_), and time of maximum concentration (*T*_max_) will be determined from data. Goodness-of-fit was assessed by the *r*^2^ (linear regression coefficient) of the drug concentration on the terminal phase. Bioavailability (F) was calculated using equation 3.

### Equations

1. Log of selectivity index (pSI)


(1) $$pSI\;=\log_{10}\left(\frac{HepG2\;CC_{50}\left(nM\right)}{PfD6\;IC_{50}\;\left(nM\right)}\right)$$

2. Predicted hepatic extraction ratio (E_H_)

Each degradation half-life (*t*_1/2_) was used to calculate [2a] a predicted *in vivo* hepatic clearance value (CL_int_) before [2b] a predicted *in vivo* hepatic extraction ratio (E_H_).


(2a) $$CL_{int}=\;\left(\frac{\ln2}{t_{1/2}}\right)\times \left(\frac{1}{(microsomal\;protien\;\left[conc\right]\;\left(mg/mL\right)}\right)\times \left(Scaling\;Factor\right)$$

(2b) $$E_{H}=\;\frac{CL_{blood}}{Q}=\;\frac{CL_{int}}{Q+\;CL_{int}}$$

Microsomal protein concentration = (0.5 mg/mL). Scaling factor for mice = (microsomal protein per gram of liver = 45 mg/g) × (liver weight per gram of body weight = 87.5 g/kg) = 3,937.5. Hepatic blood flow (Q) of mice = 90 (mL/min/kg).

E_H_ classifications are based on the following assumptions, outlined in Obach (22):

1. NADPH-dependent oxidative metabolism predominates other metabolic routes.
2. Rates of *in vitro* metabolism and enzymatic activity are reflective of those *in vivo*.

Calculations of CL_int_ are based on the “*in vitro* t_1/2_ method” (22), which utilizes the following assumptions:

3. Bioavailability


(3) $$F=100\cdot \frac{AUC_{po}\cdot D_{iv}}{AUC_{iv}\cdot D_{po}}$$

AUC is area under the curve for oral (po) and tail vein (iv) and D is dose for oral (po) and tail vein (iv).

## SUPPLEMENTAL MATERIAL

Supplemental Material aac.01300-25-s0001.pdf Synthetic details and characterization of select compounds; Fig. S1 to S39.

Graphical abstract aac.01300-25-s0002.tiff Visual depiction of the study.

ASM does not own the copyrights to Supplemental Material that may be linked to, or accessed through, an article. The authors have granted ASM a non-exclusive, world-wide license to publish the Supplemental Material files. Please contact the corresponding author directly for reuse.
