# Supplementary material for: Designing novel bisquinoline antimalarials from historical 4-aminoquinolines to combat drug-resistant malaria
Source: Antimicrob Agents Chemother. 2026 Mar 2;70(4):e01300-25. doi: 10.1128/aac.01300-25 (PMC13041310; doi:10.1128/aac.01300-25)
Supplement: Supplemental Material — Synthetic details and characterization of select compounds; Fig. S1 to S39. [file aac.01300-25-s0001.pdf]

Supporting Information

Designing Novel Bisquinoline Antimalarials from Historical 4-Aminoquinolines to Combat Drug-Resistant Malaria

Authors:

Mason J. Handford[a], Yuexin Li[b], Teresa Riscoe[b], Xiaowei Zhang[b], Jane X. Kelly[b,c] and Michael K. Riscoe[b,d]#.

[a]Chemical Physiology and Biochemistry Department, Oregon Health & Science University, 3181 SW Sam Jackson Boulevard, Portland, Oregon, USA.

[b]Experimental Chemotherapy Lab, Portland VA Medical Center, 3710 SW US Veterans Hospital Road, Portland, Oregon, USA.

[c]Department of Chemistry, Portland State University, 1719 SW 10th Avenue, Portland, Oregon, USA.

[d]Molecular Microbiology and Immunology Department, Oregon Health & Science University, 3181 SW Sam Jackson Boulevard, Portland, Oregon, USA.

#Address Correspondence to **Michael K. Riscoe** (riscoem@ohsu.edu)

Table of contents

Synthetic Details and Characterization of Select Compounds . . . S3
^{1}H NMR Spectra of Presented Compounds . . . S6
HRAM/MS Chromatograms of Presented Compounds . . . S29

List of Supplementary Figures

Figure S1: ^{1}H NMR spectra of **7**. . . . S6
Figure S2: ^{1}H NMR spectra of **8**. . . . S7
Figure S3: ^{1}H NMR spectra of **9**. . . . S8
Figure S4: ^{1}H NMR spectra of **10**. . . . S9
Figure S5: ^{1}H NMR spectra of **11**. . . . S10
Figure S6: ^{1}H NMR spectra of **12**. . . . S11
Figure S7: ^{1}H NMR spectra of **13**. . . . S12
Figure S8: ^{1}H NMR spectra of **14**. . . . S13
Figure S9: ^{1}H NMR spectra of **15**. . . . S14
Figure S10: ^{1}H NMR spectra of **17**. . . . S15
Figure S11: ^{1}H NMR spectra of **18**. . . . S16
Figure S12: ^{1}H NMR spectra of **21a intermediate**. . . . S17
Figure S13: ^{1}H NMR spectra of **21b intermediate**. . . . S18
Figure S14: ^{1}H NMR spectra of **21c intermediate**. . . . S19
Figure S15: ^{1}H NMR spectra of **21a**. . . . S20
Figure S16: ^{1}H NMR spectra of **21b**. . . . S21
Figure S17: ^{1}H NMR spectra of **21c**. . . . S22
Figure S18: ^{1}H NMR spectra of **23**. . . . S23
Figure S19: ^{1}H NMR spectra of **24**. . . . S24
Figure S20: ^{1}H NMR spectra of **25**. . . . S25
Figure S21: ^{1}H NMR spectra of **26**. . . . S26
Figure S22: ^{1}H NMR spectra of **27**. . . . S27
Figure S23: ^{1}H NMR spectra of **28**. . . . S28
Figure S24: HRAM/MS Chromatogram of **8**. . . . S29
Figure S25: HRAM/MS Chromatogram of **9**. . . . S30
Figure S26: HRAM/MS Chromatogram of **10**. . . . S31
Figure S27: HRAM/MS Chromatogram of **11**. . . . S32
Figure S28: HRAM/MS Chromatogram of **12**. . . . S33
Figure S29: HRAM/MS Chromatogram of **13**. . . . S34
Figure S30: HRAM/MS Chromatogram of **14**. . . . S35
Figure S31: HRAM/MS Chromatogram of **15**. . . . S36
Figure S32: HRAM/MS Chromatogram of **17**. . . . S37
Figure S33: HRAM/MS Chromatogram of **18**. . . . S38
Figure S34: HRAM/MS Chromatogram of **23**. . . . S39
Figure S35: HRAM/MS Chromatogram of **24**. . . . S40
Figure S36: HRAM/MS Chromatogram of **25**. . . . S41
Figure S37: HRAM/MS Chromatogram of **26**. . . . S42
Figure S38: HRAM/MS Chromatogram of **27**. . . . S43
Figure S39: HRAM/MS Chromatogram of **28**. . . . S44

Synthetic Details and Characterization of Select Compounds

Structure of **7**

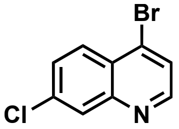

4-bromo-7-chloroquinoline (**7**). The title compound was prepared as described. In short, 20.20 g (112 mmol) of 7-chloro-4-hydroxyquinoline was charged to a 500 mL round bottom flask containing 250 mL DMF (0.45 M). Flask was equipped with a stir bar and placed in an ice bath, followed by the slow addition of phosphorus tribromide (12 mL, 1.1 equivalence) using an addition funnel and stirred overnight. Reaction mixture was decanted onto ice and saturated sodium bicarbonate was added until pH > 10. Crude product was extracted in a separatory funnel with 3 x 100 mL washes of ethyl acetate. Organic layers were combined and washed in a separatory funnel with water (2 x 200 mL), followed by aqueous saturated sodium chloride (2 x 100 mL). Resulting organic layer dried with magnesium sulfate and gravity filtered, the filtrate was then condensed in rotary evaporator. Crude material was recrystallized in hot ethyl acetate to form final product as white needle-like crystals. Yield = 80% (21.76 grams, 89.7 mmol). 1H-NMR (400 MHz; DMSO-d_6) ppm: 8.78 (d, J = 4.7 Hz, 1H), 8.19 (d, J = 7.2 Hz, 1H), 8.18 (s, 1H), 8.00 (d, J = 4.7 Hz, 1H), 7.82 (dd, J = 8.9, 2.3 Hz, 1H).

Structure of **21a intermediate**

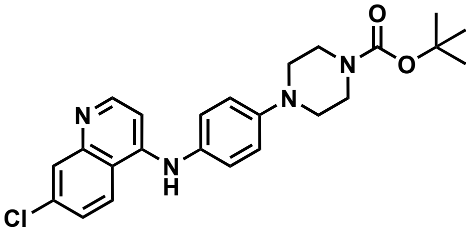

tert-butyl 4-(4-((7-chloroquinolin-4-yl)amino)phenyl)piperidine-1-carboxylate (**21a intermediate**). The title compound was prepared using General Procedure (ii). In short, 1.150 g (5.80 mmol) 7-chlor0-4-bromoquinoline, 1.603 g (5.80 mmol, 1 equiv.) 4-(aminophenyl)-piperadine-1-Boc, and 10 mL tertrohydrofuran (0.58 M) were charged to a 20 mL microwave reactor vial equipped with a magnetic stir bar. Reaction vessel sealed with septa cap and reaction heated to 120 °C for 45 minutes with high adsorption in a microwave reactor. Resulting yellow solid was filtered and washed with tetrahydrofuran (4 x 10 mL). Solid was dissolved in 150 mL 1M NH_3 in methanol and solids triturated with additional 200 mL methanol (4 x 50 mL). Resulting solid dried and dissolved in 400 mL DCM, and washed with 2 M NaOH (3 x 150 mL), water (3 x 100 mL), and 200 mL saturated sodium chloride. Resulting organic layer dried with magnesium sulfate, gravity filtered and dried. Final product isolated as a white solid. Yield = 87%, 1.924 g (4.40 mmol). ^{1}H NMR (DMSO-d_6, 400 MHz) ppm: 9.03 (s, 1H), 8.44 (d, 1H, J = 5.4 Hz), 8.42 (d, 1H, J = 9.3 Hz), 7.88 (d, 1H, J = 2.3 Hz), 7.56 (dd, 1H, J = 2.3, 9.0 Hz), 7.30 (s, 4H), 6.86 (d, 1H, J = 5.4 Hz), 4.09 (br d, 2H, J = 11.8 Hz), 2.82 (br s, 1H), 2.6-2.8 (m, 1H), 1.79 (br d, 2H, J = 12.5 Hz), 1.4-1.6 (m, 3H), 1.42 (s, 9H)

Structure of **21b intermediate**

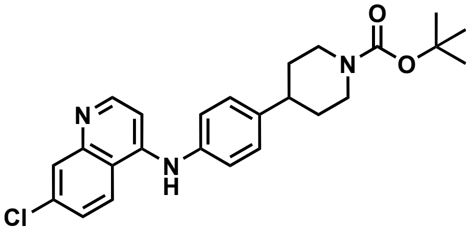

tert-butyl 4-(4-((7-chloroquinolin-4-yl)amino)phenyl)piperazine-1-carboxylate (**21b intermediate**). The title compound was prepared using General Procedure (ii). In short, 0.523 g (2.20 mmol) 4-bromoquinoline, 0.691 g (2.50 mmol) 4-(aminophenyl)-piperadine-1-Boc, and 5 mL tertrohydrofuran (0.44 M) were charged to a 20 mL microwave reactor vial equipped with a magnetic stir bar. Reaction vessel sealed with septa cap and reaction heated to 120 °C for 45 minutes with high adsorption in a microwave reactor. Resulting solid was filtered and washed with tetrahydrofuran (4 x 5 mL). Solid was dissolved in 150 mL methanol to which 50 mL of 2M sodium hydroxide was added and stirred for 1.5 hours. Solution was condensed *in vacuo*, filtered, washed with water and left to dry overnight. Final product isolated as a white solid. Yield = 80%, 0.714 g (1.77 mmol). ^{1}H NMR (400 MHz, CHLOROFORM-*d*) ppm: 8.49 (d, J = 5.25 Hz, 1 H) 8.01 (d, J = 2.13 Hz, 1 H) 7.84 (d, J = 9.01 Hz, 1 H) 7.44 (dd, J = 9.01, 2.13 Hz, 1 H) 7.18 - 7.24 (m, 2 H) 6.96 - 7.02 (m, 2 H) 6.71 (d, J = 5.25 Hz, 1 H) 6.51 (s, 1 H) 3.56 - 3.67 (m, 4 H) 3.12 - 3.21 (m, 5 H) 1.46 - 1.52 (m, 9 H).

Structure of **21c intermediate**

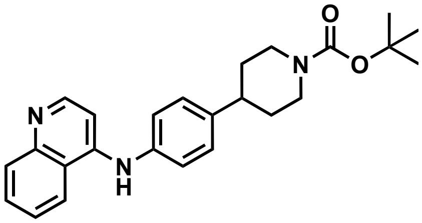

tert-butyl 4-(4-(quinolin-4-ylamino)phenyl)piperidine-1-carboxylate (**21c intermediate**). The title compound was prepared using *General Procedure (ii).* In short, 0.523 g (2.20 mmol) 4-bromoquinoline, 0.691 g (2.50 mmol) 4-(aminophenyl)-piperadine-1-Boc, and 5 mL tertrohydrofuran (0.44 M) were charged to a 20 mL microwave reactor vial equipped with a magnetic stir bar. Reaction vessel sealed with septa cap and reaction heated to 120 °C for 45 minutes with high adsorption in a microwave reactor. Resulting solid was filtered and washed with tetrahydrofuran (4 x 5 mL). Solid was dissolved in 150 mL methanol to which 50 mL of 2M sodium hydroxide was added and stirred for 1.5 hours. Solution was condensed *in vacuo*, then filtered and left to dry overnight. Final product isolated as a white solid. Yield = 80%, 0.714 g (1.77 mmol). ^{1}H NMR (DMSO-d_6, 400 MHz) ppm: 8.88 (s, 1H), 8.43 (d, 1H, J = 5.3 Hz), 8.37 (d, 1H, J = 8.5 Hz), 7.86 (d, 1H, J = 8.0 Hz), 7.68 (t, 1H, J = 7.3 Hz), 7.52 (t, 1H, J = 7.3 Hz), 7.29 (s, 4H), 6.87 (d, 1H, J = 5.3 Hz), 4.0-4.2 (m, 2H), 3.17 (d, 1H, J = 5.1 Hz), 2.82 (br s, 1H), 2.70 (tt, 1H, J = 3.2, 11.9 Hz), 1.79 (br d, 2H, J = 12.3 Hz), 1.4-1.6 (m, 2H), 1.43 (s, 9H).

Structure of **21a**

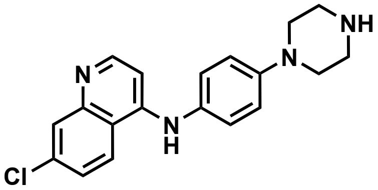

7-chloro-N-(4-(piperidin-4-yl)phenyl)quinolin-4-amine (**21a**). The title compound was prepared using *General Procedure (iii).* In short, 4.440 g (10.1 mmol) of **21a intermediate** was charged to a 250 mL round-bottom flask equipped with a magnetic stir bar and suspended in 100 mL dichloromethane. Slowly 23 mL (303 mmol, 30 equiv.) trifluoroacetic acid was added drop-wise while stirring using an addition funnel. As TFA was added initial turgid suspension went fully into solution. After 4 h, remaining TFA was neutralized with 200 mL of 2M sodium hydroxide solution and until pH > 10. Resulting white precipitate was filtered, and washed with water (3 x 50 mL), ethyl acetate (3 x 50 mL), and DCM (3 x 25 mL). Solid was dried overnight in vacuum oven. Final product isolated as a white solid. Yield = 97%, 3.310 g (9.80 mmol). ^{1}H NMR (CHLOROFORM-*d*, 400 MHz) ppm: 8.54 (d, 1H, J = 5.3 Hz), 8.03 (d, 1H, J = 2.1 Hz), 7.85 (d, 1H, J = 9.0 Hz), 7.45 (dd, 1H, J = 2.2, 8.9 Hz), 7.3-7.3 (m, 2H), 7.2-7.3 (m, 2H), 6.91 (d, 1H, J = 5.3 Hz), 6.58 (s, 1H), 3.22 (br d, 2H, J = 11.8 Hz), 2.77 (dt, 2H, J = 2.3, 12.1 Hz), 2.65 (s, 1H), 1.8-1.9 (m, 2H), 1.7-1.7 (m, 2H).

Structure of **21b**

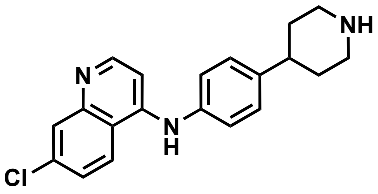

7-chloro-N-(4-(piperazin-1-yl)phenyl)quinolin-4-amine (**21b**). The title compound was prepared using *General Procedure (iii).* In short, 1.501 g (3.42 mmol) of **21b intermediate** was charged to a 50 mL round-bottom flask equipped with a magnetic stir bar and suspended in 10 mL dichloromethane. Slowly 4 mL (52.6 mmol, 15 equiv.) trifluoroacetic acid was added dropwise while stirring using a glass dropper. As TFA was added inital turgid suspension went fully into solution. After 4 h, remaining TFA was neutalized with 100 mL of 2M sodium hydroxide solution and until pH > 10. Resulting white precipitate was filtered, and washed with water (3 x 5 mL), ethyl acetate (3 x 5 mL), and DCM (3 x 10 mL). Solid was dried overnight in vacuum oven. Final product isolated as a yellow solid. Yield = 40%, 0.464 g (1.37 mmol). ^{1}H NMR (400 MHz, DMSO-d_6) ppm: 8.91 (s, 1 H) 8.87 - 8.96 (m, 1 H) 8.41 (d, J = 9.01 Hz, 1 H) 8.37 (d, J = 5.38 Hz, 1 H) 7.84 (d, J = 2.25 Hz, 1 H) 7.52 (dd, J = 9.01, 2.25 Hz, 1 H) 7.19 (d, J = 8.88 Hz, 2 H) 6.94 - 7.07 (m, 2 H) 6.62 (d, J = 5.38 Hz, 1 H) 2.99 - 3.13 (m, 4 H) 2.77 - 2.91 (m, 4 H).

Structure of **21c**

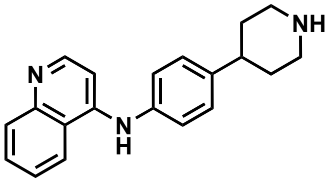

N-(4-(piperidin-4-yl)phenyl)quinolin-4-amine (**21c**). The title compound was prepared using *General Procedure (iii).* In short, 0.6493 g (1.61 mmol) of **21c intermediate** were charged to a 100 mL Erlenmeyer flask equipped with a magnetic stir bar and suspended in 30 mL dichloromethane. Slowly 3.0 mL (39.2 mmol, 25 equiv.) trifluoroacetic acid was added dropwise while stirring using a glass dropper. As TFA was added inital turgid suspension went fully into solution. After 4 h, remaining TFA was neutalized with 50 mL of 2M sodium hydroxide solution and until pH > 10. Resulting white precipitate was filtered, and washed with water (3 x 5 mL), ethyl acetate (3 x 5 mL), and DCM (3 x 10 mL). Solid was dried overnight in vacuum oven. Final product isolated as a yellow/straw-colored solid. Yield = 81%, 0.396 g (1.31 mmol). ^{1}H NMR (DMSO-d_6, 400 MHz) ppm: 8.89 (br s, 1H), 8.43 (d, 1H, J = 5.3 Hz), 8.38 (d, 1H, J = 8.4 Hz), 7.86 (d, 1H, J = 8.4 Hz), 7.69 (t, 1H, J = 7.6 Hz), 7.52 (t, 1H, J = 7.6 Hz), 7.29 (q, 4H, J = 8.6 Hz), 6.87 (d, 1H, J = 5.3 Hz), 3.1-3.2 (m, 3H), 2.6-2.8 (m, 2H), 2.0-2.1 (m, 1H), 1.8-1.9 (m, 2H), 1.5-1.7 (m, 2H).

^{1}H NMR Spectra of Presented Compounds

Figure S1: ^{1}H NMR spectra of **7**.

^{1}H NMR (400 MHz, DMSO-d_6) δ ppm 8.77 (d, *J*=4.63 Hz, 1 H) 8.19 (d, *J*=7.38 Hz, 1 H) 8.17 (s, 1 H) 8.00 (d, *J*=4.75 Hz, 1 H) 7.82 (d, *J*=9.00 Hz, 1 H)

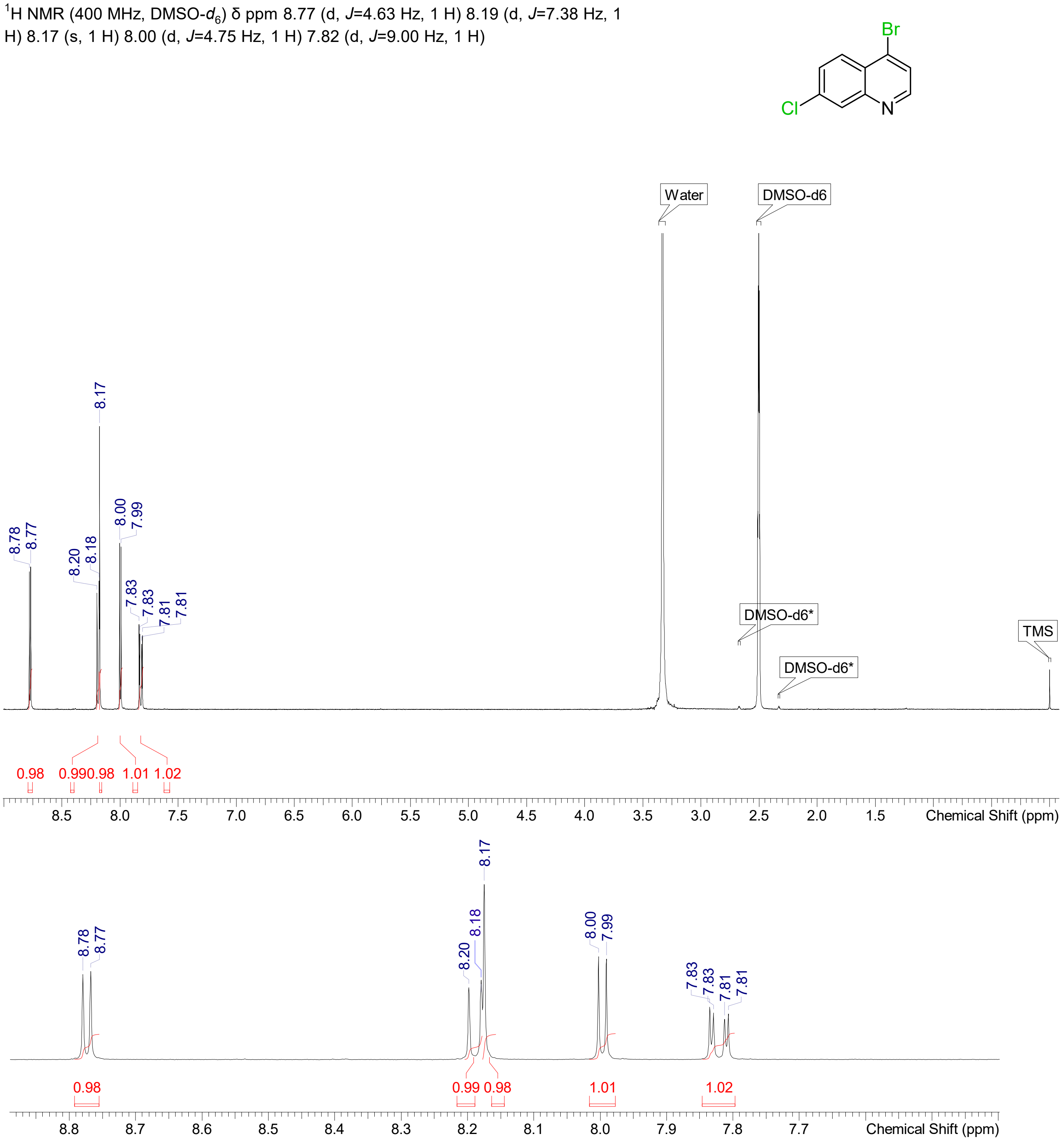

Figure S2: ^{1}H NMR spectra of **8**.

^{1}H NMR (400 MHz, DMSO-d_6) δ ppm 9.26 (s, 1 H) 8.57 (d, *J*=5.38 Hz, 1 H) 8.52 (d, *J*=9.13 Hz, 1 H) 7.98 (d, *J*=2.25 Hz, 1 H) 7.83 (d, *J*=8.50 Hz, 3 H) 7.66 (dd, *J*=9.01, 2.25 Hz, 2 H) 7.53 (d, *J*=8.63 Hz, 3 H) 7.11 (d, *J*=5.25 Hz, 1 H)

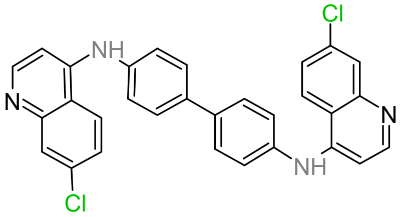

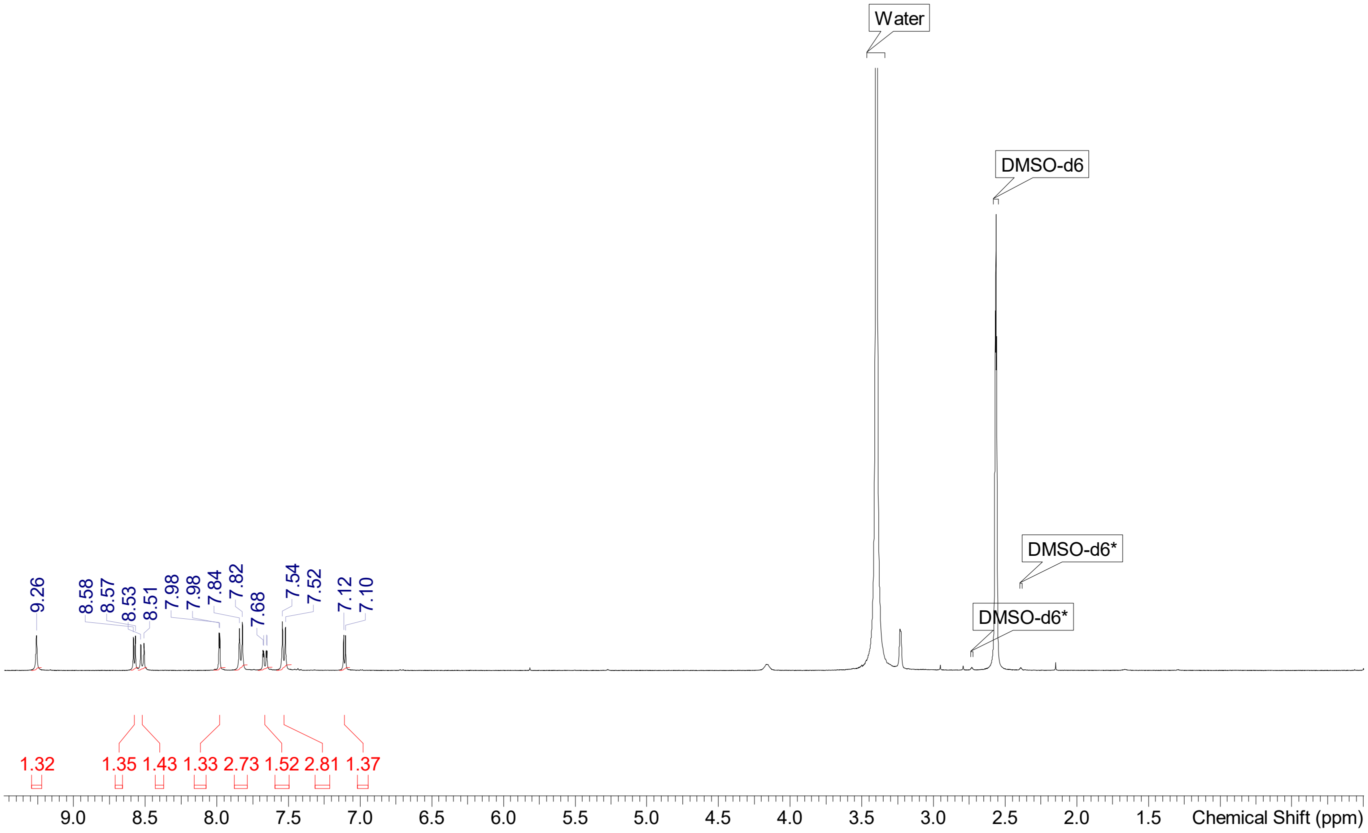

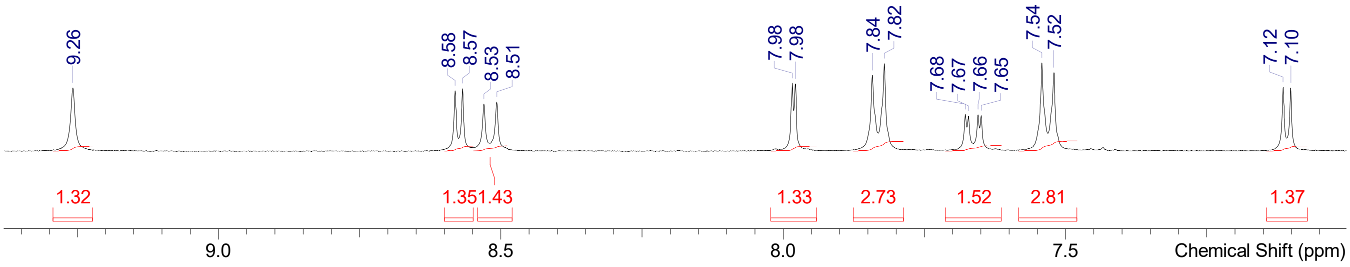

Figure S3: ^{1}H NMR spectra of **9**.

^{1}H NMR (400 MHz, DMSO-d_6) δ ppm 10.96 (br s, 2 H) 8.74 (d, *J*=9.13 Hz, 2 H) 8.67 (d, *J*=6.88 Hz, 2 H) 8.09 (d, *J*=2.00 Hz, 2 H) 7.97 (dd, *J*=9.13, 2.00 Hz, 2 H) 7.74 (dt, *J*=8.41, 4.36 Hz, 2 H) 7.56 - 7.64 (m, 2 H) 7.52 (dd, *J*=8.25, 2.00 Hz, 2 H) 7.15 (d, *J*=6.88 Hz, 2 H)

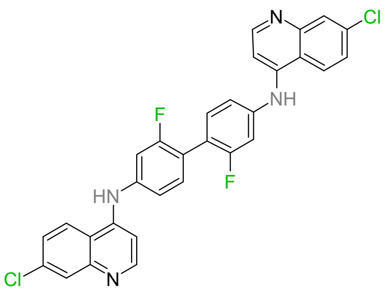

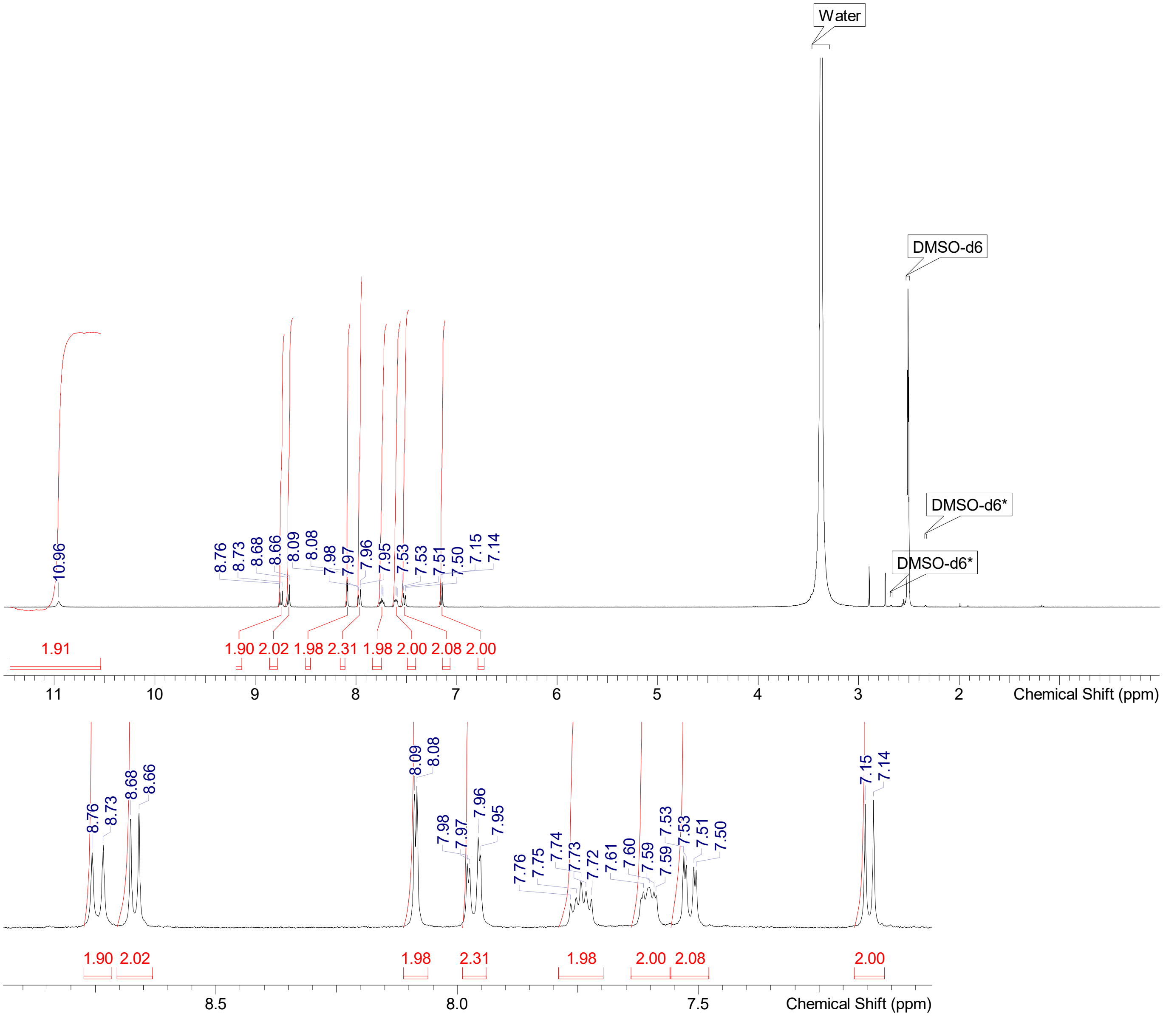

Figure S4: ^{1}H NMR spectra of **10**.

^{1}H NMR (400 MHz, DMSO-d_6) δ ppm 9.95 (br s, 2 H) 8.56 (d, *J*=9.13 Hz, 2 H) 8.48 (d, *J*=6.13 Hz, 2 H) 7.95 (d, *J*=2.13 Hz, 2 H) 7.74 (dd, *J*=9.01, 1.88 Hz, 2 H) 7.40 (q, *J*=8.59 Hz, 8 H) 6.85 (d, *J*=6.25 Hz, 2 H) 4.06 (s, 2 H)

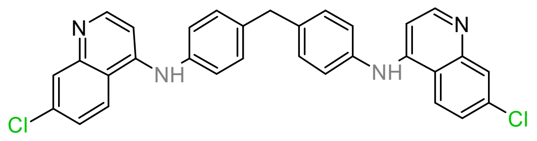

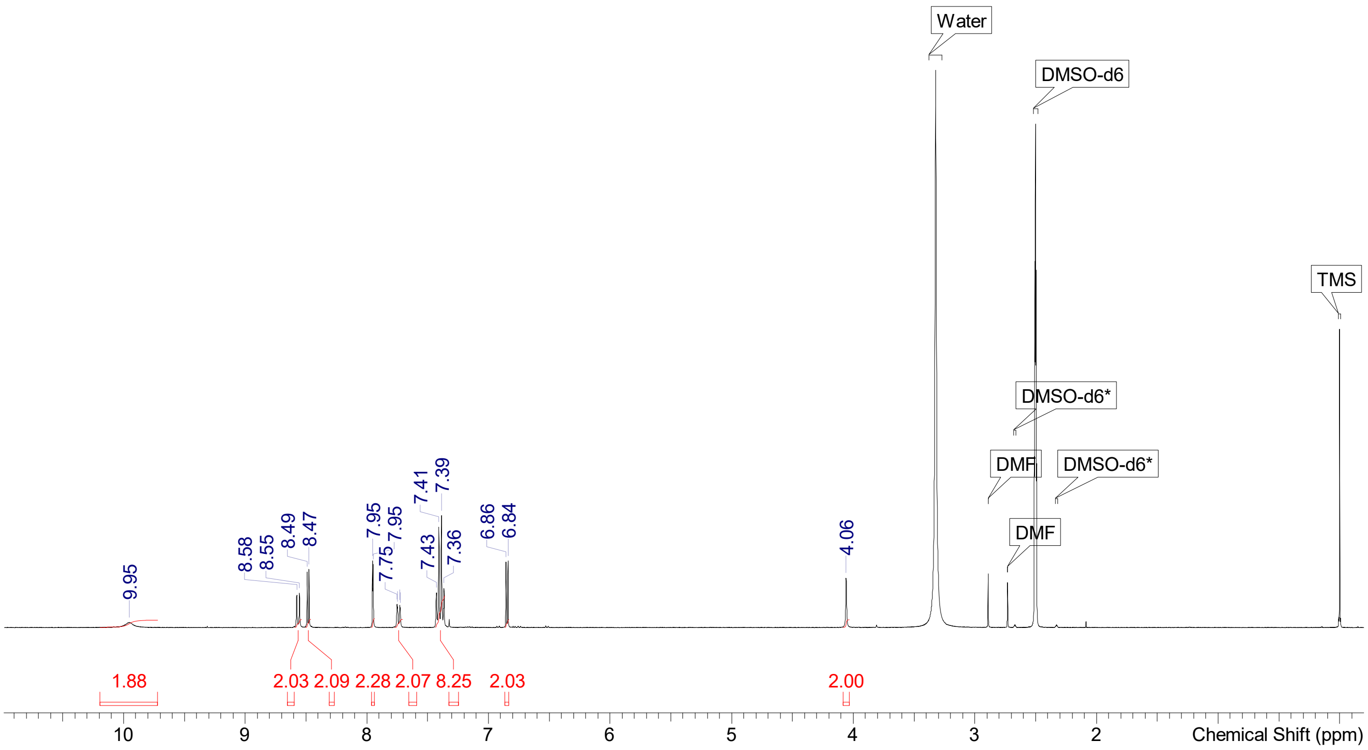

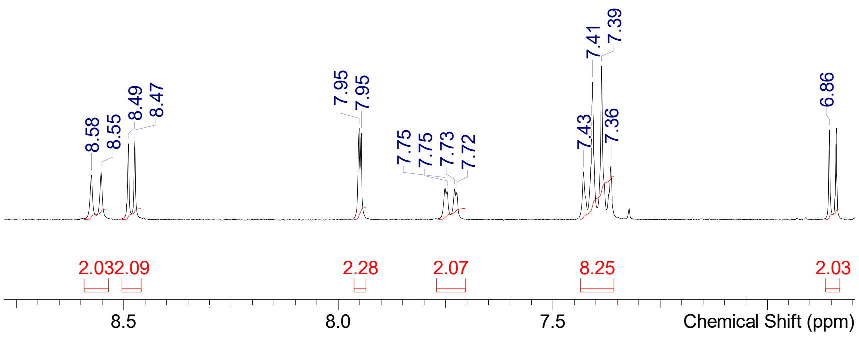

Figure S5: ^{1}H NMR spectra of **11**.

^{1}H NMR (400 MHz, DMSO-d_6) δ ppm 8.98 (s, 2 H) 8.38 - 8.46 (m, 4 H) 8.30 (s, 1 H) 7.87 (d, J=2.13 Hz, 2 H) 7.55 (dd, J=9.01, 2.25 Hz, 2 H) 7.22 - 7.28 (m, 4 H) 7.16 - 7.21 (m, 4 H) 6.70 (d, J=5.38 Hz, 2 H)

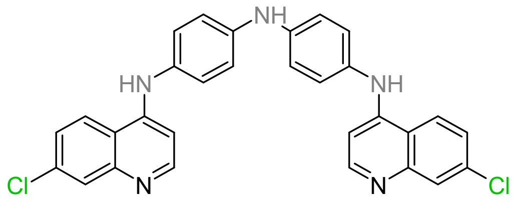

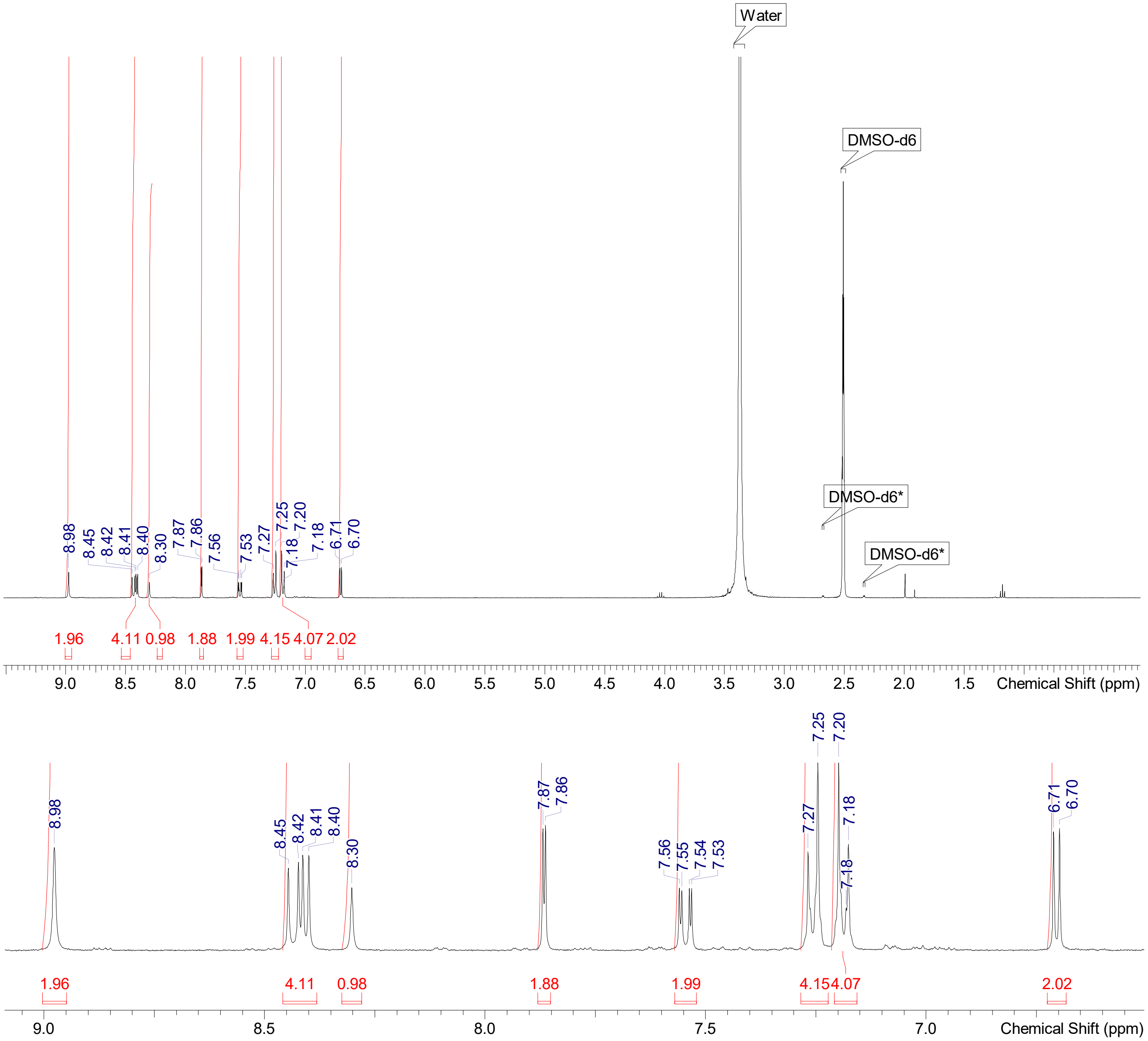

Figure S6: ^{1}H NMR spectra of **12**.

^{1}H NMR (400 MHz, DMSO-d_6) δ ppm 9.09 (s, 1 H) 8.41 - 8.48 (m, 2 H) 7.89 (d, J=2.25 Hz, 1 H) 7.58 (dd, J=9.07, 2.19 Hz, 1 H) 7.37 - 7.43 (m, 2 H) 7.10 - 7.19 (m, 2 H) 6.84 (d, J=5.38 Hz, 1 H)

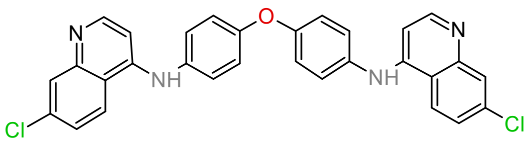

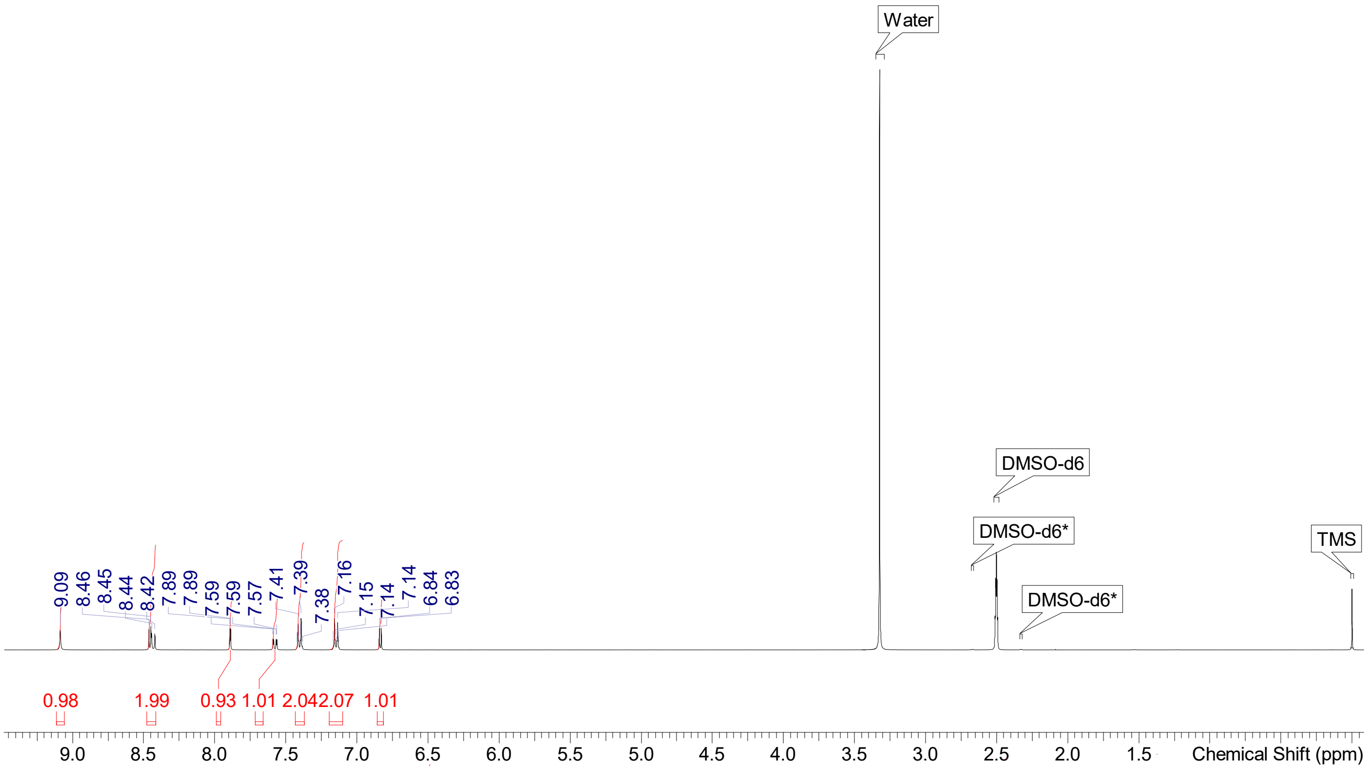

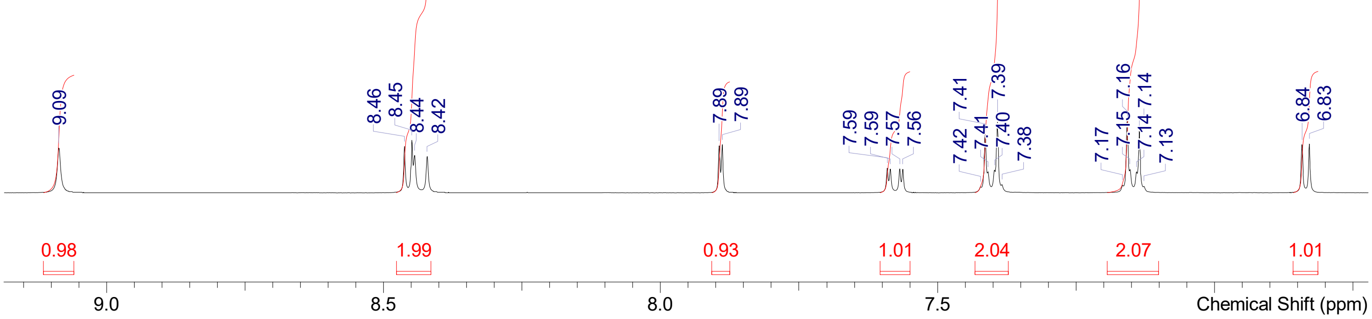

Figure S7: ^{1}H NMR spectra of **13**.

^{1}H NMR (400 MHz, DMSO-d_6) δ ppm 9.05 (s, 2 H) 8.40 - 8.47 (m, 4 H) 7.89 (d, J=2.13 Hz, 2 H) 7.57 (dd, J=9.01, 2.25 Hz, 2 H) 7.28 - 7.36 (m, 8 H) 6.91 (d, J=5.38 Hz, 2 H) 1.71 (s, 6 H)

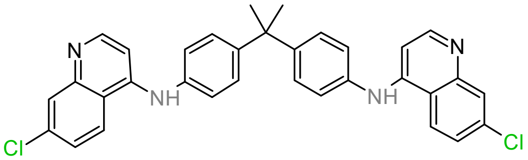

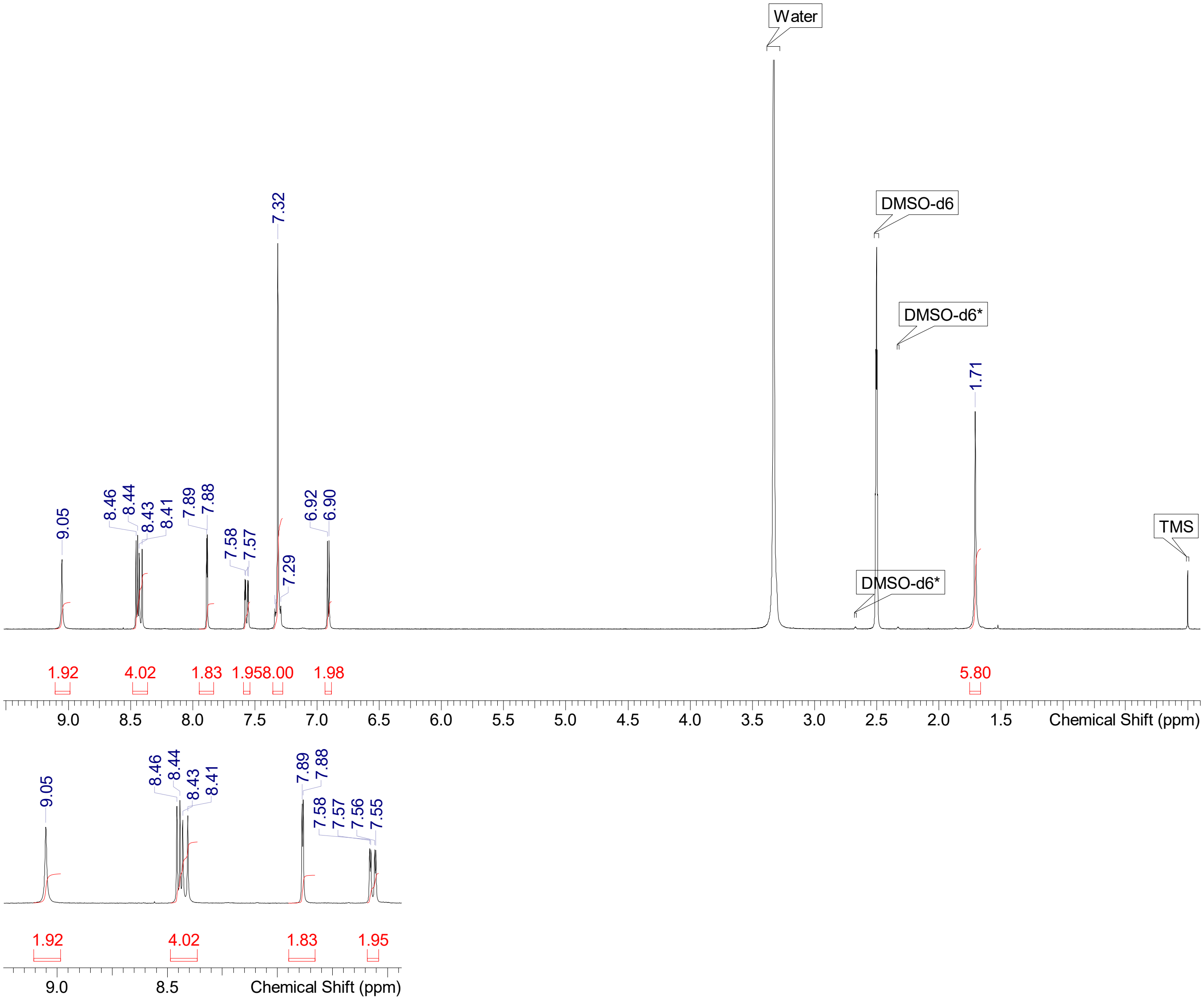

Figure S8: ^{1}H NMR spectra of **14**.

^{1}H NMR (400 MHz, DMSO-d_6) δ ppm 11.44 (s, 1 H) 9.17 (s, 2 H) 8.50 (d, *J*=9.13 Hz, 2 H) 8.36 (d, *J*=5.38 Hz, 2 H) 8.13 (d, *J*=1.88 Hz, 2 H) 7.86 (d, *J*=2.25 Hz, 2 H) 7.60 (d, *J*=8.50 Hz, 2 H) 7.52 - 7.58 (m, 2 H) 7.36 - 7.44 (m, 2 H) 6.64 (d, *J*=5.38 Hz, 2 H)

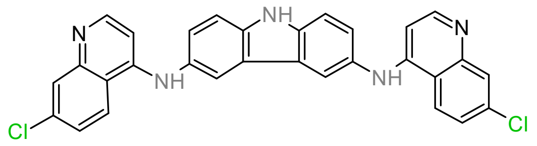

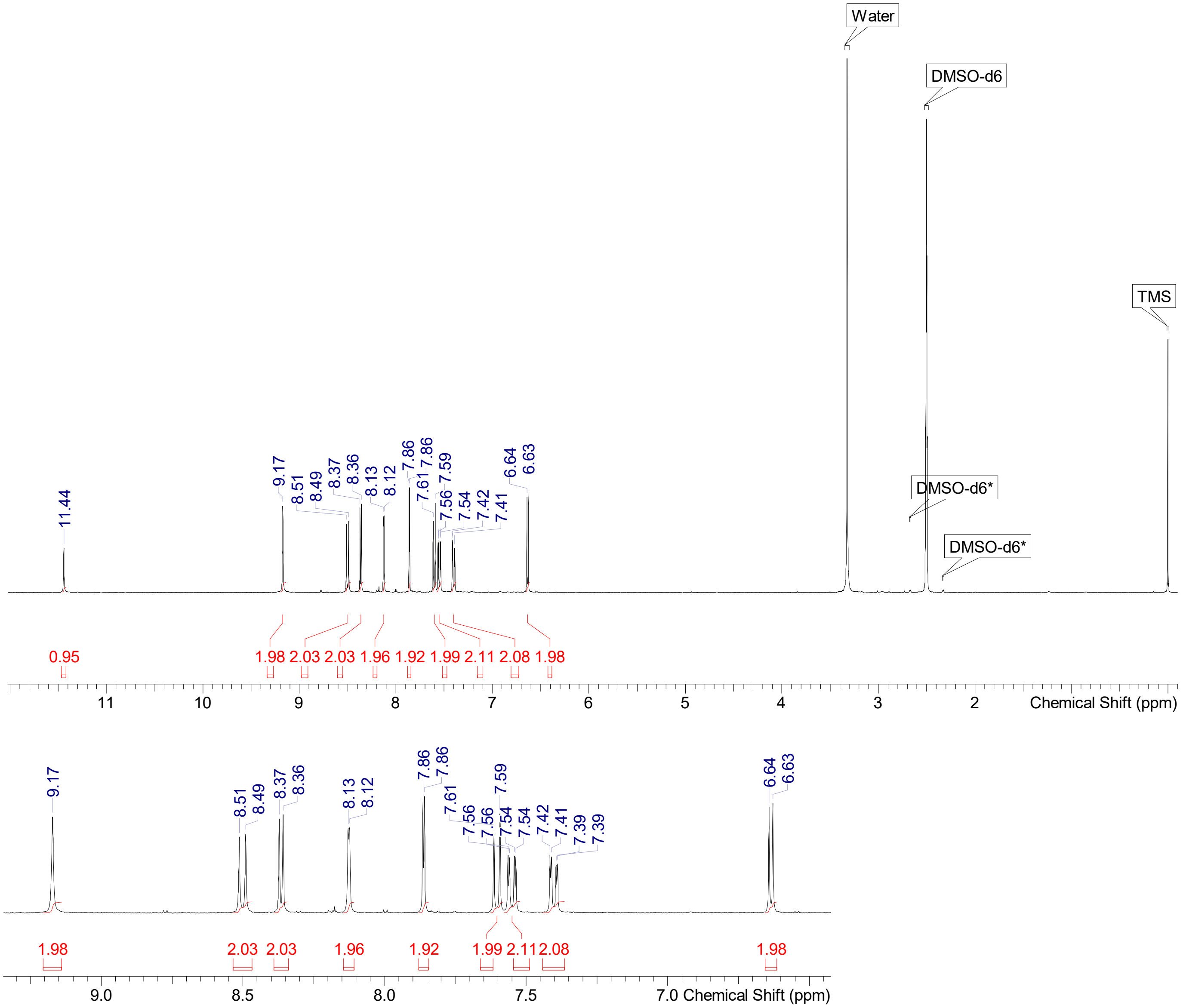

Figure S9: ^{1}H NMR spectra of **15**.

^{1}H NMR (400 MHz, DMSO-d_6) δ ppm 9.30 (br s, 2 H) 8.53 (br d, J=5.25 Hz, 2 H) 8.40 (d, J=9.00 Hz, 2 H) 7.93 (d, J=2.00 Hz, 2 H) 7.76 (d, J=2.25 Hz, 2 H) 7.69 - 7.74 (m, 2 H) 7.63 (dd, J=9.07, 2.06 Hz, 2 H) 7.23 (d, J=8.76 Hz, 2 H) 7.01 (br d, J=5.00 Hz, 2 H)

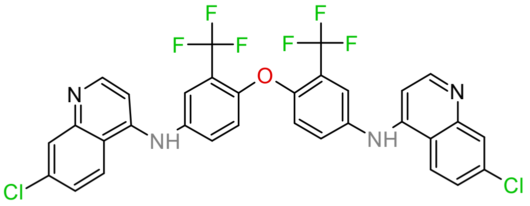

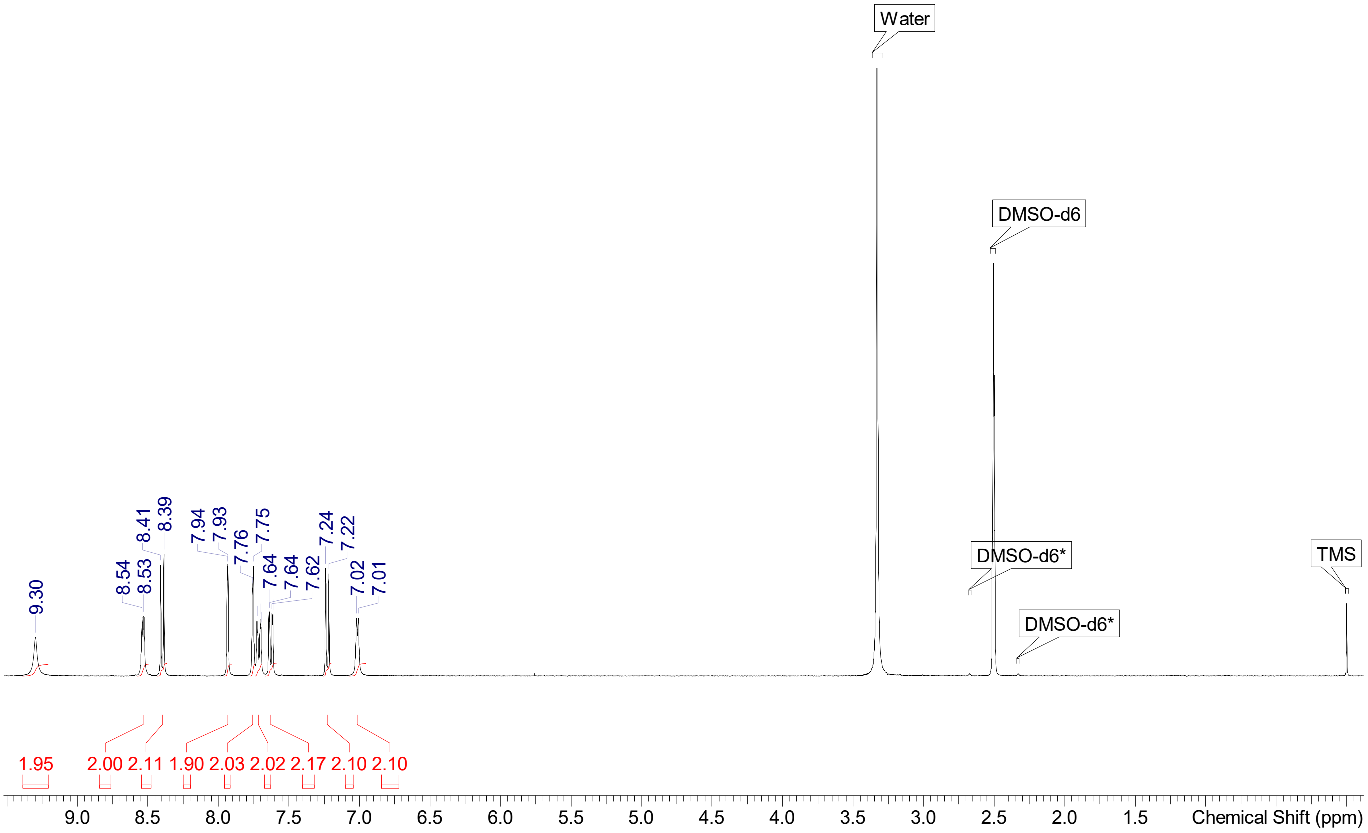

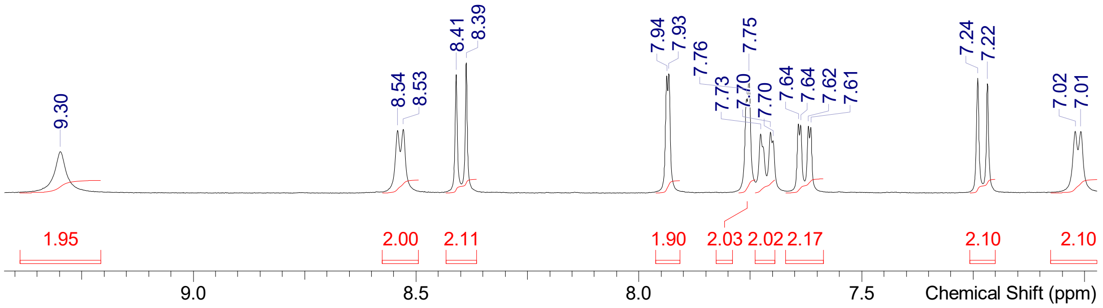

Figure S10: ^{1}H NMR spectra of **17**.

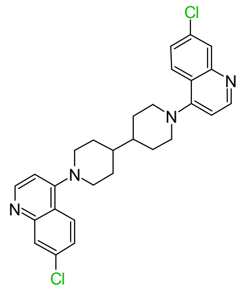

^{1}H NMR (400 MHz, DMSO-d_6) δ ppm 8.69 (d, J=5.00 Hz, 2 H) 8.02 (d, J=9.01 Hz, 2 H) 7.97 (d, J=2.00 Hz, 2 H) 7.57 (dd, J=8.94, 1.94 Hz, 2 H) 7.02 (d, J=5.13 Hz, 2 H) 3.63 (br d, J=12.13 Hz, 5 H) 2.85 (br t, J=11.63 Hz, 5 H) 1.94 (br d, J=12.13 Hz, 4 H) 1.52 - 1.68 (m, 4 H) 1.47 (br d, J=8.63 Hz, 2 H)

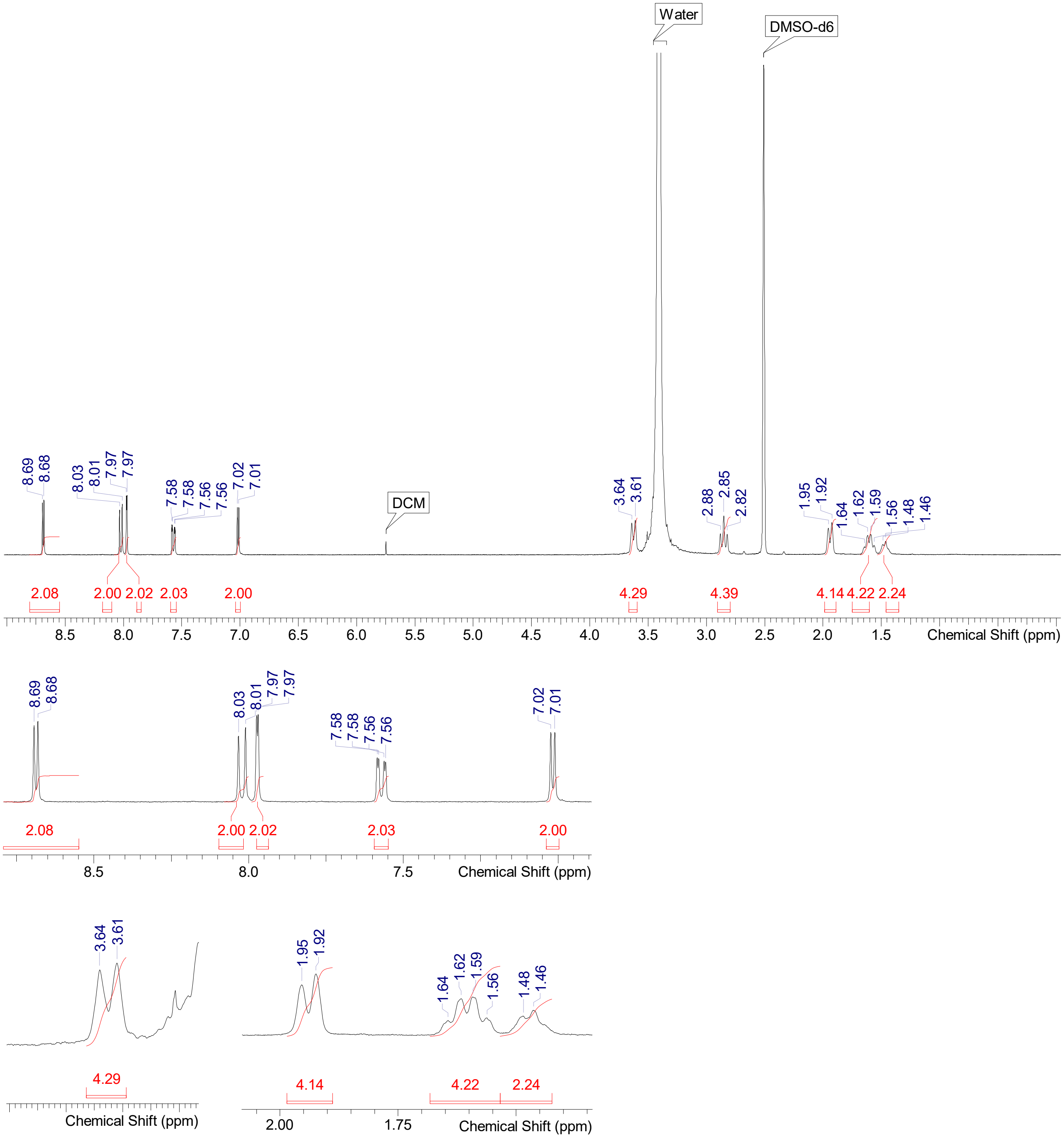

Figure S11: ^{1}H NMR spectra of **18**.

^{1}H NMR (400 MHz, DMSO-d_6) δ ppm 8.68 (d, *J*=5.00 Hz, 2 H) 7.93 - 8.01 (m, 4 H) 7.55 (dd, *J*=9.01, 2.25 Hz, 2 H) 6.98 (d, *J*=5.00 Hz, 2 H) 3.53 (br d, *J*=12.13 Hz, 4 H) 2.81 (br t, *J*=11.26 Hz, 4 H) 1.85 (br d, *J*=9.51 Hz, 4 H) 1.30 - 1.58 (m, 12 H)

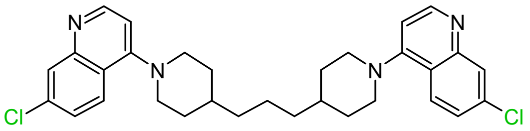

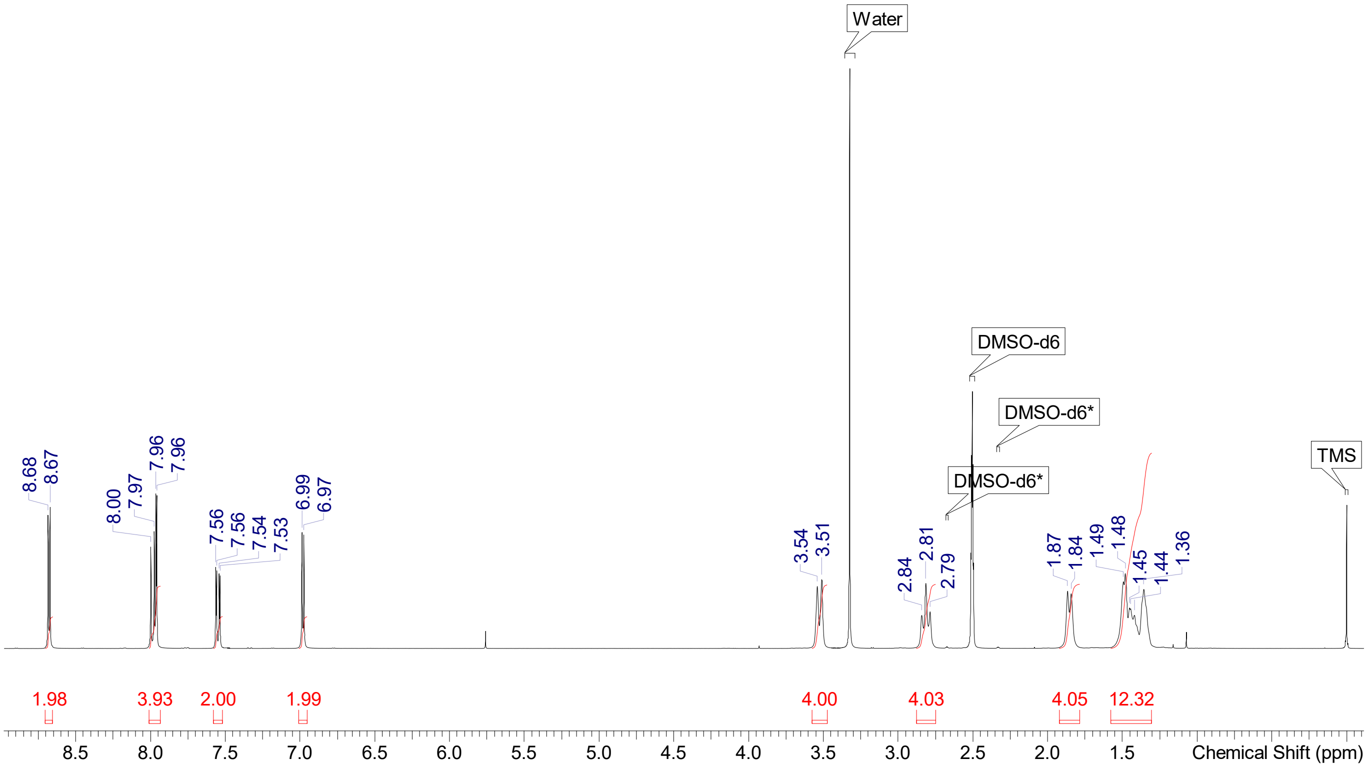

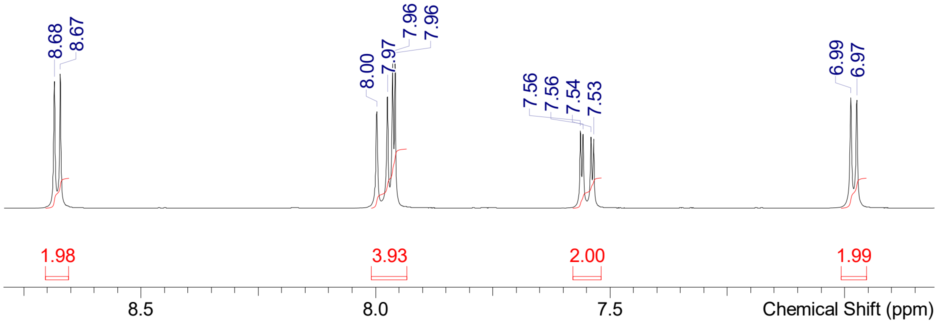

Figure S12: ^{1}H NMR spectra of **21a intermediate**.

^{1}H NMR (400 MHz, DMSO-d_6) δ ppm 9.03 (s, 1 H) 8.44 (d, *J*=5.38 Hz, 1 H) 8.42 (d, *J*=9.26 Hz, 1 H) 7.88 (d, *J*=2.25 Hz, 1 H) 7.56 (dd, *J*=9.01, 2.25 Hz, 1 H) 7.30 (s, 4 H) 6.86 (d, *J*=5.38 Hz, 1 H) 4.09 (br d, *J*=11.76 Hz, 2 H) 2.82 (br s, 1 H) 2.63 - 2.78 (m, 1 H) 1.79 (br d, *J*=12.51 Hz, 2 H) 1.44 - 1.58 (m, 3 H) 1.42 (s, 9 H)

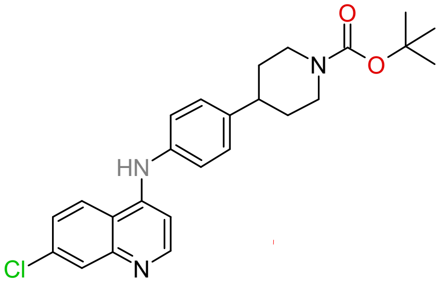

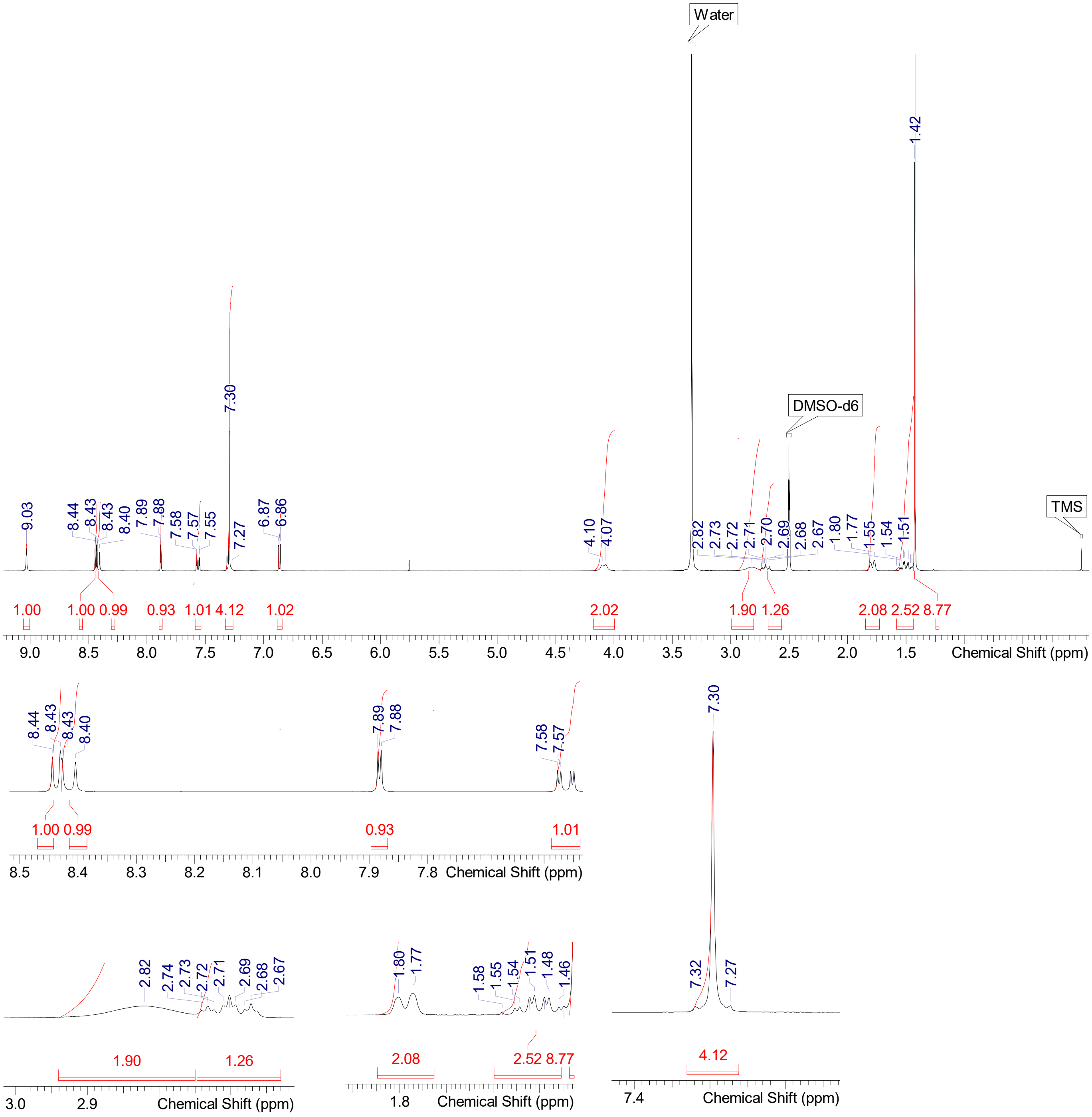

Figure S13: ^{1}H NMR spectra of **21b intermediate**.

^{1}H NMR (400 MHz, CHLOROFORM-*d*) δ ppm 8.49 (d, *J*=5.25 Hz, 1 H) 8.01 (d, *J*=2.13 Hz, 1 H) 7.84 (d, *J*=9.01 Hz, 1 H) 7.44 (dd, *J*=9.01, 2.13 Hz, 1 H) 7.18 - 7.24 (m, 2 H) 6.96 - 7.02 (m, 2 H) 6.71 (d, *J*=5.25 Hz, 1 H) 6.51 (s, 1 H) 3.56 - 3.67 (m, 4 H) 3.12 - 3.21 (m, 5 H) 1.46 - 1.52 (m, 9 H)

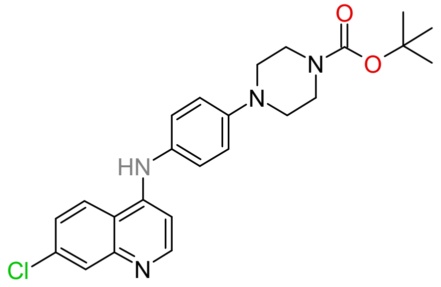

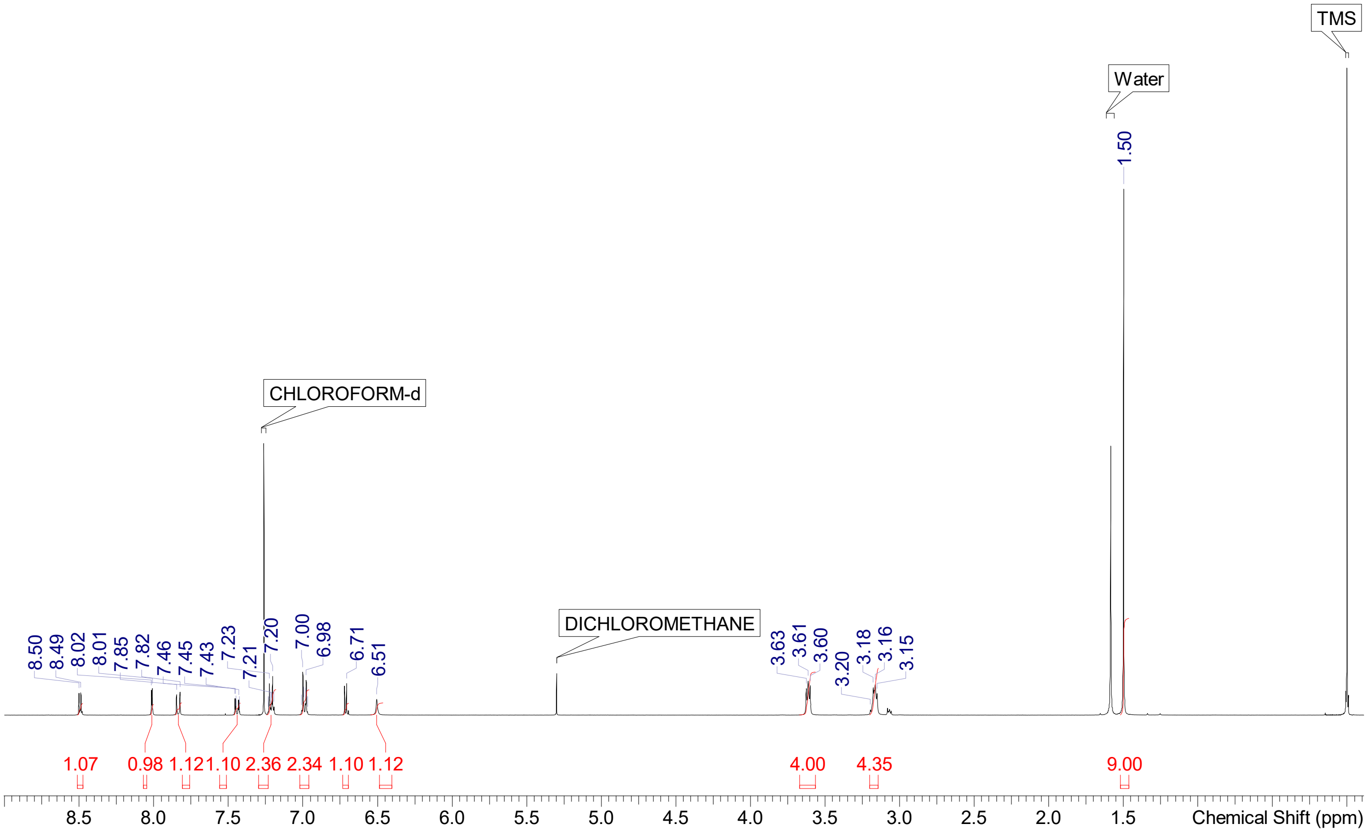

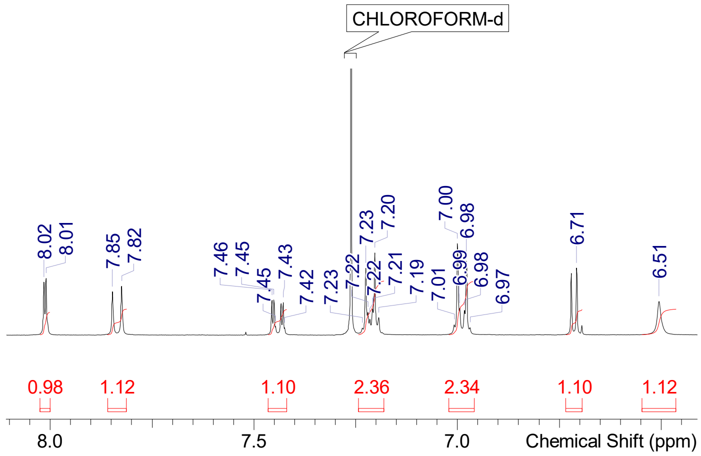

Figure S14: ^{1}H NMR spectra of **21c intermediate**.

^{1}H NMR (400 MHz, DMSO-d_6) δ ppm 8.88 (s, 1 H) 8.43 (d, *J*=5.25 Hz, 1 H) 8.37 (d, *J*=8.50 Hz, 1 H) 7.86 (d, *J*=8.04 Hz, 1 H) 7.68 (t, *J*=7.25 Hz, 1 H) 7.52 (t, *J*=7.26 Hz, 1 H) 7.29 (s, 4 H) 6.87 (d, *J*=5.25 Hz, 1 H) 4.02 - 4.17 (m, 2 H) 3.17 (d, *J*=5.13 Hz, 1 H) 2.82 (br s, 1 H) 2.70 (tt, *J*=11.94, 3.19 Hz, 1 H) 1.79 (br d, *J*=12.26 Hz, 2 H) 1.44 - 1.58 (m, 2 H) 1.43 (s, 9 H)

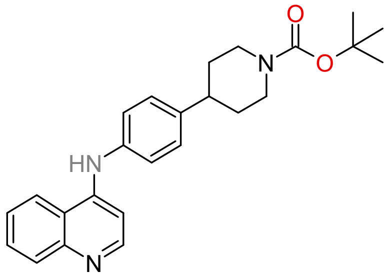

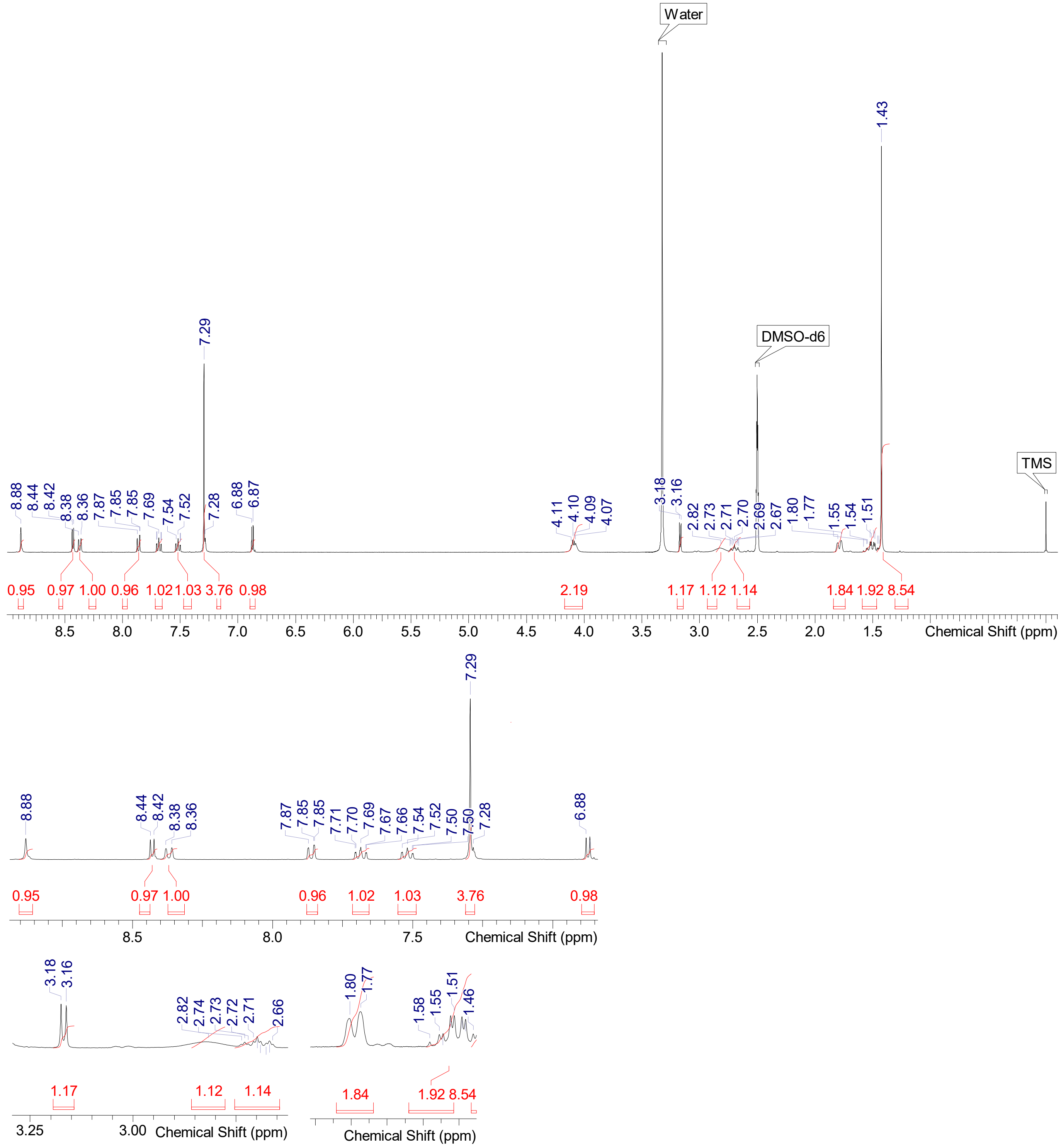

Figure S15: ^{1}H NMR spectra of **21a**.

^{1}H NMR (400 MHz, CHLOROFORM-*d*) δ ppm 8.54 (d, *J*=5.25 Hz, 1 H) 8.03 (d, *J*=2.13 Hz, 1 H) 7.85 (d, *J*=9.01 Hz, 1 H) 7.45 (dd, *J*=8.94, 2.19 Hz, 1 H) 7.27 - 7.31 (m, 2 H) 7.21 - 7.26 (m, 2 H) 6.91 (d, *J*=5.25 Hz, 1 H) 6.58 (s, 1 H) 3.22 (br d, *J*=11.76 Hz, 2 H) 2.77 (td, *J*=12.13, 2.25 Hz, 2 H) 2.65 (s, 1 H) 1.83 - 1.91 (m, 2 H) 1.67 - 1.73 (m, 2 H)

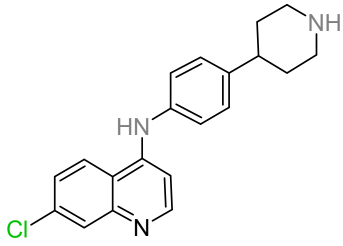

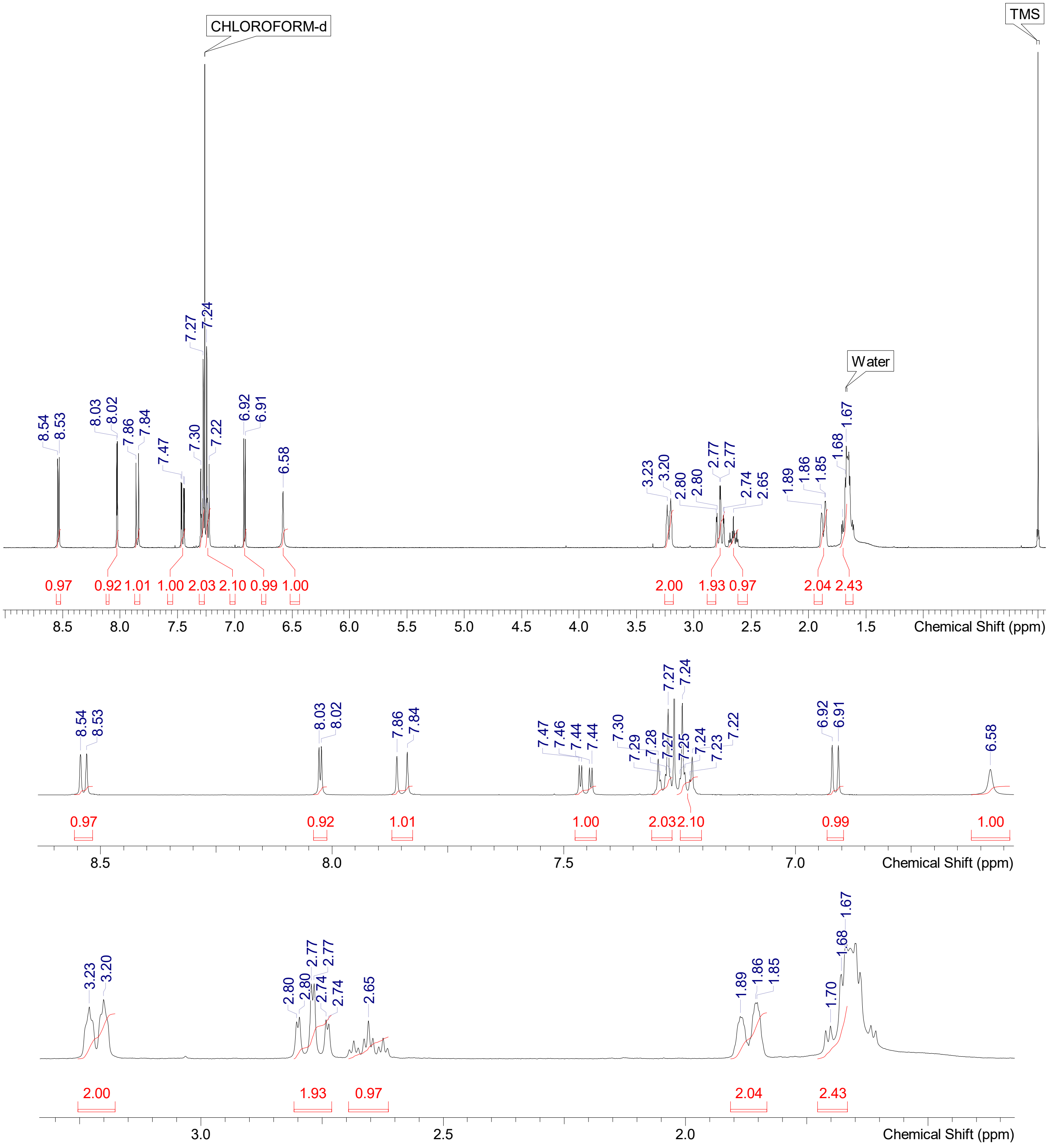

Figure S16: ^{1}H NMR spectra of **21b**.

^{1}H NMR (400 MHz, DMSO-d_6) δ ppm 8.91 (s, 1 H) 8.87 - 8.96 (m, 1 H) 8.41 (d, J=9.01 Hz, 1 H) 8.37 (d, J=5.38 Hz, 1 H) 7.84 (d, J=2.25 Hz, 1 H) 7.52 (dd, J=9.01, 2.25 Hz, 1 H) 7.19 (d, J=8.88 Hz, 2 H) 6.94 - 7.07 (m, 2 H) 6.62 (d, J=5.38 Hz, 1 H) 2.99 - 3.13 (m, 4 H) 2.77 - 2.91 (m, 4 H)

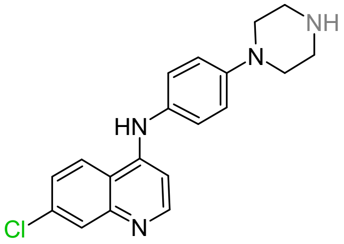

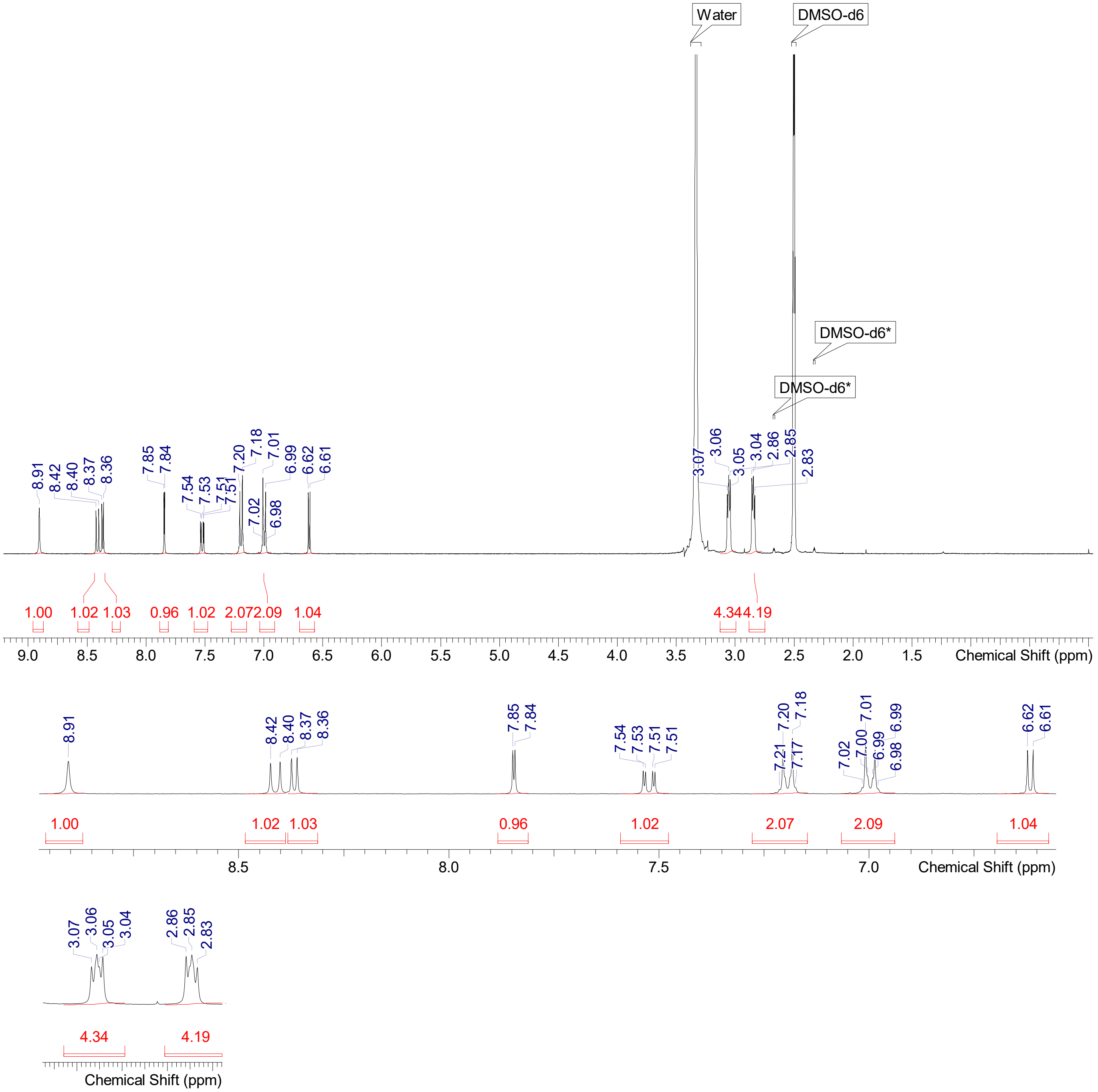

Figure S17: ^{1}H NMR spectra of **21c**.

^{1}H NMR (400 MHz, DMSO-d_6) δ ppm 8.89 (br s, 1 H) 8.43 (d, *J*=5.25 Hz, 1 H) 8.38 (d, *J*=8.38 Hz, 1 H) 7.86 (d, *J*=8.38 Hz, 1 H) 7.69 (t, *J*=7.63 Hz, 1 H) 7.52 (t, *J*=7.63 Hz, 1 H) 7.29 (q, *J*=8.63 Hz, 4 H) 6.87 (d, *J*=5.25 Hz, 1 H) 3.12 - 3.21 (m, 3 H) 2.65 - 2.83 (m, 2 H) 2.00 - 2.09 (m, 1 H) 1.77 - 1.85 (m, 2 H) 1.55 - 1.75 (m, 2 H)

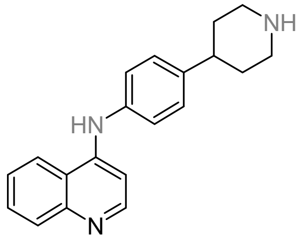

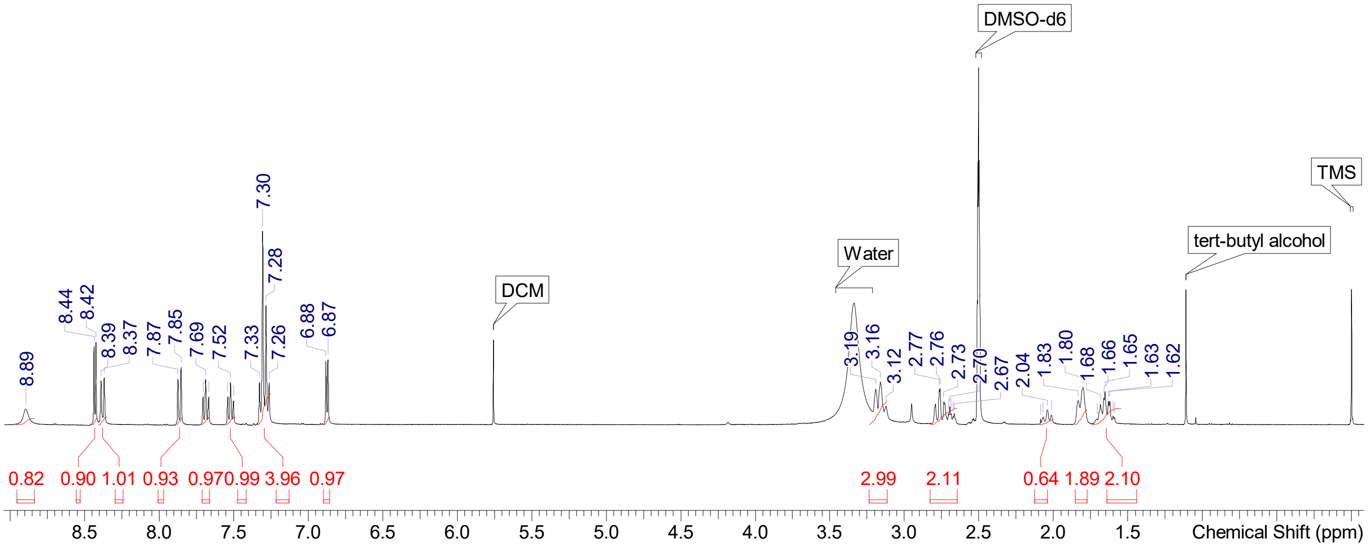

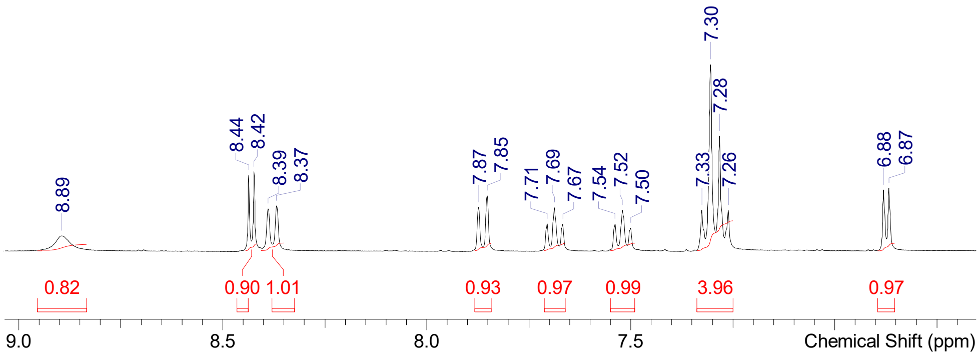

Figure S18: ^{1}H NMR spectra of **23**.

^{1}H NMR (400 MHz, DMSO-d_6) δ ppm 9.04 (s, 1 H) 8.40 - 8.45 (m, 2 H) 7.94 (d, *J*=5.75 Hz, 1 H) 7.88 (d, *J*=2.25 Hz, 1 H) 7.56 (dd, *J*=9.01, 2.25 Hz, 1 H) 7.27 - 7.34 (m, 4 H) 6.86 - 6.91 (m, 3 H) 4.11 (br d, *J*=13.38 Hz, 2 H) 2.95 - 3.05 (m, 2 H) 2.85 (tt, *J*=11.99, 3.39 Hz, 1 H) 1.88 (br d, *J*=11.88 Hz, 2 H) 1.64 (qd, *J*=12.55, 3.63 Hz, 2 H)

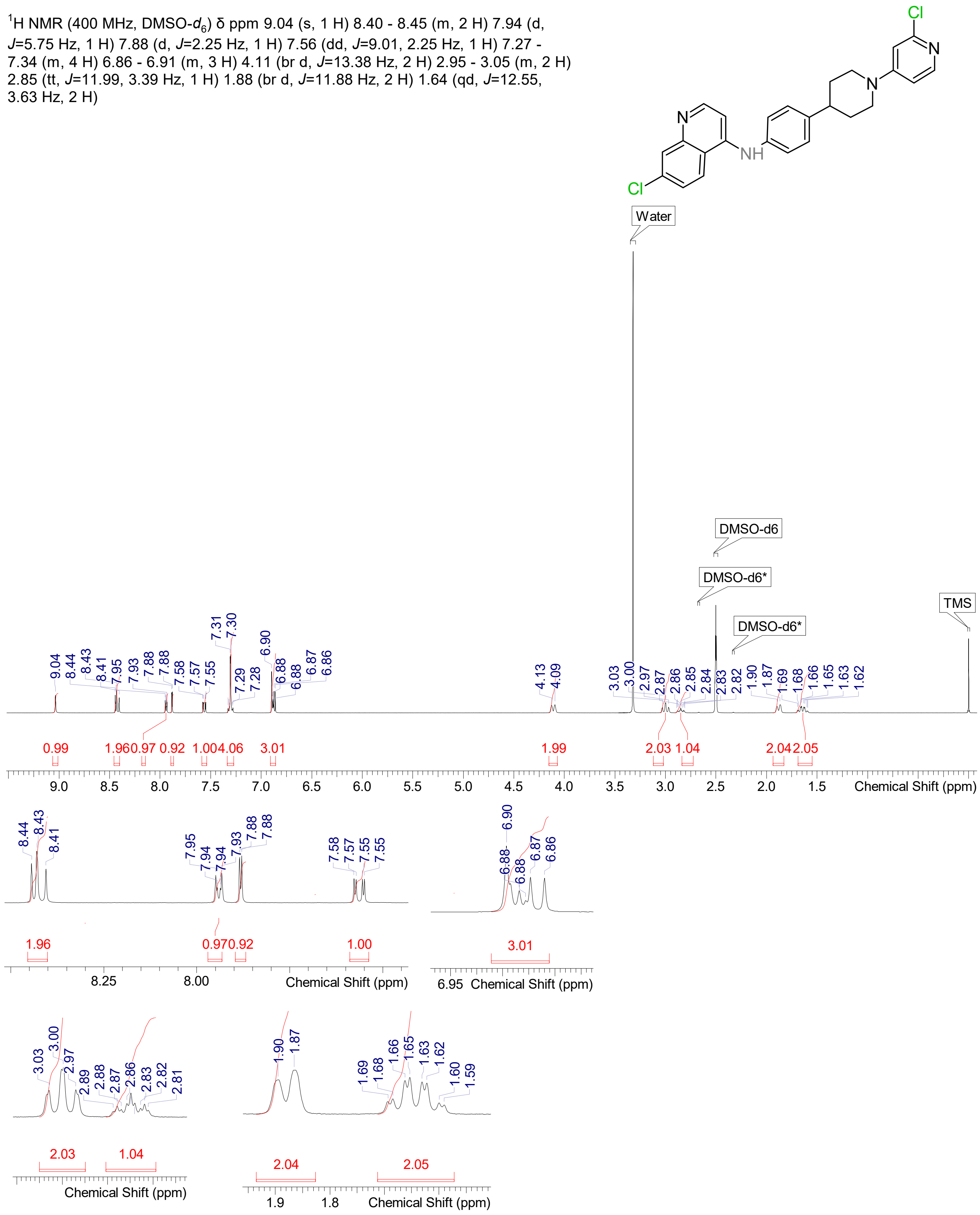

Figure S19: ^{1}H NMR spectra of **24**.

^{1}H NMR (400 MHz, DMSO-d_6) δ ppm 9.07 (s, 1 H) 8.70 (d, J=5.00 Hz, 1 H) 8.42 - 8.48 (m, 2 H) 8.09 (d, J=8.53 Hz, 1 H) 7.96 (dd, J=8.44, 0.81 Hz, 1 H) 7.89 (d, J=2.25 Hz, 1 H) 7.70 (ddd, J=8.32, 6.88, 1.31 Hz, 1 H) 7.53 - 7.61 (m, 2 H) 7.41 - 7.46 (m, 2 H) 7.32 - 7.37 (m, 2 H) 7.03 (d, J=5.00 Hz, 1 H) 6.90 (d, J=5.38 Hz, 1 H) 3.69 (br d, J=12.13 Hz, 2 H) 2.92 - 3.03 (m, 2 H) 2.77 - 2.88 (m, 1 H) 1.97 - 2.11 (m, 4 H)

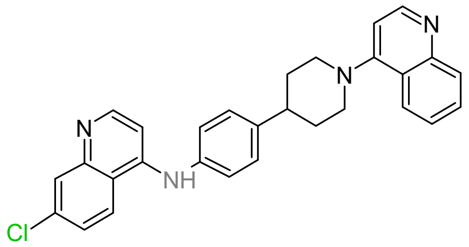

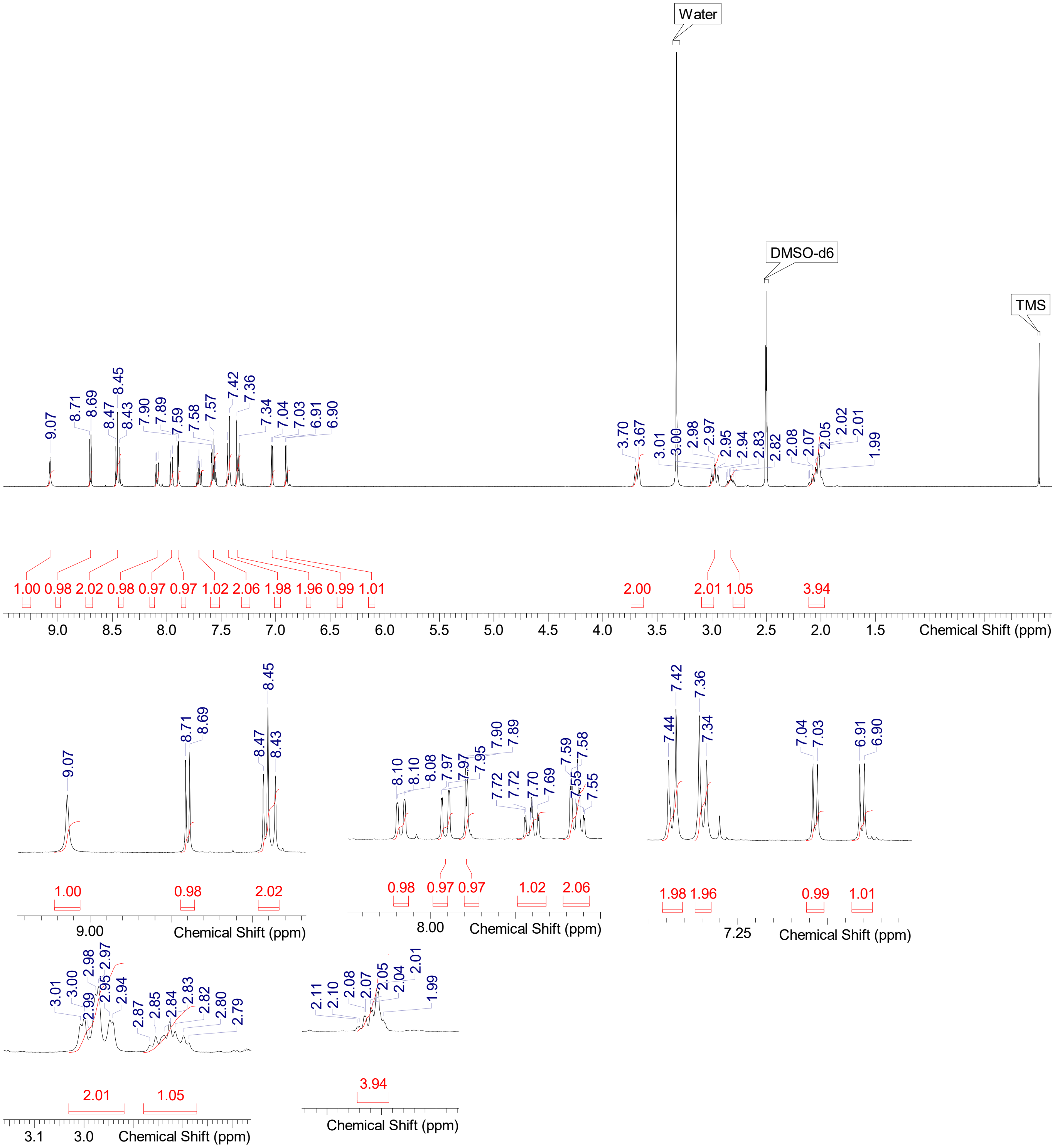

Figure S20: ^{1}H NMR spectra of **25**.

^{1}H NMR (400 MHz, CHLOROFORM-*d*) δ ppm 8.74 (d, *J*=5.00 Hz, 1 H) 8.57 (d, *J*=5.25 Hz, 1 H) 8.05 (t, *J*=2.31 Hz, 2 H) 8.00 (d, *J*=9.01 Hz, 1 H) 7.87 (d, *J*=9.01 Hz, 1 H) 7.46 (t, *J*=9.25 Hz, 2 H) 7.37 - 7.41 (m, 2 H) 7.28 - 7.32 (m, 2 H) 6.96 (d, *J*=5.25 Hz, 1 H) 6.89 (d, *J*=5.00 Hz, 1 H) 6.61 (br s, 1 H) 3.74 (br d, *J*=12.13 Hz, 2 H) 2.94 - 3.05 (m, 2 H) 2.75 - 2.90 (m, 1 H) 2.04 - 2.18 (m, 4 H)

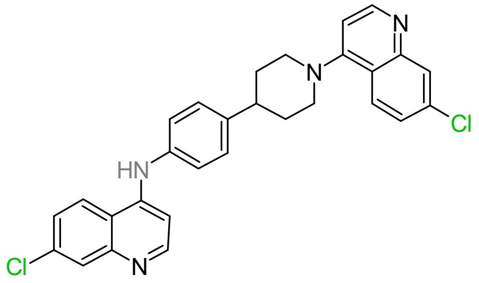

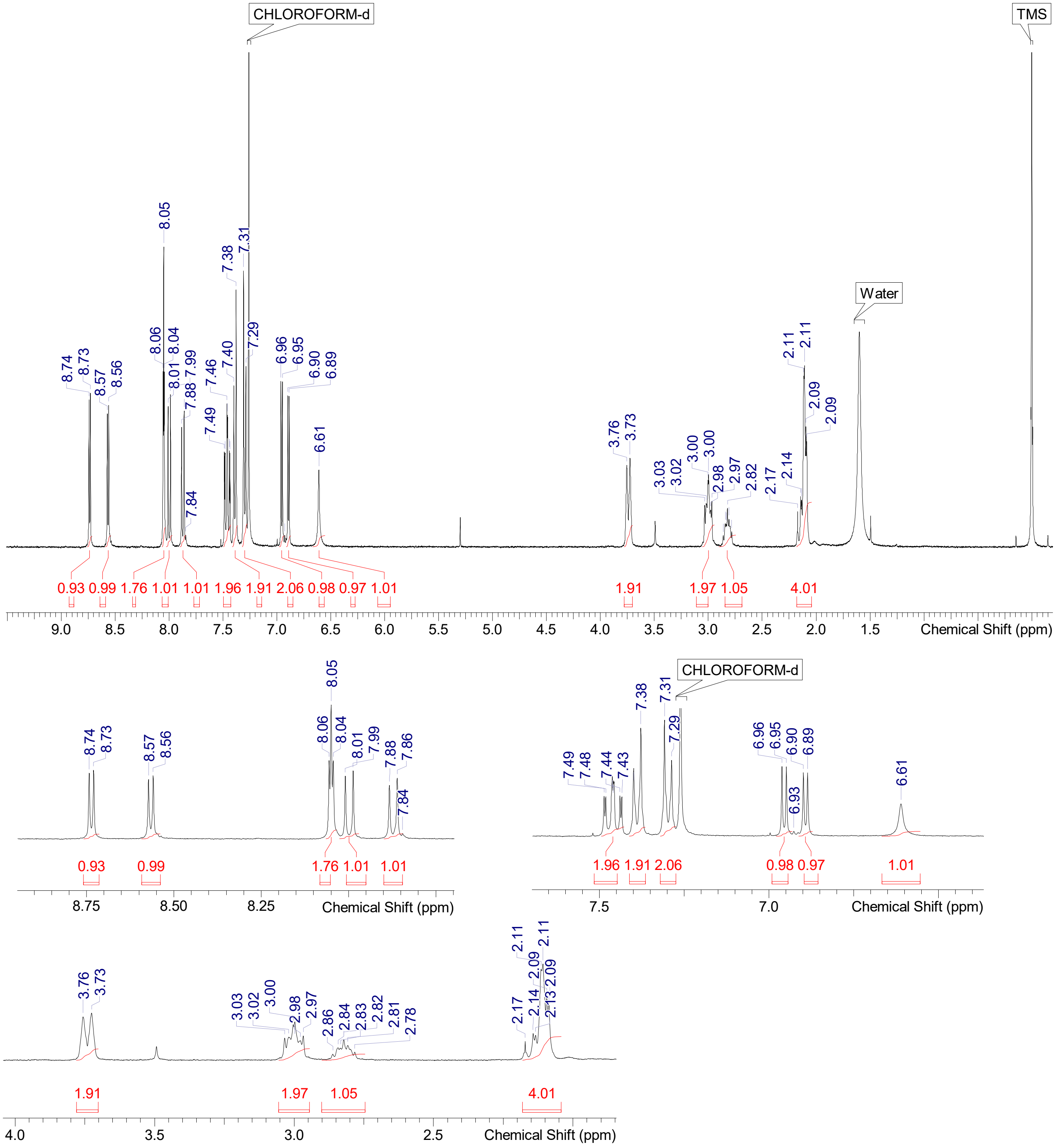

Figure S21: ^{1}H NMR spectra of **26**.

^{1}H NMR (400 MHz, CHLOROFORM-*d*) δ ppm 8.77 (d, *J*=4.88 Hz, 1 H) 8.52 (d, *J*=5.25 Hz, 1 H) 8.08 (d, *J*=2.13 Hz, 1 H) 8.02 (dd, *J*=5.57, 3.44 Hz, 2 H) 7.85 (d, *J*=9.01 Hz, 1 H) 7.46 (ddd, *J*=9.01, 7.00, 2.25 Hz, 2 H) 7.26 - 7.29 (m, 1 H) 7.23 - 7.26 (m, 1 H) 7.06 - 7.11 (m, 2 H) 6.92 (d, *J*=5.13 Hz, 1 H) 6.75 (d, *J*=5.38 Hz, 1 H) 6.54 (s, 1 H) 3.49 - 3.53 (m, 4 H) 3.39 - 3.45 (m, 4 H)

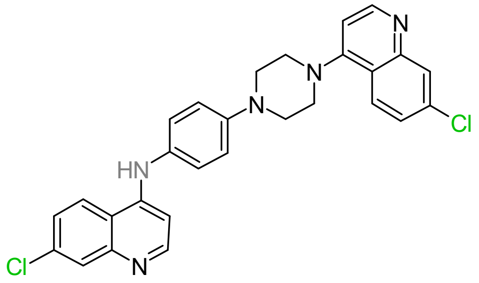

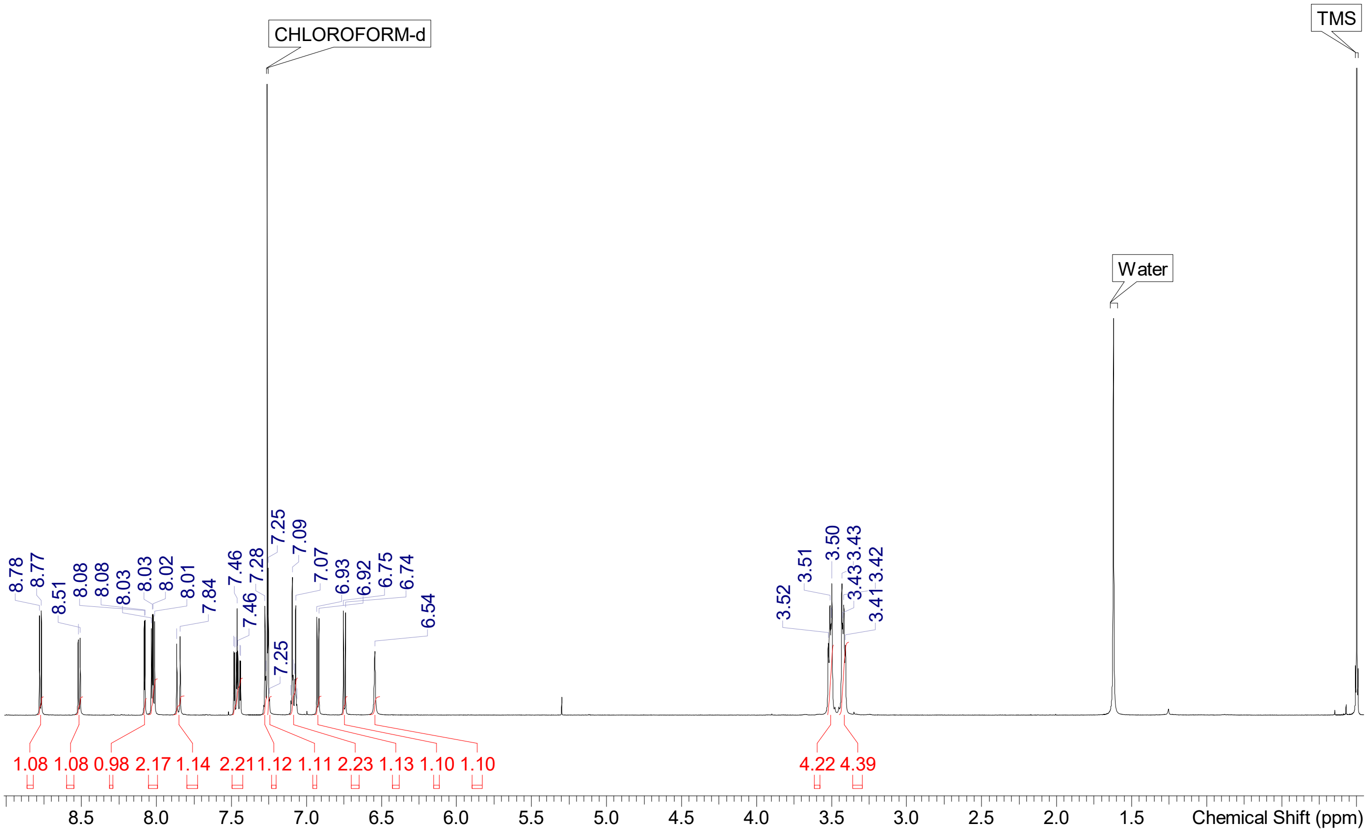

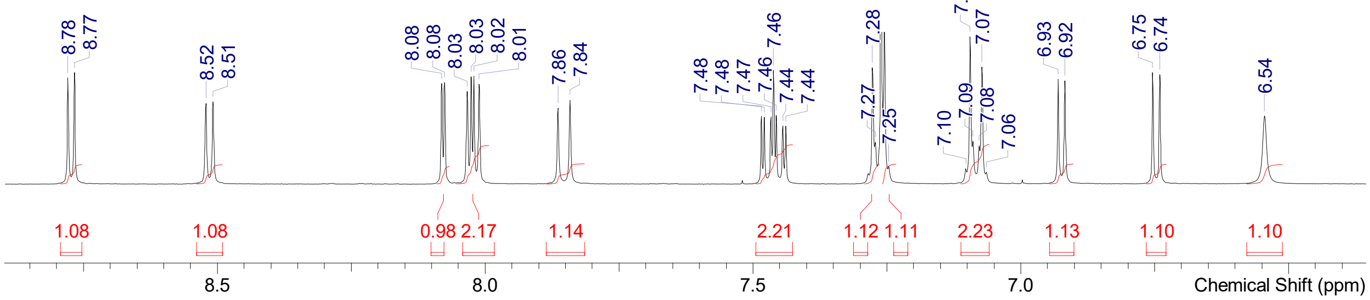

Figure S22: ^{1}H NMR spectra of **27**.

^{1}H NMR (400 MHz, DMSO-d_6) δ ppm 8.92 (s, 1 H) 8.72 (d, *J*=5.00 Hz, 1 H) 8.45 (d, *J*=5.38 Hz, 1 H) 8.39 (d, *J*=8.00 Hz, 1 H) 8.10 (d, *J*=9.01 Hz, 1 H) 7.99 (d, *J*=2.13 Hz, 1 H) 7.87 (d, *J*=7.63 Hz, 1 H) 7.70 (t, *J*=7.14 Hz, 1 H) 7.58 (dd, *J*=9.01, 2.13 Hz, 1 H) 7.53 (t, *J*=7.20 Hz, 1 H) 7.39 - 7.44 (m, 2 H) 7.33 - 7.37 (m, 2 H) 7.06 (d, *J*=5.00 Hz, 1 H) 6.91 (d, *J*=5.25 Hz, 1 H) 3.68 (br d, *J*=12.01 Hz, 2 H) 2.94 - 3.07 (m, 2 H) 2.75 - 2.90 (m, 1 H) 1.97 - 2.11 (m, 4 H)

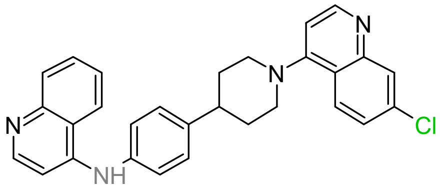

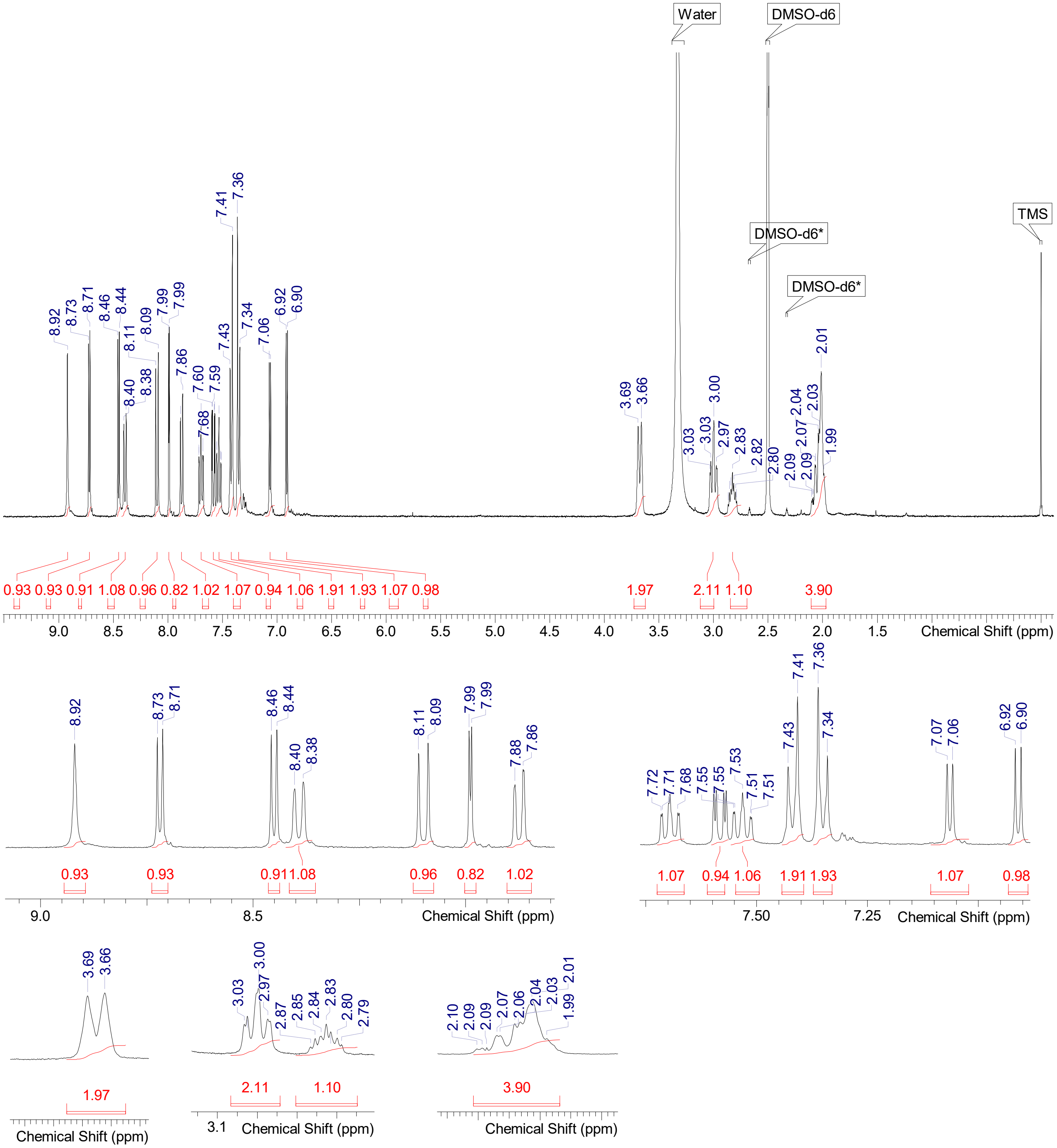

Figure S23: ^{1}H NMR spectra of **28**.

^{1}H NMR (400 MHz, DMSO-d_6) δ ppm 8.93 (s, 1 H) 8.70 (d, *J*=4.88 Hz, 1 H) 8.45 (d, *J*=5.25 Hz, 1 H) 8.40 (d, *J*=8.38 Hz, 1 H) 8.09 (d, *J*=8.38 Hz, 1 H) 7.96 (d, *J*=8.38 Hz, 1 H) 7.88 (d, *J*=8.25 Hz, 1 H) 7.70 (br t, *J*=6.25 Hz, 2 H) 7.55 (tt, *J*=7.88, 7.00 Hz, 2 H) 7.42 (d, *J*=8.25 Hz, 2 H) 7.35 (d, *J*=8.25 Hz, 2 H) 7.04 (d, *J*=5.00 Hz, 1 H) 6.85 - 6.95 (m, 1 H) 3.69 (br d, *J*=11.88 Hz, 2 H) 2.97 (br t, *J*=10.44 Hz, 2 H) 2.75 - 2.90 (m, 1 H) 1.98 - 2.11 (m, 4 H)

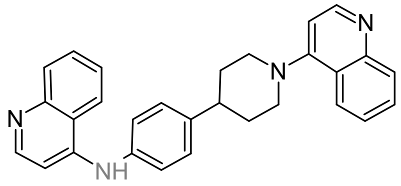

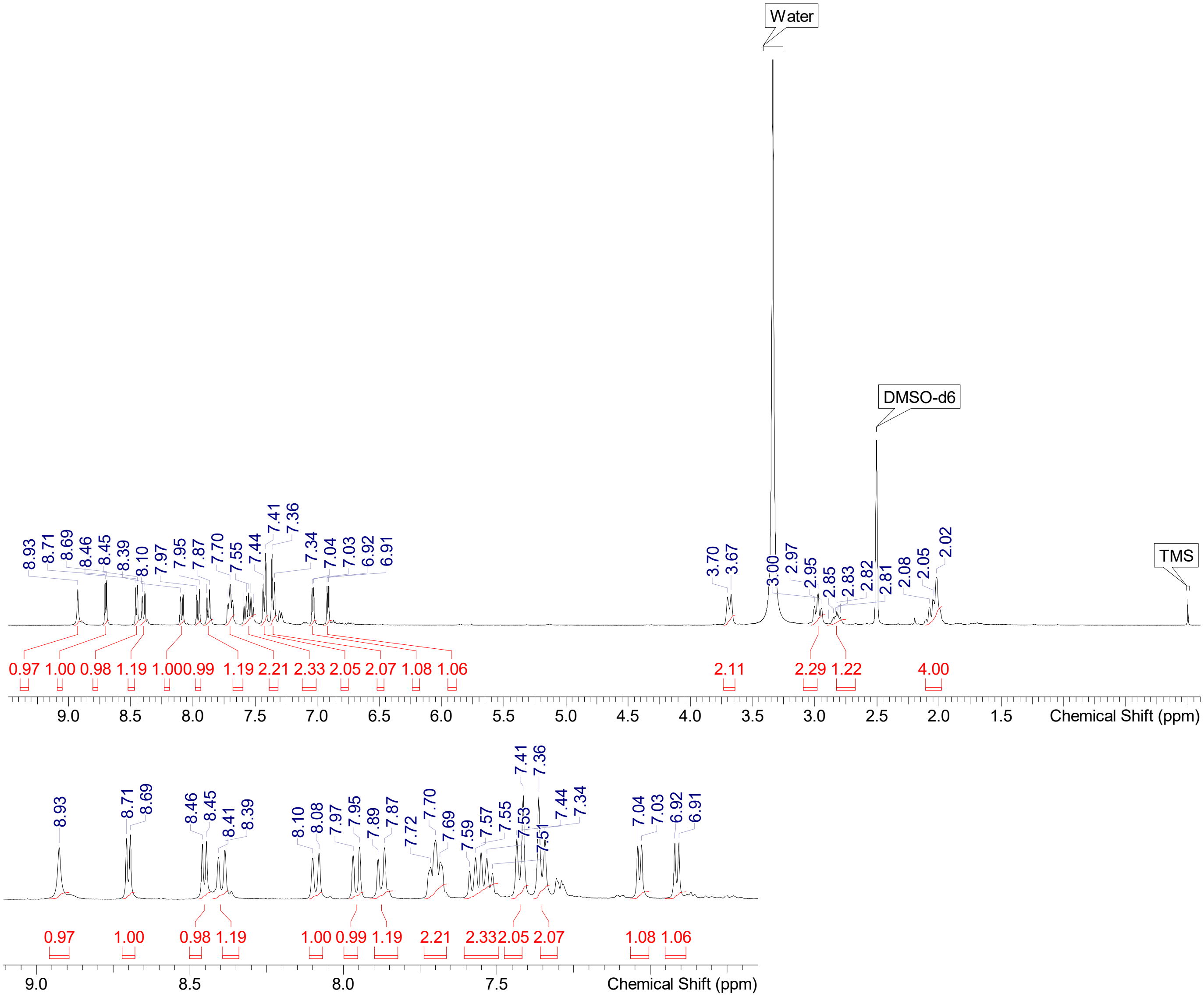

HRAM/MS Chromatograms of Presented Compounds

Figure S24: HRAM/MS Chromatogram of **8**.

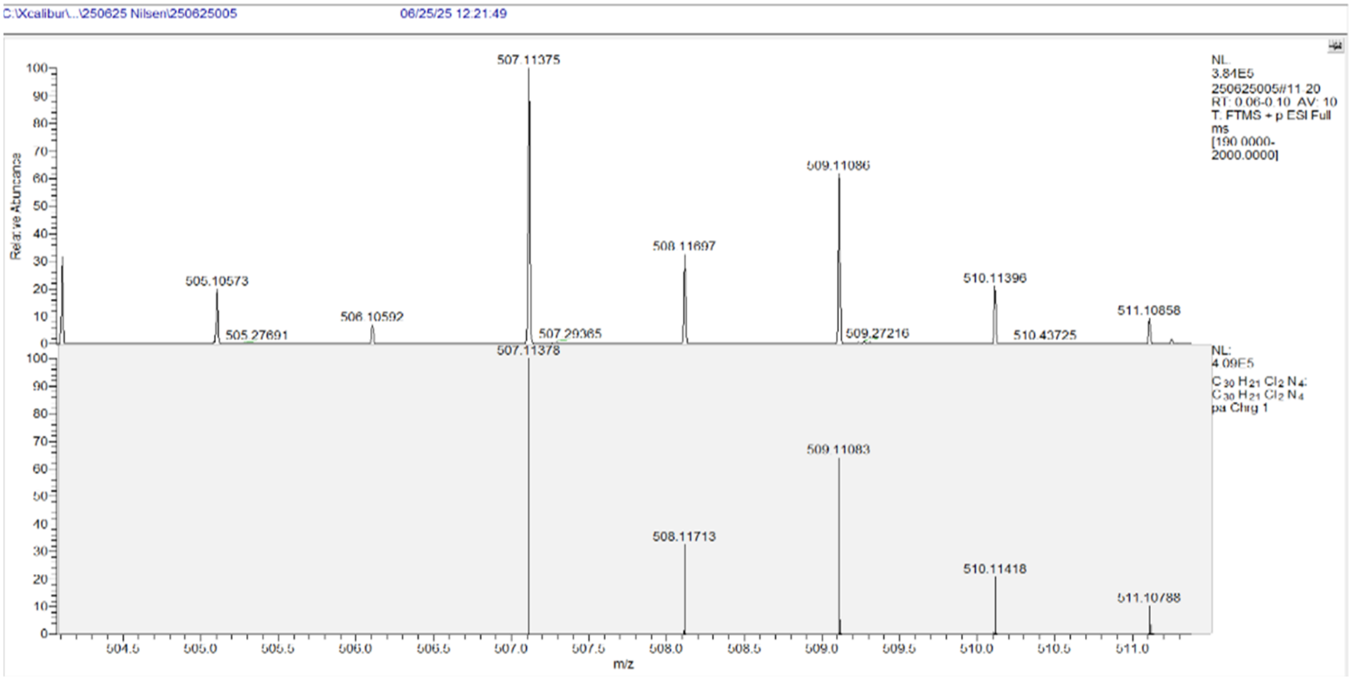

High Resolution Accurate Mass/Mass Spectrometry using positive mode ESI: $[M+H]^+$ of $[C_{30}H_{20}Cl_2N_4]$ = 507.11377 amu (top); observed = 507.11375 amu (bottom).

Figure S25: HRAM/MS Chromatogram of **9**.

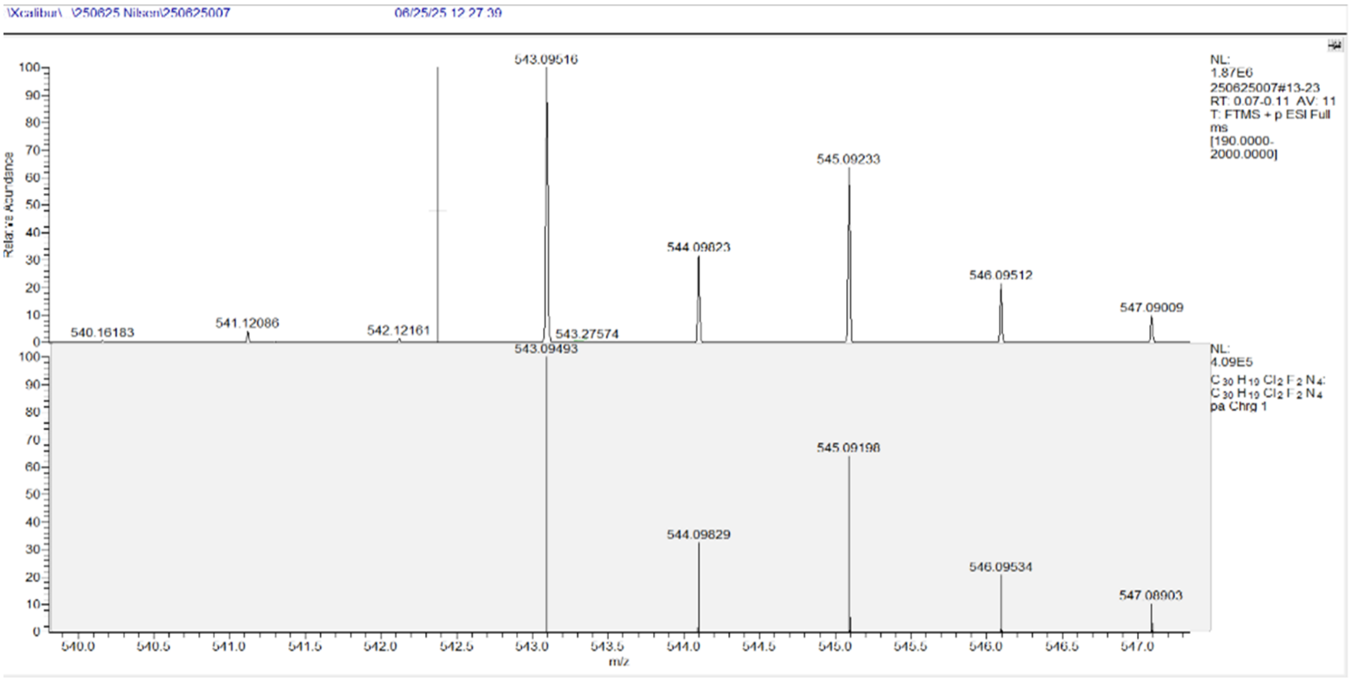

High Resolution Accurate Mass/Mass Spectrometry using positive mode ESI: $[M+H]^+$ of $[C_{30}H_{18}Cl_2F_2N_4]$ = 543.09493 amu (top); observed = 543.09516 (bottom).

Figure S26: HRAM/MS Chromatogram of **10**.

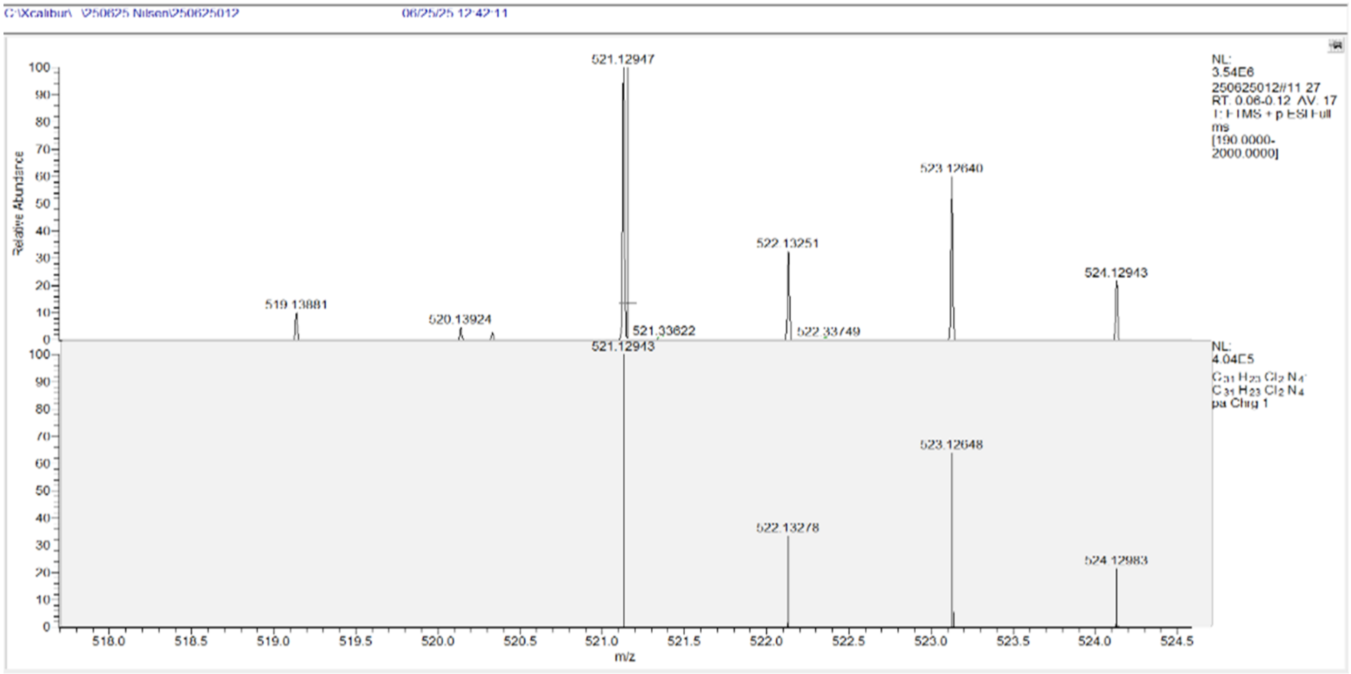

High Resolution Accurate Mass/Mass Spectrometry using positive mode ESI: $[M+H]^+$ of $[C_{31}H_{22}Cl_2N_4]$ = 521.12942 amu (top); observed = 521.12947 (bottom).

Figure S27: HRAM/MS Chromatogram of **11**.

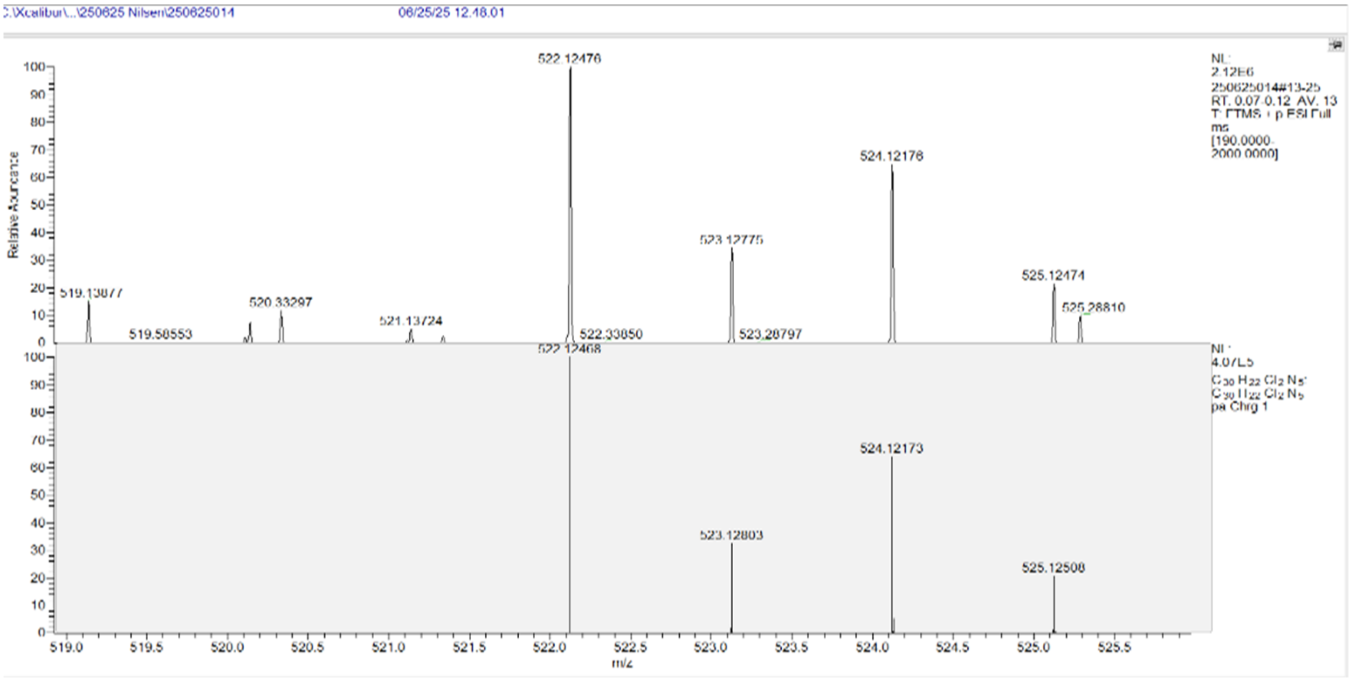

High Resolution Accurate Mass/Mass Spectrometry using positive mode ESI: $[M+H]^+$ of $[C_{30}H_{21}Cl_2N_5]$ = 522.12467 amu (top); observed = 522.12476 (bottom).

Figure S28: HRAM/MS Chromatogram of **12**.

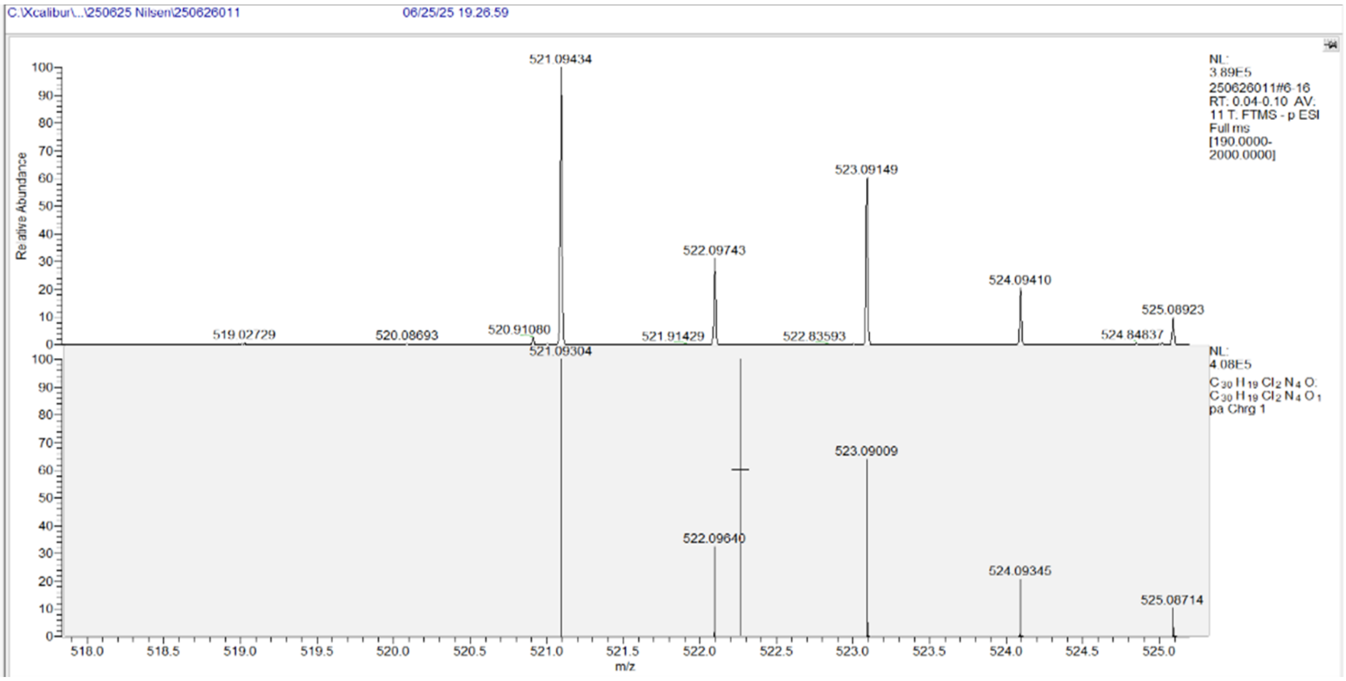

High Resolution Accurate Mass/Mass Spectrometry using negative mode ESI: [M-H]$^-$ of [$C_{30}H_{20}Cl_2N_4O$] = 521.09304 amu (top); observed = 521.09434 amu (bottom).

Figure S29: HRAM/MS Chromatogram of **13**.

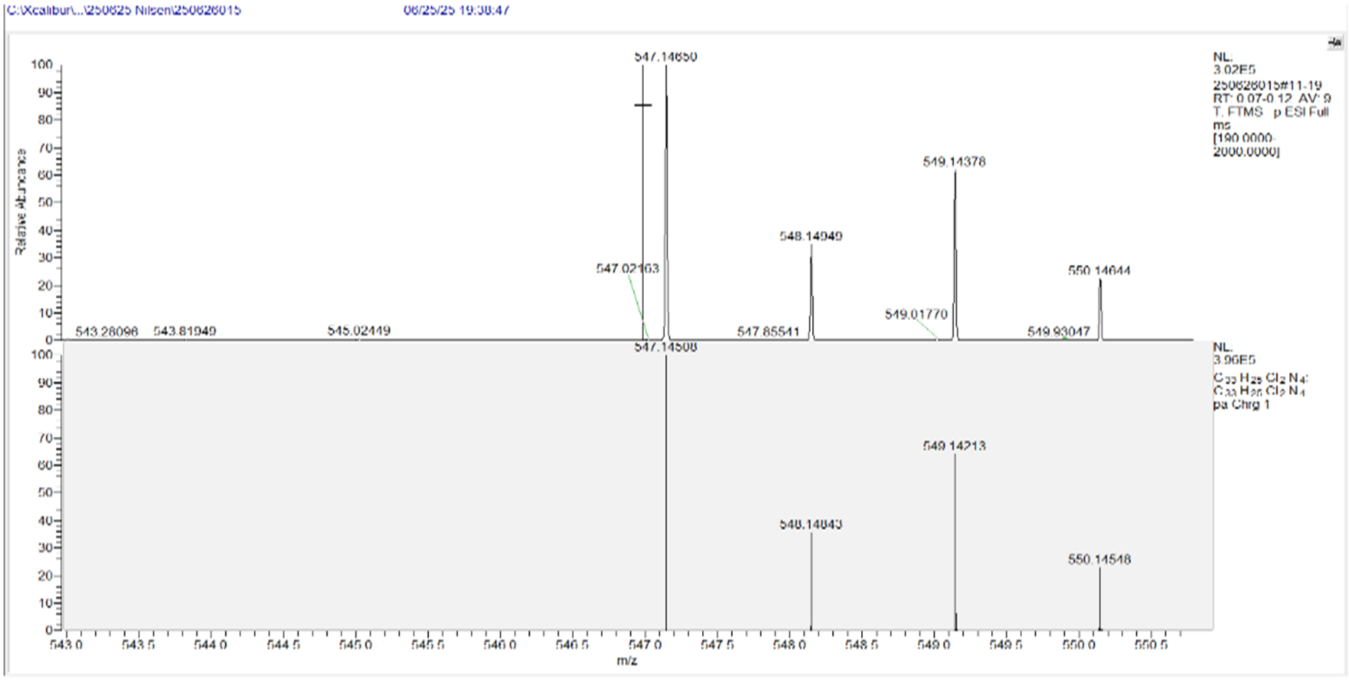

High Resolution Accurate Mass/Mass Spectrometry using negaitive mode ESI: $[M\text{-}H]^-$ of $[C_{33}H_{26}Cl_2N_4]$ = 547.14507 amu (top); observed = 547.14650 (bottom).

Figure S30: HRAM/MS Chromatogram of **14**.

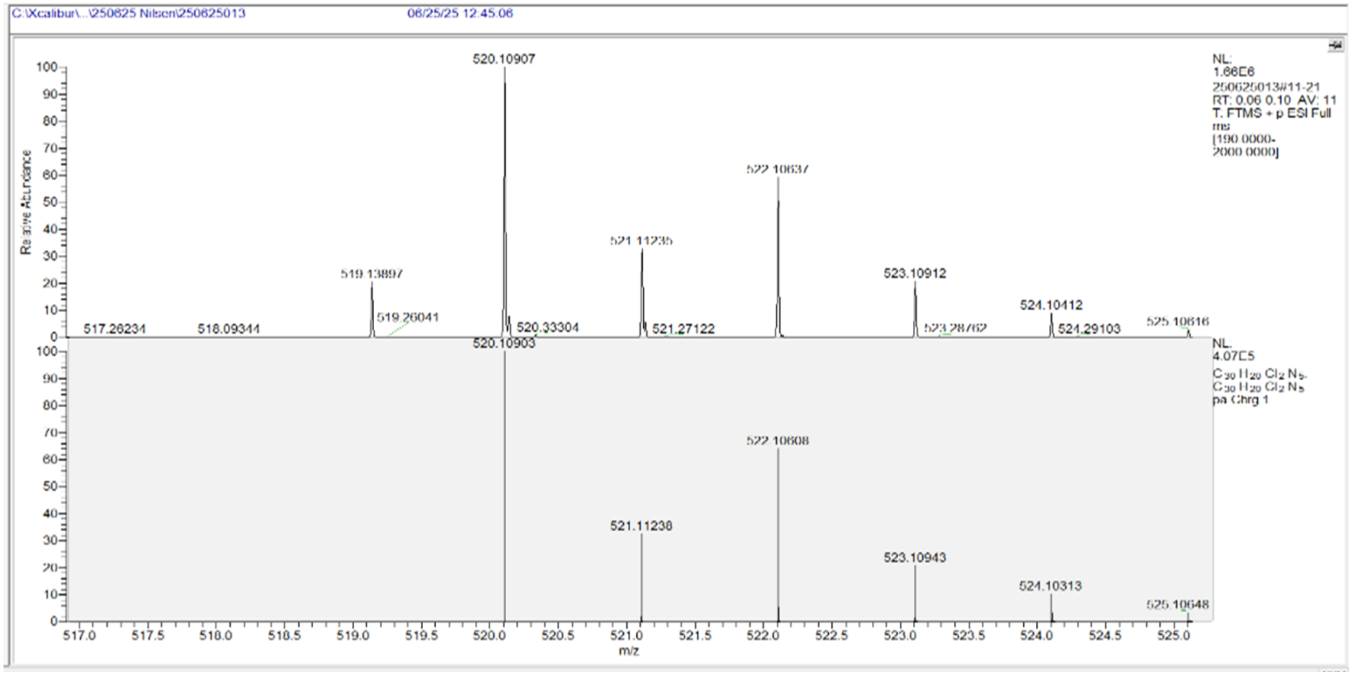

High Resolution Accurate Mass/Mass Spectrometry using positive mode ESI: $[M+H]^+$ of $[C_{30}H_{19}Cl_2N_5]$ = 520.10907 amu (top); observed = 520.10907 amu (bottom).

Figure S31: HRAM/MS Chromatogram of **15**.

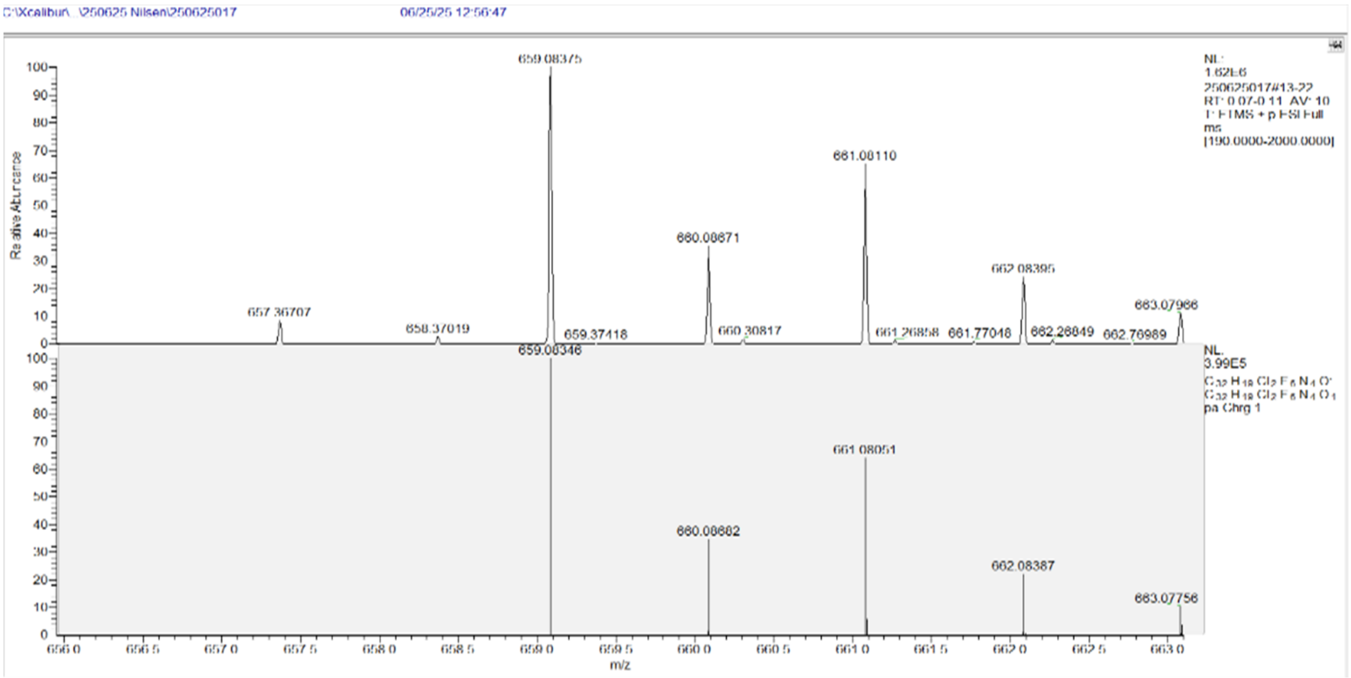

High Resolution Accurate Mass/Mass Spectrometry using positive mode ESI: $[M+H]^+$ of $[C_{32}H_{18}Cl_2F_6N_4O]$ = 659.08375 amu (top); observed = 659.08375 amu (bottom).

Figure S32: HRAM/MS Chromatogram of **17**.

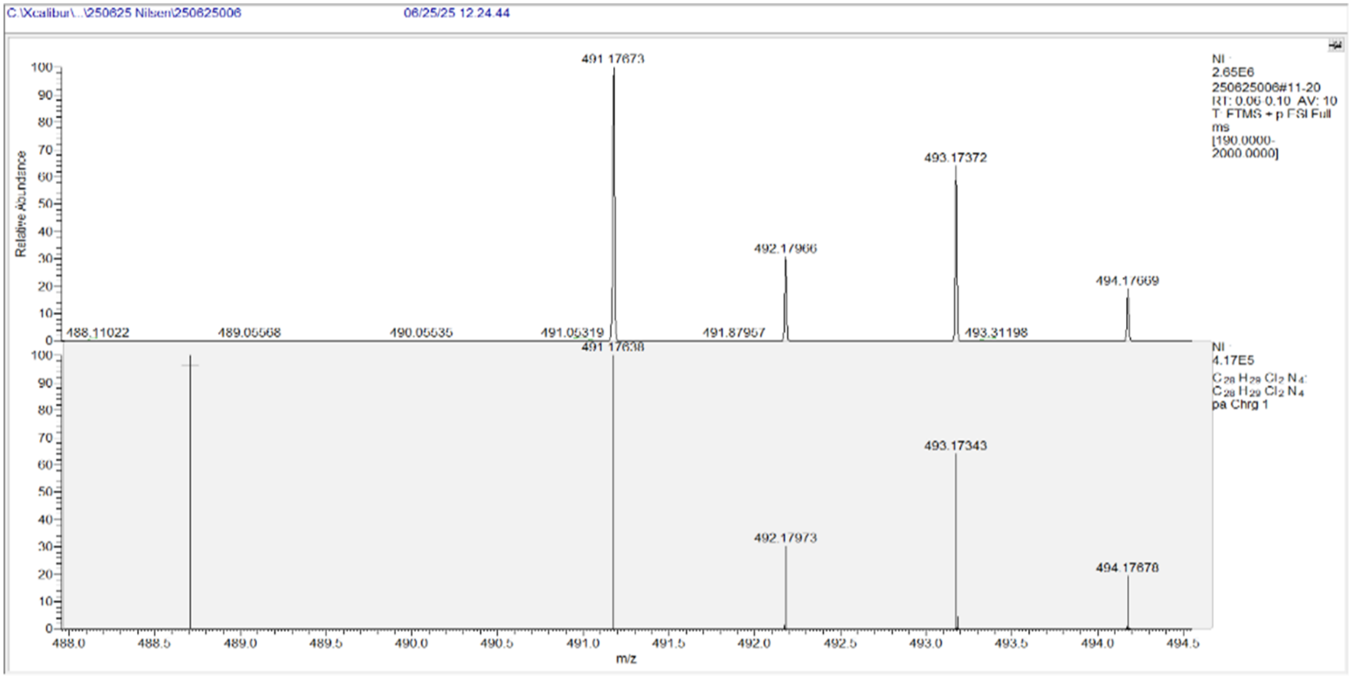

High Resolution Accurate Mass/Mass Spectrometry using positive mode ESI: $[M+H]^+$ of $[C_{28}H_{28}Cl_2N_4]$ = 491.17637 amu (top); observed = 491.17673 amu (bottom).

Figure S33: HRAM/MS Chromatogram of **18**.

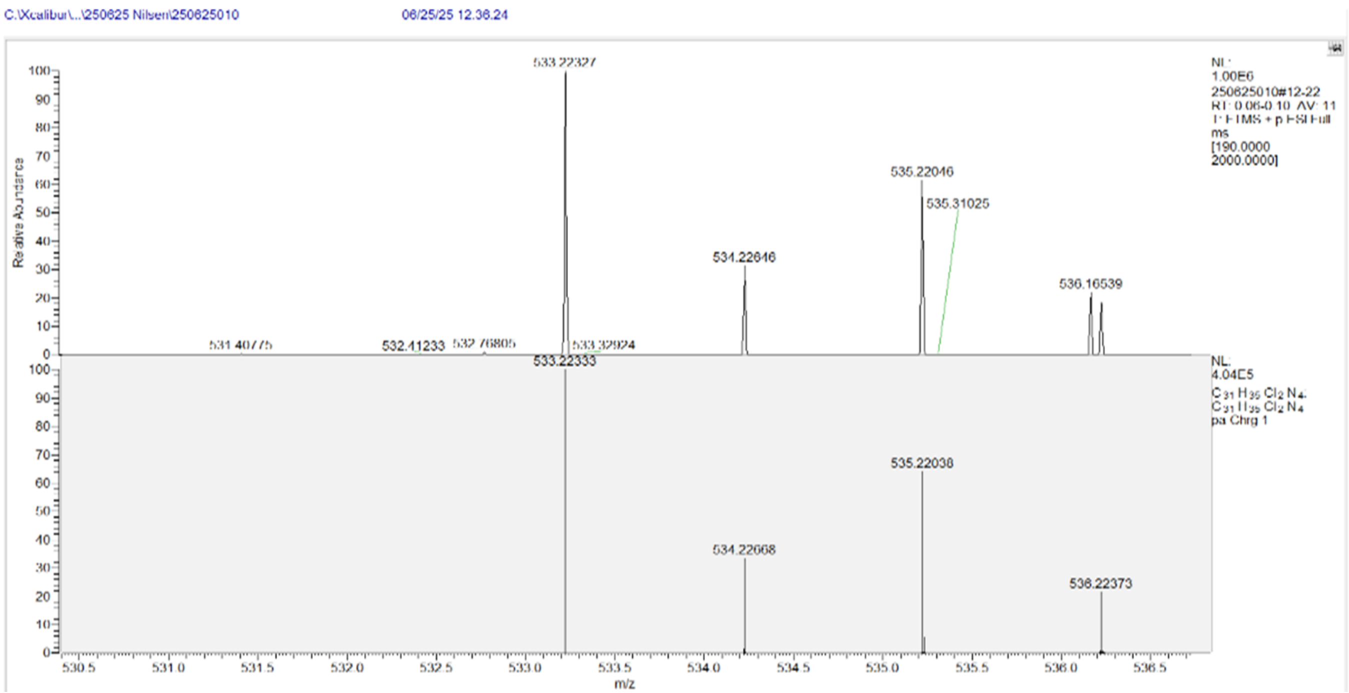

High Resolution Accurate Mass/Mass Spectrometry using positive mode ESI: $[M+H]^+$ of $[C_{31}H_{34}Cl_2N_4]$ = 533.22332 amu (top); observed = 533.22325 amu (bottom).

Figure S34: HRAM/MS Chromatogram of **23**.

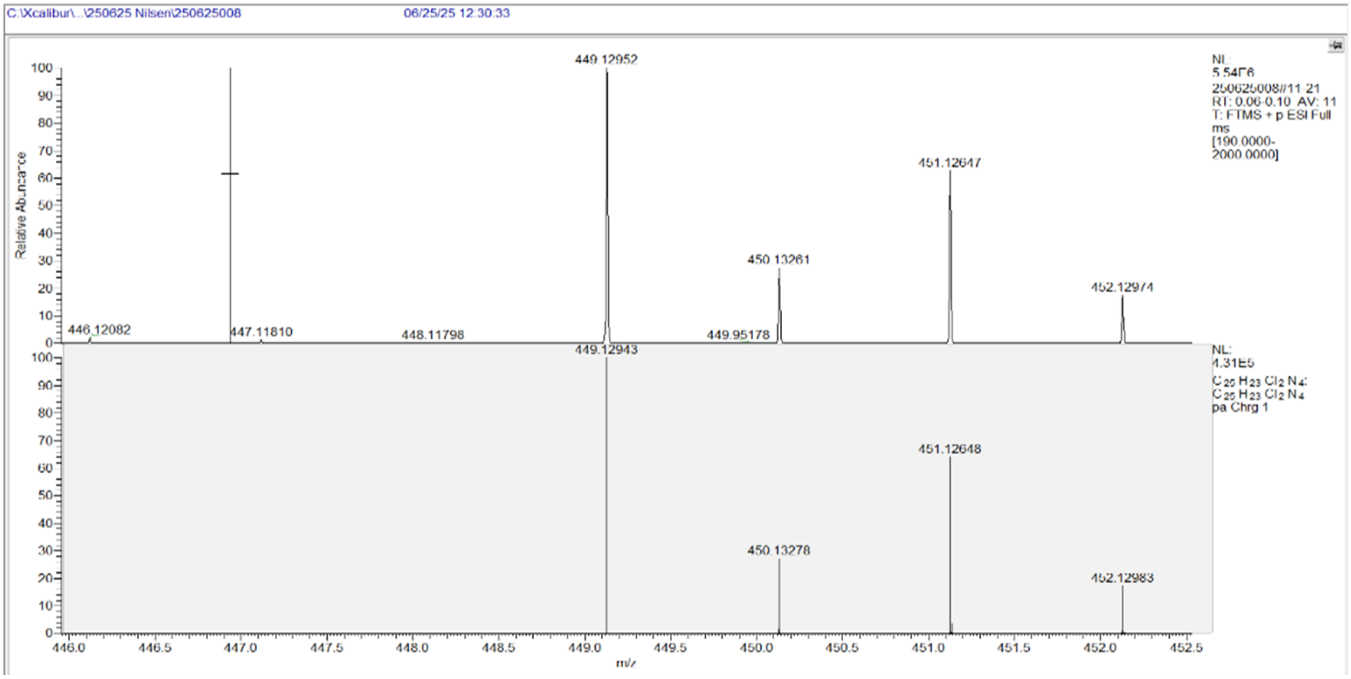

High Resolution Accurate Mass/Mass Spectrometry using positive mode ESI: $[M+H]^+$ of $[C_{25}H_{22}Cl_2N_4]$ = 449.12942 amu (top); observed = 449.12952 amu (bottom).

Figure S35: HRAM/MS Chromatogram of **24**.

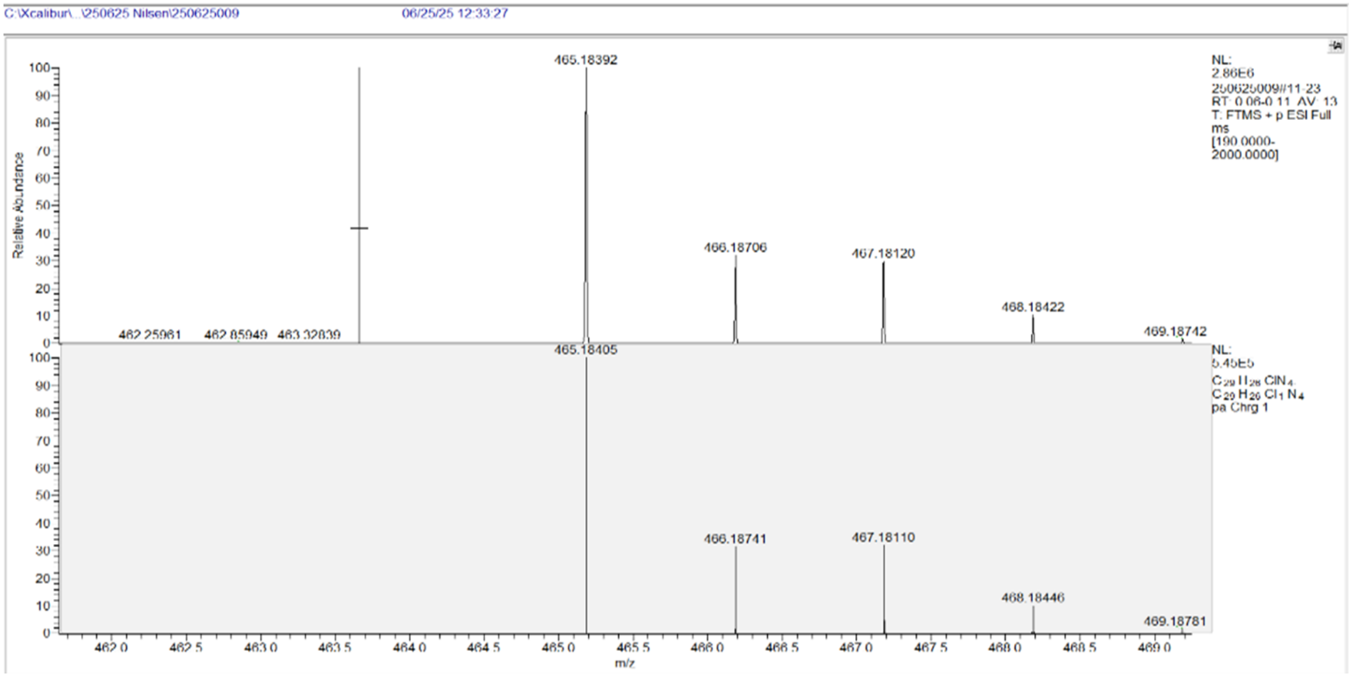

High Resolution Accurate Mass/Mass Spectrometry using positive mode ESI: $[M+H]^+$ of $[C_{29}H_{25}ClN_4]$ = 465.18405 amu (top); observed = 465.18392 amu (bottom).

Figure S36: HRAM/MS Chromatogram of **25**.

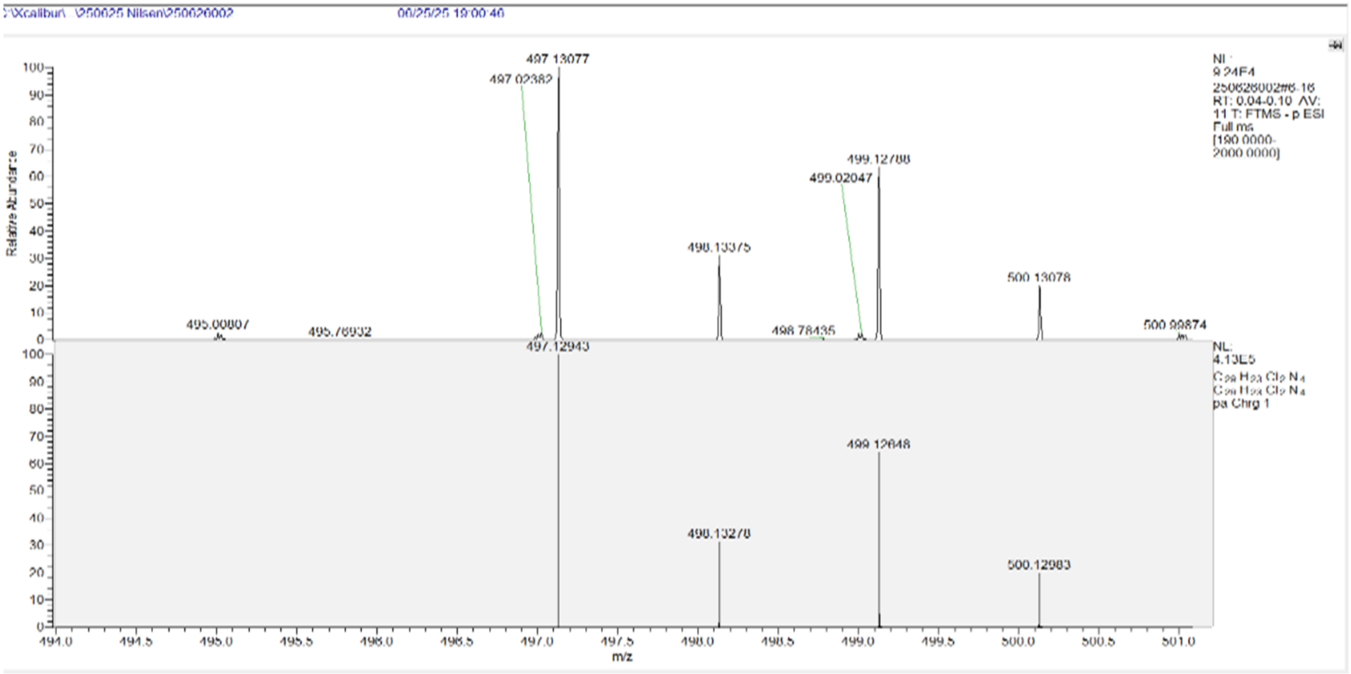

High Resolution Accurate Mass/Mass Spectrometry using negative mode ESI: [M-H]$^-$ of [$C_{29}H_{24}Cl_2N_4$] = 497.12942 amu (top); observed = 497.13077 amu (bottom).

Figure S37: HRAM/MS Chromatogram of **26**.

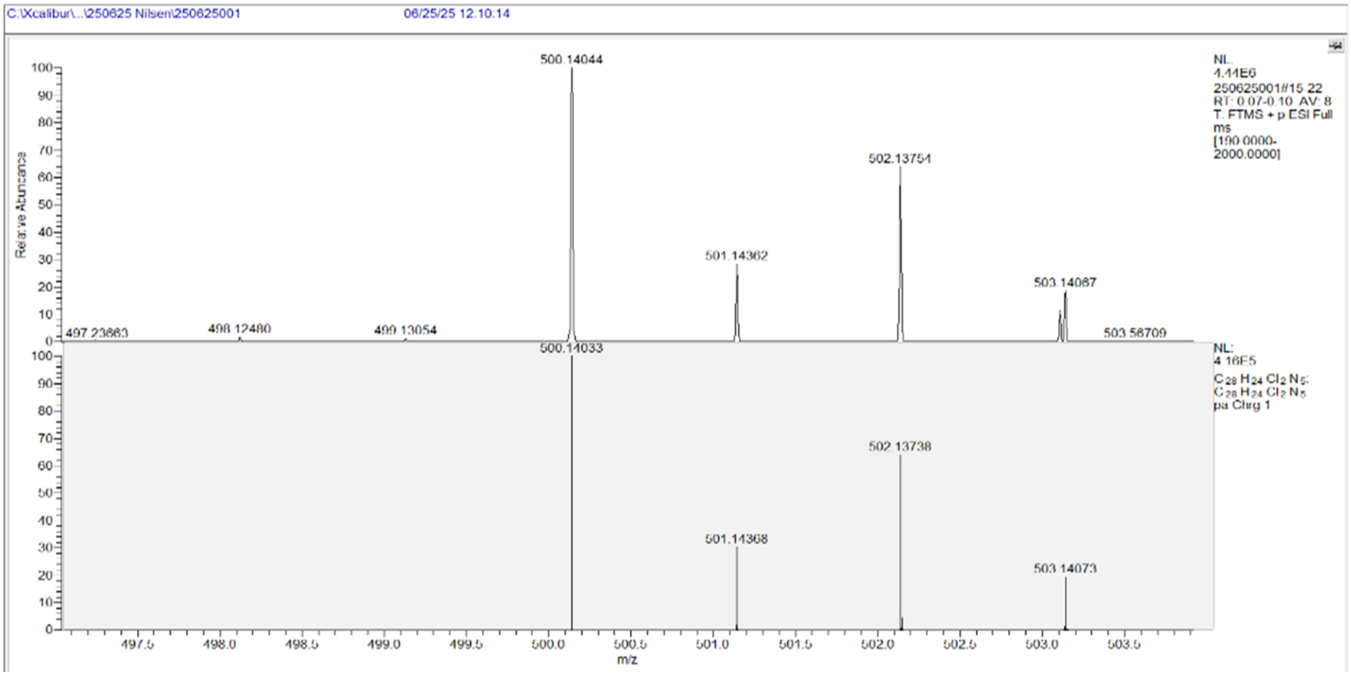

High Resolution Accurate Mass/Mass Spectrometry using positive mode ESI: $[M+H]^+$ of $[C_{28}H_{23}Cl_2N_5]$ = 500.14032 amu (top); observed = 500.14042 amu (bottom).

Figure S38: HRAM/MS Chromatogram of **27**.

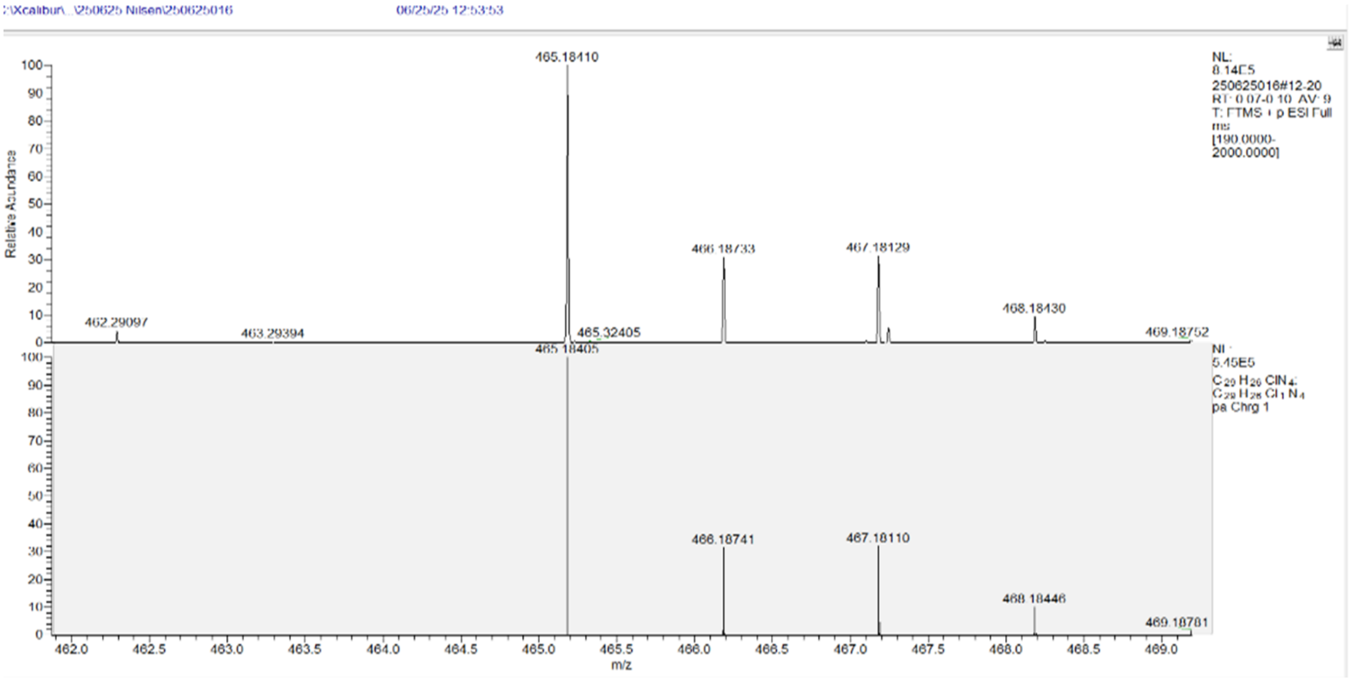

High Resolution Accurate Mass/Mass Spectrometry using positive mode ESI: $[M+H]^+$ of $[C_{29}H_{25}ClN_4]$ = 465.18405 amu (top); observed = 465.18410 amu (bottom).

Figure S39: HRAM/MS Chromatogram of **28**.

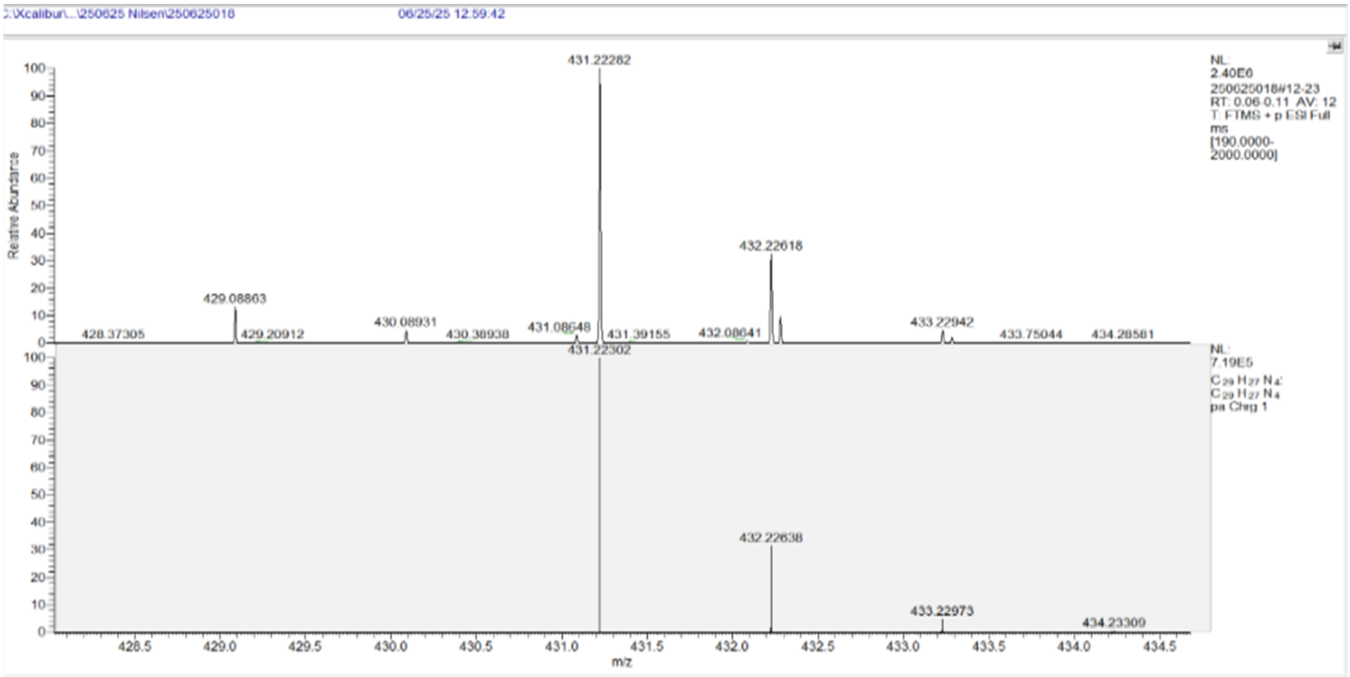

High Resolution Accurate Mass/Mass Spectrometry using positive mode ESI: $[M+H]^+$ of $[C_{29}H_{26}N_4]$ = 431.22302 amu (top); observed = 431.22282 amu (bottom).
