# Supplementary figures and images for: Designing novel bisquinoline antimalarials from historical 4-aminoquinolines to combat drug-resistant malaria
Source: Antimicrob Agents Chemother. 2026 Mar 2;70(4):e01300-25. doi: 10.1128/aac.01300-25 (PMC13041310; doi:10.1128/aac.01300-25)

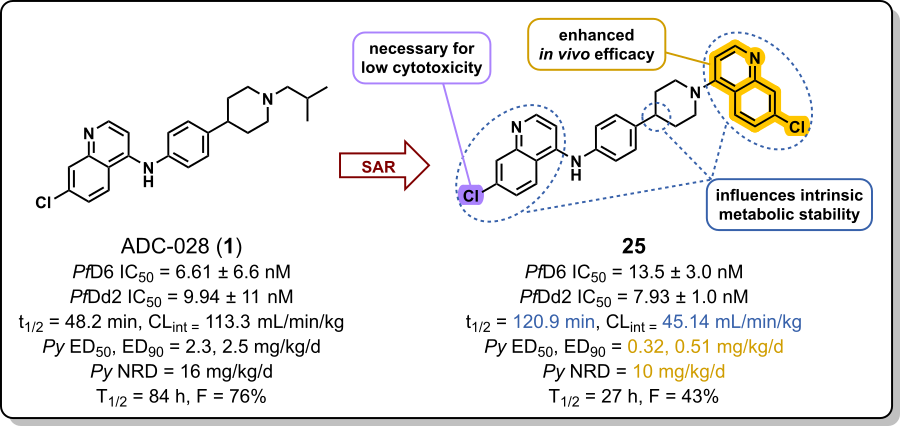

Supplement: Graphical abstract — Visual depiction of the study. [file aac.01300-25-s0002.tiff]
